# Through Massage to the Brain—Neuronal and Neuroplastic Mechanisms of Massage Based on Various Neuroimaging Techniques (EEG, fMRI, and fNIRS)

**DOI:** 10.3390/jcm15020909

**Published:** 2026-01-22

**Authors:** James Chmiel, Donata Kurpas

**Affiliations:** 1 Institute of Neurofeedback and tDCS Poland, ul. 3 Maja 25-27, 70-215 Szczecin, Poland; 2 Department of Nursing, Faculty of Nursing and Midwifery, Division of Research Methodology, Wrocław Medical University, 51-618 Wrocław, Poland

**Keywords:** massage, Tuina, electroencephalography, electroencephalogram, EEG, QEEG, neurophysiology, neural correlates, oscillations, fMRI, fNIRS

## Abstract

**Introduction**: Massage therapy delivers structured mechanosensory input that can influence brain function, yet the central mechanisms and potential for neuroplastic change have not been synthesized across neuroimaging modalities. This mechanistic review integrates evidence from electroencephalography (EEG), functional MRI (fMRI), and functional near-infrared spectroscopy (fNIRS) to map how massage alters human brain activity acutely and over time and to identify signals of longitudinal adaptation. **Materials and Methods**: We conducted a scoping, mechanistic review informed by PRISMA/PRISMA-ScR principles. PubMed/MEDLINE, Cochrane Library, Google Scholar, and ResearchGate were queried for English-language human trials (January 1990–July 2025) that (1) delivered a practitioner-applied manual massage (e.g., Swedish, Thai, shiatsu, tuina, reflexology, myofascial techniques) and (2) measured brain activity with EEG, fMRI, or fNIRS pre/post or between groups. Non-manual stimulation, structural-only imaging, protocols, and non-English reports were excluded. Two reviewers independently screened and extracted study, intervention, and neuroimaging details; heterogeneity precluded meta-analysis, so results were narratively synthesized by modality and linked to putative mechanisms and longitudinal effects. Results: Forty-seven studies met the criteria: 30 EEG, 12 fMRI, and 5 fNIRS. **Results**: Regarding EEG, massage commonly increased alpha across single sessions with reductions in beta/gamma, alongside pressure-dependent autonomic shifts; moderate pressure favored a parasympathetic/relaxation profile. Connectivity effects were state- and modality-specific (e.g., reduced inter-occipital alpha coherence after facial massage, preserved or reorganized coupling with hands-on vs. mechanical delivery). Frontal alpha asymmetry frequently shifted leftward (approach/positive affect). Pain cohorts showed decreased cortical entropy and a shift toward slower rhythms, which tracked analgesia. Somatotopy emerged during unilateral treatments (contralateral central beta suppression). Adjuncts (e.g., binaural beats) enhanced anti-fatigue indices. Longitudinally, repeated programs showed attenuation of acute EEG/cortisol responses yet improvements in stress and performance; in one program, BDNF increased across weeks. In preterm infants, twice-daily massage accelerated EEG maturation (higher alpha/beta, lower delta) in a dose-responsive fashion; the EEG background was more continuous. In fMRI studies, in-scanner touch and reflexology engaged the insula, anterior cingulate, striatum, and periaqueductal gray; somatotopic specificity was observed for mapped foot areas. Resting-state studies in chronic pain reported normalization of regional homogeneity and/or connectivity within default-mode and salience/interoceptive networks after multi-session tuina or osteopathic interventions, paralleling symptom improvement; some task-based effects persisted at delayed follow-up. fNIRS studies generally showed increased prefrontal oxygenation during/after massage; in motor-impaired cohorts, acupressure/massage enhanced lateralized sensorimotor activation, consistent with use-dependent plasticity. Some reports paired hemodynamic changes with oxytocin and autonomic markers. **Conclusions**: Across modalities, massage reliably modulates central activity acutely and shows convergent signals of neuroplastic adaptation with repeated dosing and in developmental windows. Evidence supports (i) rapid induction of relaxed/analgesic states (alpha increases, network rebalancing) and (ii) longer-horizon changes—network normalization in chronic pain, EEG maturation in preterm infants, and neurotrophic up-shifts—consistent with trait-level recalibration of stress, interoception, and pain circuits. These findings justify integrating massage into rehabilitation, pain management, mental health, and neonatal care and motivate larger, standardized, multimodal longitudinal trials to define dose–response relationships, durability, and mechanistic mediators (e.g., connectivity targets, neuropeptides).

## 1. Introduction

Massage therapy is most commonly defined as the manual manipulation of the body’s soft tissues delivered by a trained practitioner with the intention of supporting a client’s health and well-being; professional and academic sources converge on this core idea even though titles and scopes vary by jurisdiction and school [1]. Etymologically, the word “massage” is traced by historians either to an Arabic root for pressing/touching or to a Greek root for kneading. This ambiguity neatly mirrors the field’s diversity.

Modern Western lineages coalesced in the 19th century, notably “Swedish massage,” associated with Per Henrik Ling’s gymnastics movement and later codified with named strokes—effleurage, petrissage, friction, tapotement, and vibration—that remain the backbone of entry-level curricula and many credentialing exams. In the United States, massage featured in mainstream medical texts in the late 19th and early 20th centuries (for example, J. H. Kellogg’s The Art of Massage in 1895 [2]) before being reclassified in mid-century as a complementary or alternative treatment as biomedicine and pharmaceuticals ascended. Interest rebounded among consumers and clinicians in the late 20th and early 21st centuries, a trend documented in national surveys of “unconventional medicine.”

Massage delivers mechanical forces to skin, fascia, and muscle that cells convert into signals (mechanotransduction), shifting inflammatory pathways and pain processing; controlled animal and human work shows reduced immune-cell infiltration, edema, and pro-inflammatory signaling after damaging exercise when massage is applied, accelerating functional recovery and limiting secondary injury [3]. At the tissue level, massage behaves as a mechanotherapy that adapts skeletal muscle via effects on satellite-cell proliferation, immunoregulation, extracellular-matrix turnover, and intracellular protein and ribosome turnover, with evidence from cyclic compressive loading models that standardize force, frequency, and duration [4]. These mechanobiologic effects include dose-dependent modulation of macrophage phenotypes: appropriately dosed compressive loading favors increases in reparative M2 macrophages in healthy muscle and, when applied soon after injury, appears to attenuate neutrophil-driven secondary damage and reduce pro-inflammatory cytokines (e.g., TNF-α, IL-6), thereby creating a milieu conducive to repair and less nociceptor sensitization [3]. Beyond local tissue changes, massage consistently engages autonomic and endocrine axes when delivered with moderate pressure: studies report increased vagal activity, lower heart rate, and reduced cortisol, consistent with stress- and emotion regulation [5]. In preterm infants, moderate-pressure protocols increase vagal activity and gastric motility and are associated with higher insulin and IGF-1 levels, two pathways plausibly mediating improved nutrient absorption and weight gain [5]. Immunologically, moderate-pressure massage has been linked to enhanced natural killer (NK) cell numbers/activity in several groups; a proposed chain is pressure-receptor stimulation → vagal activation → cortisol reduction → preservation/augmentation of immune effector cells [5]. Cutaneous affective touch pathways likely contribute to unmyelinated C-tactile afferents and associated oxytocinergic circuits that can dampen HPA axis reactivity and support analgesic, anxiolytic, and anti-inflammatory effects observed with specific massage techniques [6]. Locally, massage increases skin and intramuscular temperature (via frictional heating and hyperemia). At the same time, evidence for large-artery muscle blood flow changes remains limited/inconclusive with classical techniques—pointing to possible microcirculatory or non-hemodynamic mechanisms for many benefits [7]. Neurologically, moderate pressure can reduce neuromuscular excitability (e.g., diminished stretch/H-reflex amplitudes), which may contribute to decreased muscle tension and pain [5].

Massage therapy has considerable potential, according to numerous studies. Reviews and meta-analytical studies have indicated that massage can be a valuable complementary intervention for various conditions. Massage has been shown to alleviate different types of pain, such as fibromyalgia [8], low back pain [9], neck pain [10], shoulder pain [11], migraine [12], post-surgery pain [13], and cancer pain [14]. Additionally, it has beneficial behavioral effects, alleviating symptoms of anxiety [15] and depression [16]. It improves motor function in Parkinson’s disease [17] and provides an additional intervention in the treatment of asthma [18] and hypertension [19].

As can be seen, many studies have examined the effectiveness of massage in neurological and psychiatric conditions. Often, the essence of these conditions is that they result from altered brain function, including brain activity. If massage beneficially reduces the symptoms of these conditions and improves patients’ well-being, it suggests that complex neural mechanisms likely underlie these effects at the brain level. To date, no review has collected and analyzed the effects of massage on brain activity measured using various neuroimaging techniques. These techniques include electroencephalography (EEG), functional magnetic resonance imaging (fMRI), and functional near-infrared spectroscopy (fNIRS). Such a mechanistic analysis could lead to a better understanding of the mechanisms of action of various massage techniques in the treatment of diseases. The research question is as follows: How does practitioner-applied manual massage modulate human brain activity measured with EEG, fMRI, and fNIRS, and what evidence supports neuroplastic (longer-term) adaptation with repeated massage exposure? To answer this question, we conducted a mechanistic scoping review of human trials that measured brain activity with EEG, fMRI, or fNIRS before/during/after massage or between massage and comparator conditions. Objectives were as follows:

1. Map acute neural effects of massage within each modality (e.g., EEG spectral power/connectivity, fMRI task/rest BOLD activation and functional connectivity, fNIRS hemodynamic responses);
2. Identify convergent mechanistic patterns across modalities and populations (healthy participants and clinical cohorts);
3. Evaluate signals consistent with neuroplasticity, including longitudinal changes after multi-session programs, developmental maturation effects, or persistent network alterations at follow-up;
4. Highlight methodological gaps (intervention heterogeneity, controls, outcome harmonization) and propose priorities for future multimodal, longitudinal studies.

This review focuses on practitioner-applied manual massage techniques (e.g., Swedish, Thai, shiatsu, tuina, reflexology, myofascial approaches) and excludes non-manual stimulation or studies limited to structural imaging.

## 2. Materials and Methods

This mechanistic review synthesizes evidence from clinical trials that assessed changes in human brain activity following massage interventions using electroencephalography (EEG), functional magnetic resonance imaging (fMRI), or functional near-infrared spectroscopy (fNIRS). Methods were informed by best practices for evidence syntheses and the PRISMA and PRISMA-ScR reporting principles where applicable. Because the aim was to map mechanistic signals across heterogeneous designs rather than to estimate pooled effects, the approach was scoping rather than fully systematic and did not adhere to all PRISMA items. No protocol was prospectively registered.

### 2.1. Data Sources and Search Strategy

An a priori search strategy was executed in July 2025 by two reviewers (J.C. and I.S.). We queried PubMed/MEDLINE, Cochrane Library, Google Scholar, and ResearchGate for records published from January 1990 through July 2025—search terms combined massage-related keywords with neuroimaging terms and their common variants. A representative PubMed string was (“massage” OR “reflexology” OR “tui na” OR “tuina” OR “shiatsu” OR “Thai massage” OR “myofascial release” OR “manual therapy”) AND (“EEG” OR “electroencephalography” OR “electroencephalogram” OR “functional MRI” OR fMRI OR “functional magnetic resonance imaging” OR fNIRS OR “functional near infrared spectroscopy”).

Search syntax was adapted for each database. We used PubMed’s “similar articles” and citation tracking features to enhance coverage and screened reference lists of eligible studies. Records were exported to a reference manager for de-duplication before screening.

### 2.2. Study Selection Criteria

Eligible studies were human clinical trials (randomized or non-randomized) published in English between January 1990 and July 2025 that met the following criteria:

1. Evaluated a massage intervention (e.g., Swedish, Thai, shiatsu, tui na/tuina, reflexology, myofascial release, or other practitioner-delivered manual massage);
2. Measured brain activity with EEG, fMRI, or fNIRS either pre/post-intervention or between groups.

We excluded reviews, editorials, non-English publications, animal studies, protocols without results, and studies limited to structural MRI or other modalities that do not index neural activity (e.g., anatomical MRI only). Trials using non-manual stimulation (e.g., TENS, vibration devices) without a massage component were also excluded.

### 2.3. Screening Process

**Figure 1:**
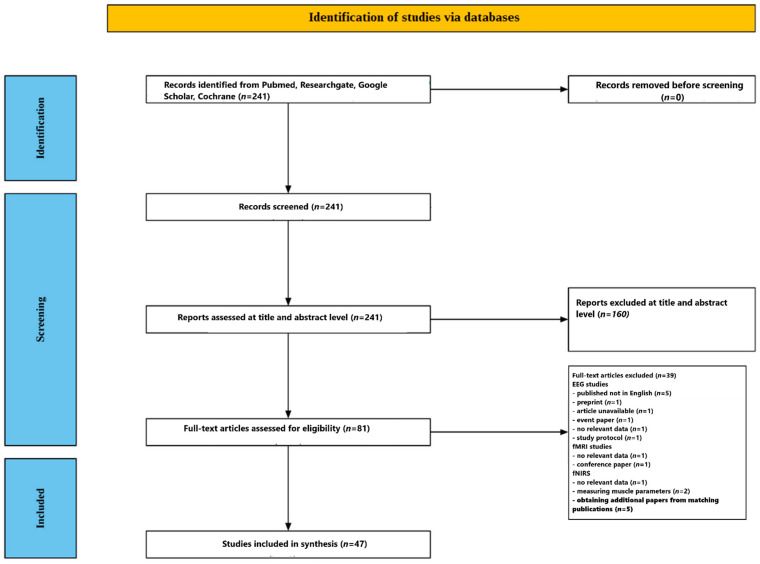
Flowchart depicting the different phases of the systematic review.

A multistage screening workflow was used to ensure transparent and reproducible study selection. Two reviewers (J.Ch. and D.K.) independently screened records and resolved disagreements by discussion; if consensus was not reached, a third senior author adjudicated. Inter-rater agreement was monitored during pilot screening to harmonize decisions. The study selection process is summarized in a PRISMA-style flow diagram in Figure 1.

#### 2.3.1. Title and Abstract Screening

Titles and abstracts were screened against the inclusion criteria, emphasizing (i) presence of a bona fide massage intervention and (ii) acquisition of EEG, fMRI, or fNIRS outcomes indexing neural activity.

#### 2.3.2. Full-Text Assessment

Records passing initial screening underwent full-text review to verify eligibility (study design, human participants, publication window, English language) and confirm that neuroimaging outcomes reflected brain activity (e.g., EEG spectral power/connectivity, fMRI BOLD activation/connectivity, fNIRS HbO/HbR changes). Reasons for exclusion at the full-text stage were recorded.

### 2.4. Data Extraction and Synthesis

Data extraction was performed independently by two reviewers using a standardized form. Extracted fields were cross-checked, and discrepancies were resolved by consensus (third-author adjudication when necessary). Using a standardized form, we extracted study design; sample size and demographics; clinical context (healthy vs. clinical populations); massage modality; body region(s); provider qualifications; session duration/frequency/dose; comparator conditions (e.g., sham touch, rest, usual care); neuroimaging modality and acquisition parameters (e.g., EEG montage and frequency bands, fMRI sequence and task/rest status, fNIRS optode configuration and wavelengths); preprocessing pipelines and motion/artifact handling; primary neurophysiological outcomes; timing of measurements; and statistical approaches. Given expected heterogeneity in interventions and analytic pipelines, we conducted a narrative synthesis, organizing findings by modality (EEG, fMRI, fNIRS), massage type, and population and noting convergence/divergence across methods.

### 2.5. Operational Definitions and Scope

For this review, “massage” denotes practitioner-delivered manual manipulation or sustained pressure of soft tissues with therapeutic intent. If delivered manually, reflexology and tui na/tuina were included as massage subtypes. Studies exclusively employing acupuncture/needling were outside the scope unless a manual massage component was the primary intervention.

### 2.6. Critical Appraisal and Risk of Bias

This review was designed as a mechanistic/scoping synthesis to map neurophysiological signals across heterogeneous massage protocols and neuroimaging pipelines rather than to estimate pooled treatment effects. Therefore, we did not conduct a formal risk-of-bias or study-quality rating (e.g., Cochrane RoB2/ROBINS-I), and we did not exclude studies based on methodological quality.

## 3. Results

Figure 1 provides a summary of the screening process. Of the 241 studies initially identified through database search, 160 were excluded based on title and abstract review. The remaining 81 articles were assessed by reading the full texts. At this stage, 39 articles were excluded. Regarding EEG studies, 10 preliminary matching studies were excluded: 5 because they were published in languages other than English, 1 article was a preprint, 1 article was unavailable, 1 found paper was an advertisement for an event, 1 article was a conference proceeding without relevant data, and 1 was a study protocol.

Regarding fMRI studies, 2 preliminary matching studies were excluded: 1 because it contained no relevant data and 1 because it was a conference paper. Regarding the fNRIS preliminary matching studies, 3 results were excluded: 1 because it was a study protocol, and 2 measured muscle parameters. At this stage, 47 studies were identified that fit the scope of the review. A search for similar and cited articles yielded an additional 5 articles. Ultimately, 47 articles were included in the review: 30 using EEG [20,21,22,23,24,25,26,27,28,29,30,31,32,33,34,35,36,37,38,39,40,41,42,43,44,45,46,47,48,49], 12 using fMRI [50,51,52,53,54,55,56,57,58,59,60,61], and 5 using fNIRS [62,63,64,65,66].

### 3.1. EEG Studies

**Figure 2:**
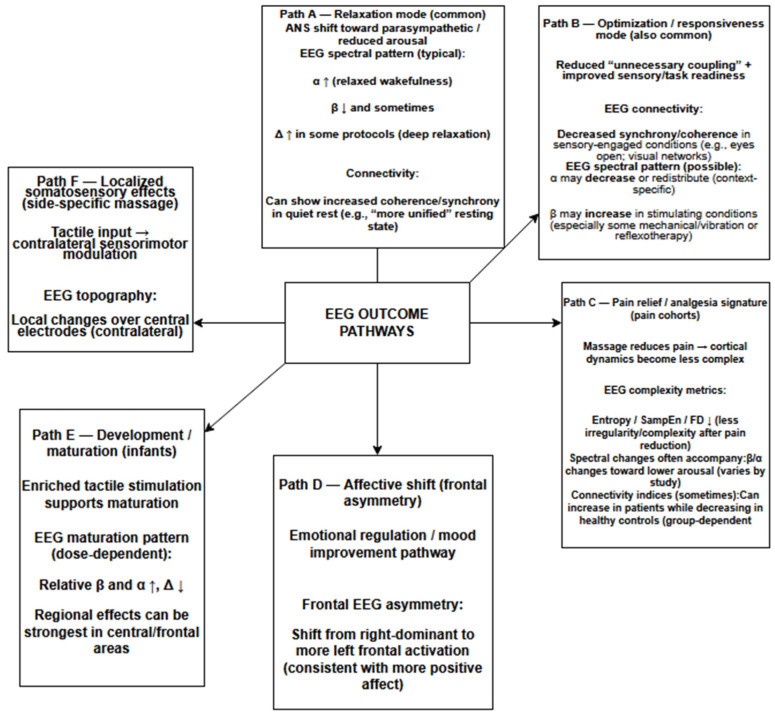
A graphical summary of the EEG results.

The included EEG studies are presented in Table 1. A graphical summary of the EEG results is provided in Figure 2.

#### 3.1.1. Participants’ Characteristics

Across the included studies, participants span the lifespan and a wide range of health states. At the earliest end, very preterm and very-low-birth-weight infants in NICUs received structured maternal or nurse-delivered massage protocols, with EEG or aEEG recorded at term-equivalent age [34,35,49]; another neonatal cohort comprised one-month-old infants of clinically depressed adolescent mothers, assessed for frontal EEG asymmetry during a brief massage session [48]. In childhood, typically developing preschoolers and school-age children underwent playful, CT-afferent–oriented back “story” massages with concurrent EEG and heart rate monitoring. Most adult samples were healthy young or middle-aged volunteers—often university students or community recruits—who received single-session cervical-collar, Swedish, facial/esthetic, manual lymph drainage, Shirodhara/Abhyanga, or programmatic chair/aromatherapy massages [20,22,23,24,27,28,29,30,31,32,33,43]; additional nonclinical cohorts included mothers of children with ADHD [29] and workers with occupational leg swelling in a workplace-relevant crossover design [47]. Older adults were represented by community-dwelling and long-term-care populations, including groups with and without nursing-care needs and a crossover comparing hand versus foot massage in the eighth decade of life [21,39]. Clinical pain populations included specific low back pain with disc herniation, lumbar disc herniation, scapulocostal syndrome, and mixed skeletal muscle pain, typically studied in single 15–30 min therapeutic sessions [36,37,44,46].

Samples were generally small and single-site (approximately *n* = 5–71), with a mixture of pre–post within-subject designs and several randomized or crossover comparisons (e.g., foam rolling vs. manual massage vs. autogenic training; massage chair ± binaural beats; traditional Thai massage vs. physical therapy; hand vs. foot massage; maternal–infant massage vs. standard care) [33,34,39,41,44,49]. Sex composition varied: some cohorts were female-only (stressed female students, female undergraduates, and adult women workers; Refs. [25,47]), one reflexotherapy study enrolled only right-handed males [31], and multiple studies used mixed-sex samples, including a precisely balanced 20 males/20 females in the cervical-collar trial [20]; handedness was specified as right-handed in several EEG protocols [22,31]. Recruitment frequently targeted stressed cohorts using the Stress Response Inventory [23,24,25,26,27,28,29,30,31,32,33,34,35] or defined clinical diagnoses [36,37,44,46], while standard exclusions removed neurological/psychiatric illness, cardio-muscular disease, and medications likely to alter EEG signals [23,36,38,41]. Settings ranged from NICUs and hospital clinics to university labs, workplaces, and long-term-care facilities, reflecting both controlled experimental contexts and ecologically relevant environments.

#### 3.1.2. Types of Massage Interventions

Across the set, interventions span therapist-delivered manual techniques, self- or device-assisted methods, and hybrid protocols with sensory add-ons. Manual, therapist-delivered work includes cervical-collar massage combining general and point techniques for 20 min in a single session [20]; three 5 min within-subject conditions—oil massage, lavender aromatherapy (inhalation), and aroma-oil massage—with the oldest/nursing-care group skipping aroma-only [21]; unilateral moderate-pressure Swedish massage to the arm/forearm/hand/neck/face for 25 min per side (20 min arm + 5 min face) without oils, with brief rests between components and sides [22]; neck-focused manual lymph drainage (MLD) for 15 min versus quiet rest [23]; abdominal MLD (20 min) compared with conventional abdominal massage of equal duration [24]; a second 15 min neck MLD study targeting frontal alpha asymmetry [25]; hands-on acupoint work at left/right Jian-wai-yu and Zuo-fei-yu for 3 min each in a clockwise sequence with pre/during/post EEG [26]; a 20 min esthetic facial massage delivered across three recording days and contrasted with autogenic training and quiet rest [27]; a five-week chair massage program given twice weekly (session length not reported) versus guided muscle relaxation [28]; full-body aromatherapy massage (lavender + geranium in jojoba) for 40 min, twice weekly for four weeks, with immediate (~15 min) post-session EEG checks [29]; a controlled pressure comparison over back/shoulders/arms—10 min of moderate-pressure massage versus light-pressure massage versus vibratory stimulation while seated in a massage chair [30]; foot reflexotherapy stimulating defined reflex zones for ~5 min per foot (~10 min total) [31]; standardized Abhyanga whole-body oil massage followed by 45 min of Shirodhara with thermostatted herbal oil and peristaltic-pump flow control [32]; therapist-delivered manual massage (duration not reported) contrasted with foam rolling and autogenic training in a single standardized session [33]; maternal–infant massage of 15 min twice daily from 34 weeks PMA to term-equivalent age under randomization [34]; an NICU protocol three times daily for ten days (10 min moderate tactile stimulation prone + 5 min passive limb movements supine) [35]; a single 30 min professional therapeutic massage for regional musculoskeletal pain [36]; Chinese massage to the lower back/hips for 15 min in both lumbar disc patients and healthy controls [37]; a crossover in long-term-care elders comparing 15 min hand versus 15 min foot massage (gentle strokes, no reflexology pressure, fragrance-free oil; seated for hand, supine for foot) with a one-week washout [39]; playful children’s back-massage “stories” (rhythmic/slow/energetic/smooth sequences) designed to engage CT-afferents (duration not reported) [42]; a 20 min professional singing-bowl “sound massage” bracketed by resting EEG blocks [43]; 30 min of traditional Thai massage (deep thumb pressure along meridian lines plus passive stretching) versus physical therapy (ultrasound + hot packs) for scapulocostal syndrome under randomization [44]; a single 25 min therapist massage for specific low back pain [46]; and a randomized crossover of 15 min hand–leg massage versus 15 min machine massage for occupational leg swelling using fragrance-free oil, with a two-week washout [47].

Device-assisted and self-myofascial approaches include a mechanical version of the same 3 min per acupoint protocol used in the hands-on study, with identical sequencing and pre/during/post EEG [26]; foam rolling as self-myofascial release in a single standardized session (duration not reported) contrasted with manual massage and autogenic training [33]; a portable kneading-node shoulder massager used for 30 min in healthy adults [38]; an impedance-controlled dual-arm robotic system delivering pressing, rubbing, and stroking in a 10 min session with baseline/during/post EEG [40]; a 20 min full-body massage-chair routine tested in a crossover among rest-only, massage-only, and massage plus binaural beats (piano/nature sounds with 10→7→4→10 Hz shifts every 5 min) [41]; a single-channel frontal EEG headband that triggers temple vibration motors when beta activity exceeds set thresholds during cognitive tasks (session duration not specified) [45]; and the machine-massage leg condition paired with the hand-massage comparator in the occupational-swelling crossover [47].

#### 3.1.3. Types of EEG Measurements

Acquisition ranged from research-grade, multi-channel systems to consumer headsets. A 21-channel 10–20 montage captured resting eyes-closed (3 min) and eyes-open (3 min) activity for cervical-collar massage with cross-correlation synchronicity analysis [20]. Manual lymph drainage (MLD) studies used 18 scalp sites with absolute/relative power [23] and a frontal 6-channel array (Fp1–Fp2, F3–F4, F7–F8) for alpha-asymmetry indices over 5 min eyes-closed epochs [25]. Several works used 32-channel systems with coherence (e.g., hands-on vs. mechanical acupoint massage [26]) or 64-channel EEG for singing-bowl massage with full-band spectral time series [43]. Targeted montages included Fz, O1, and O2 for facial massage [27] and Fp1, Fp2, O1, and O2 for aromatherapy massage [29]. Pain/robotics studies frequently employed Emotiv EPOC/EPOC+ (14-ch, 128 Hz) with preprocessing (ICA, high-pass filtering, artifact rejection) [36,37,40,46]; a small anti-aging study used NeuroSky MindWave single-sensor EEG [38]. Infant work used both conventional spectral EEG (eight derivations during active sleep) [35] and aEEG with global relative power (GRP) and Burdjalov-style maturation scoring at term-equivalent age [34,49]. A workplace swelling trial and an elder hand/foot comparison coupled scalp EEG with eLORETA source estimates (e.g., left insula after hand massage; bilateral posterior cingulate after foot massage; left anterior cingulate after hand–leg massage) [39,47]. Task paradigms extended beyond rest: during-massage recordings [22,26,40]; motor imagery/execution blocks (quiet rest, MI-left/right, ME left/right) [36]; and prefrontal six-electrode fatigue indexing around chair-massage/binaural-beat sessions [41].

Spectral bands were reported explicitly and sometimes with sub-bands. Typical adult ranges were δ 0.5–4 Hz, θ 4–8 Hz, α 8–13 Hz, β 13–30 Hz [22,26]; variants included δ 1–4 Hz [23]; split-beta (β-low 13–20, β-high 21–30 Hz) and classic α 8–12 Hz in DFT/Hanning analyses [28]; α1 8–10, α2 10.5–12, β1 12.5–15, β2 15.5–25, γ 25.5–45 Hz for singing-bowl massage [43]; and extended high-frequency bins for reflexotherapy (β 13–30, γ1 31–40, γ2 41–50, γ3 60–80 Hz) with δ 1–3, θ 4–7, α 8–12 Hz [31]. Aromatherapy-chair fatigue metrics used θ 3–9, α 9–13, β 13–30 Hz with θ/β and (θ + α)/β ratios [41]. Infant bands were shifted lower: δ 0.5–2, θ 2–6, α 6–13, β 13–30 Hz for GRP at TEA [34] and δ 0.5–4, θ 4.5–7.5, α 8–13, β 13.5–30 Hz for NICU FFT analyses [35]. Study-specific foci included regional effects at C4 (right central) for contralateral β suppression during left-arm Swedish massage [22] and frontal pairs for asymmetry with positive scores indicating greater left activation [25]; trend also inspected in [26,28,30].

Signal processing and derived metrics were diverse but precise. Power was computed as absolute and/or relative values with FFT (often Hanning-windowed DFT [27,28,43]), sometimes as spectral time series [43]. Connectivity was assessed via cross-correlation synchronicity (regional pairs [20]), classical coherence (showing alpha/beta reductions under mechanical massage [26]), and alpha inter-occipital coherence (O1–O2) decreases after facial massage [27]. Hemispheric alpha asymmetry quantified affective shifts in frontal leads [25], with complementary observations in [28,30]. Several pain and rehabilitation studies emphasized complexity/entropy: ApEn, SampEn, Permutation Entropy, Wavelet Entropy, Fuzzy/Intrinsic Fuzzy Entropy, and HHT marginal spectrum entropy, often complemented by normalized band energy, wavelet packet band extraction, and a permutation disalignment index (PDI) for coupling [36,37,46]. Feature-based classification (e.g., CSP, SVM/logistic regression, CNN, BiLSTM with attention) evaluated the separability of MI/ME states and pre-/post-massage [48]. Source space mapping used eLORETA to localize alpha generators (insula, ACC, PCC) after specific body-site interventions [39,47]. Across designs, preprocessing explicitly addressed artifacts—ICA, MEMD + CCA for EMG, and wavelet-ICA for EOG in MI/ME protocols [36]—with standardized eyes-closed baselines (commonly 3–5 min) and pre/during/post blocks enabling within-subject contrasts.

#### 3.1.4. Massage Parameters (Site, Duration, Frequency, and Delivery Mode)

Massage sites were variable but fell into common anatomical categories: neck/cervical (e.g., cervical-collar massage; neck MLD) [20,23,25], upper limb/face [22,27], back/shoulder/acupoints [26,30,38,41], abdomen [24], lower back/hips [37,44,46], hand/foot/lower limb [31,39,47], and whole-body or multi-region protocols (often in aromatherapy or intensive infant protocols) [29,34,35,48,49]. Several interventions were explicitly targeted (acupoints, reflex zones, meridians) [26,31,44], while others used region-based therapeutic protocols (e.g., MLD, Swedish massage).

Session duration in adult studies clustered strongly within a short acute window, most commonly 10–30 min, with additional short exposures (e.g., 5 min) [21] and longer sessions up to 40–45 min in aromatherapy massage and Shirodhara [29,32]. Where frequency was reported, adult programs commonly used two sessions/week over 4–5 weeks [28,29]. In contrast, infant/preterm studies used substantially higher cumulative exposure (e.g., multiple sessions per day over days to weeks or twice daily until term-equivalent age) [34,35,49].

Delivery mode varied across manual therapist-administered massage [20,22,27,30,33,39,44], specialized manual techniques (MLD) [23,24,25], combined modalities (aromatherapy, binaural beats/sound, singing bowl) [29,41,43], and device-based formats including mechanical massage chairs and robotic massage [26,38,40,41]. Several studies directly contrasted manual versus mechanical delivery [26,47] or compared massage to non-massage relaxation controls [23,27,28,33,41,44].

#### 3.1.5. Pressure/Intensity Reporting and Evidence of Intensity-Dependence

Across the EEG literature, pressure intensity was rarely quantified in physical units (e.g., Newtons), and most studies relied on qualitative descriptors or technique-defined expectations of force. As a result, pressure was most often operationalized implicitly—through the massage modality (e.g., MLD vs. Thai massage), the delivery agent (therapist vs. device/robot), or the stated intention of the intervention (relaxation vs. stimulation vs. analgesia). Despite this limitation, the studies collectively provide convergent evidence that pressure/intensity is a determinant of EEG directionality and that even when self-reported relaxation improves, the underlying neurophysiological response can diverge depending on tactile intensity and mechanical characteristics.

Qualitative pressure descriptions were common, with several studies explicitly stating “light,” “moderate,” or “deep” pressure, or describing techniques that imply particular force ranges. For example, unilateral Swedish massage in healthy adults was delivered with “moderate pressure” and without oils to limit systemic confounds [22]. In contrast, traditional Thai massage for scapulocostal syndrome used deep thumb pressure along meridian-related lines followed by passive stretching, indicating a comparatively higher-intensity mechanical input [44]. Manual lymph drainage (MLD) interventions, by design, employ lighter, rhythmical skin-stretch techniques; while the summaries do not provide force values, MLD is characterized as a “light tactile intervention” and was treated as such in the stressed participant cohorts [23,24,25]. Device-based studies similarly used modality-dependent intensity settings but typically did not report calibrated force; instead, they emphasized device type (mechanical chair, shoulder massager, robot) and standardized program structure (e.g., pressing/rubbing/stroking or acupoint sequences) as the operative intensity proxy [26,38,40,41].

Within this reporting landscape, the most unmistakable evidence of intensity-dependence comes from the controlled trial directly comparing moderate-pressure massage, light-pressure massage, and vibratory stimulation delivered for 10 min to the back/shoulders/arms while seated [30]. This study provides a rare, explicit manipulation of tactile intensity within a shared experimental framework and demonstrates that intensity can produce opposing physiological profiles. Moderate-pressure massage produced the strongest objective relaxation signature, including decreased heart rate and EEG changes described as increased delta activity accompanied by decreased alpha and beta activity, alongside a shift toward greater left-frontal EEG asymmetry (interpreted as more positive affect) [30]. In contrast, light-pressure massage elicited a pattern consistent with heightened arousal, including decreased delta power with increases in theta and beta activity and elevated heart rate, even though participants still reported reductions in anxiety and stress (albeit less pronounced than in the moderate-pressure condition) [30]. Vibratory stimulation produced mixed but generally arousal-linked physiology (increases in theta, alpha, and beta, and increased heart rate) despite subjective anxiety reductions similar to the other groups [30]. Together, these results indicate that “massage” cannot be treated as a uniform exposure in EEG studies: intensity can flip the direction of band-limited changes, and subjective relaxation does not guarantee a relaxation-consistent electrophysiological state. Importantly for cross-sectional synthesis, this study also suggests that pressure category (light vs. moderate) is not merely a nuisance parameter but a primary mechanistic variable.

Evidence for intensity-dependence also emerges indirectly when comparing interventions that differ systematically in their typical tactile forces and sensory profiles. MLD studies in stressed adults consistently reported EEG changes interpreted as relaxation-related, including increases in alpha (and in some cases delta) after MLD sessions [23,24,25]. In the neck MLD randomized design, a 15 min session produced significant increases in absolute and relative alpha and delta compared with quiet resting controls [23]. In abdominal applications, both abdominal MLD and conventional abdominal massage increased alpha and reduced higher-frequency power, but MLD produced a greater increase in alpha power and a more pronounced reduction in relative gamma than standard abdominal massage, consistent with a more substantial calming effect under otherwise matched session duration (20 min) and participant stress status [24]. Frontal alpha asymmetry also shifted toward greater left-sided activation after neck MLD in stressed female participants, significant for the F7–F8 electrode pair, suggesting an intensity/technique capable of altering affect-relevant cortical balance even when tactile input is relatively light [25]. Although these MLD studies did not quantify applied force, their convergence suggests that low-intensity, parasympathetic-oriented tactile techniques can reliably modulate EEG markers of relaxation and affective state.

Conversely, higher-intensity or mechanically complex interventions showed profiles that were not uniformly “sedating” and sometimes resembled stimulation or reactivity. In Thai massage for pain patients, a single 30 min session produced an EEG profile described as increased delta with decreased theta, alpha, and beta, and these changes correlated with reductions in anxiety and pain [44]. This pattern indicates that deep pressure in a clinical pain context can produce robust shifts toward slow-wave dominance and reduced fast activity—consistent with a strong relaxation/analgesia response rather than activation. However, stimulation-like EEG effects appear in other modalities that likely differ in contact dynamics and afferent recruitment. Foot reflexotherapy, delivered as 5 min per foot targeting reflex zones, increased beta and gamma activity (especially left frontal) while decreasing delta, interpreted as heightened cognitive engagement rather than sedation [31]. This suggests that “intensity” is not only about force magnitude but also about the patterning of stimulation (localized, sometimes noxious/pressing stimulation in reflex zones vs. broad soothing strokes) and the intended effect (activation vs. relaxation), producing distinct EEG signatures.

A further proxy for intensity-related differences is the comparison of manual versus mechanical delivery. In a study applying massage to acupoints, hands-on massage was associated with increases in slower rhythms (delta/theta) more consistent with relaxation, whereas mechanical massage more often decreased delta/theta and increased beta, with larger coherence disruptions (notably reduced alpha and beta coherence) in the mechanical group [26]. Similarly, in occupational leg swelling, both hand and machine massage reduced leg volume. Still, only hand massage increased alpha activity in the left anterior cingulate cortex (source-localized), and hand massage produced higher subjective pleasantness/relaxation scores [47]. These findings do not isolate “pressure” per se. Still, they indicate that device delivery—likely differing in micro-kinematics, force variability, and sensory predictability—can shift EEG outcomes in directions not identical to therapist-delivered tactile input, reinforcing the idea that intensity should be conceptualized as a multicomponent construct (force magnitude, temporal dynamics, spatial distribution, and sensory controllability).

Finally, even when studies used similar qualitative labels (e.g., “moderate pressure”), intensity effects appeared regionally specific. Unilateral Swedish massage is described as moderate pressure, reduced slow-wave power (delta/theta), and increased alpha across bilateral frontal/fronto-central regions while also decreasing beta in a localized contralateral central site during left-arm stimulation, consistent with somatosensory inhibition that preceded more generalized relaxation effects [22]. Such findings suggest that intensity interacts with somatotopic processing and may produce both local and global EEG signatures depending on where force is applied and how stimulation propagates across sensorimotor networks.

#### 3.1.6. EEG Spectral Power in the Efferent State and During Massage

Across the included studies, spectral power was most commonly quantified in canonical frequency bands—delta (approximately 0.5–4 Hz), theta (4–8 Hz), alpha (8–13 Hz), and beta (13–30 Hz)—with several studies additionally examining gamma activity (typically >30 Hz) or subdividing alpha/beta into narrower sub-bands. EEG was most frequently collected in resting-state (often eyes closed), either immediately pre- or post-intervention or with additional recording during massage; a smaller subset used repeated-session designs (weeks) or neonatal longitudinal follow-up. Although spectral outcomes were not uniform in direction across all massage methods, populations, and recording conditions, multiple studies reported statistically detectable band-limited changes occurring either during stimulation or in the acute post-massage period.

In resting-state paradigms with adult participants, multiple interventions produced measurable shifts in slow-wave and alpha/beta activity. In the cervical-collar study (20 min combined general + point massage), effects were described primarily in terms of cross-correlation synchronicity. Still, the protocol included standardized resting recordings with both eyes closed and eyes open (3 min each) before and after the massage, providing evidence that even brief post-massage resting intervals are sufficient to capture altered electrophysiological states in this site-specific intervention [20]. Several studies that explicitly quantified spectral power reported acute post-massage changes consistent with altered arousal level. In a neck manual lymph drainage (MLD) trial in psychologically stressed students (15 min intervention versus quiet rest control), the experimental group showed significant increases in absolute and relative alpha as well as increases in delta after treatment compared with baseline and control, with recordings taken before and after the session across multiple scalp sites [23]. A related neck MLD asymmetry study used eyes-closed frontal EEG and found an alpha-band asymmetry shift toward greater left-sided activation (significant at F7–F8), indicating that post-session spectral measures in the alpha range can change detectably after a single 15 min treatment even when recorded only from frontal channels [25]. In abdominal protocols, both a 20 min abdominal MLD session and a conventional abdominal massage session increased alpha activity. They reduced faster activity, but the MLD group exhibited a larger increase in absolute/relative alpha and a more pronounced reduction in relative gamma, indicating that spectral effects can differentiate between two abdominal tactile techniques delivered for the same duration in stressed individuals [24].

Single-session interventions in clinical pain cohorts also yielded robust spectral changes. In a randomized trial of scapulocostal syndrome, traditional Thai massage (30 min) produced a post-treatment pattern of increased delta and decreased theta, alpha, and beta, whereas the physical therapy comparator showed minimal spectral change aside from slight increases in alpha and beta; spectral shifts in the Thai massage group were also associated with larger reductions in pain and anxiety (VAS/STAI), but the electrophysiological result itself was expressed as band-specific increases/decreases following the intervention [44]. In lumbar disc herniation (LDH), a 15 min Chinese massage focused on the lower back and hips altered rhythm energy profiles alongside entropy changes: LDH patients showed a post-treatment increase in delta energy (with prominent left-hemisphere distribution) and significant decreases in theta and alpha energy, while healthy controls showed a different pattern (including increased delta entropy but reduced alpha/beta complexity), demonstrating that baseline clinical state modifies how spectral/energy measures shift after the same duration and region of massage [37]. A separate pain-related EEG motor paradigm study (30 min professional massage in individuals with skeletal muscle pain) emphasized entropy reductions in the alpha band across resting and motor imagery/execution states. Still, it also segmented data into delta/theta/alpha/beta rhythms for subsequent analyses, indicating that massage-related electrophysiological changes can be observed not only at rest but also across multiple task states within the same participant [36].

Several studies in healthy adults reported spectral changes either during massage, after massage, or both. In unilateral Swedish massage, EEG was recorded at baseline (eyes-closed resting) and during massage of one side of the arm/face (25 min per side). Compared with baseline, massage was associated with reduced delta and theta absolute power across frontal/fronto-central/central regions and increased alpha power in simulations; additionally, a localized reduction in beta power occurred over the right central area (C4) during left-arm massage, demonstrating that spectral power effects can occur during ongoing stimulation and may include both widespread and region-specific band changes [22]. In a singing-bowl massage study with a 64-channel EEG recorded across baseline, massage, and a post-massage integration phase, spectral power time-series analyses across multiple sub-bands showed a global decrease in activity during the massage compared with initial rest, with particularly notable reductions in higher-frequency beta2 and gamma bands; after the massage, overall EEG power declined further relative to baseline, indicating sustained decreases in spectral power beyond the stimulation period in this modality [43]. In a small study of machine-assisted shoulder massage (30 min) using a single-channel consumer EEG device, alpha amplitude increased significantly post-massage. At the same time, delta/theta decreased slightly, and beta/gamma increased slightly without statistical significance, suggesting that even simplified acquisition approaches can detect post-session alpha enhancement in some protocols. However, this evidence is limited by sample size and device constraints [38].

Studies comparing manual and mechanical delivery or multicomponent stimulation showed that spectral power changes depend on delivery characteristics and co-interventions. In a hands-on versus mechanical acupoint massage study (3 min per acupoint, four acupoints, recorded before/during/after), hands-on massage generally increased slower rhythms (delta, theta) across stages, whereas mechanical massage more often decreased slow rhythms and was associated with relatively greater beta increases during treatment phases; alpha tended to decline during sessions, particularly in the mechanical group, indicating distinct band-specific trajectories across massage stages in the two delivery modes [26]. A dual-arm robotic massage pilot (10 min) recorded baseline, during, and post-massage EEG and reported post-treatment increases in delta power and decreases in alpha power, particularly at frontal sites (F3/F4), indicating that automated, controlled delivery can produce detectable spectral shifts in small samples using standard band definitions [40]. In a crossover fatigue study using a mechanical massage chair, massage alone did not significantly change EEG fatigue indices derived from theta/alpha/beta ratios, whereas mechanical massage combined with binaural beats produced significant reductions in theta/beta and (theta + alpha)/beta indices across prefrontal electrodes; this result implies that band power relationships (rather than single-band changes) can be selectively sensitive to multimodal stimulation conditions and may not shift under massage-alone conditions depending on the endpoint and montage [41]. Similarly, in an oil massage/aromatherapy comparison study with 5 min interventions in adults and older adults, band changes were summarized as pre/post ratios for alpha, beta, and theta and varied by age group and condition, with increases in alpha trending particularly during aromatherapy conditions in groups without nursing care and theta changes being more prominent in the long-term care group, indicating that short exposures can still generate detectable but heterogeneous band ratio shifts across populations and modalities [21].

Longer intervention programs revealed differences between immediate post-session spectral effects and longer-term baseline patterns. In a five-week workplace chair massage program (twice weekly), both massage and relaxation control groups showed increased frontal delta after sessions. Still, only the massage group showed decreases in frontal alpha and beta power alongside improved speed and accuracy on math computation, indicating that repeated exposure can produce consistent acute spectral changes linked to cognitive measures, at least at the endpoints sampled (first and last day, pre/post) [28]. In a four-week aromatherapy massage trial (40 min, twice weekly), alpha activity increased. Delta activity decreased when measured shortly after a session early in the program (e.g., post first treatment), but the magnitude of the acute EEG alteration weakened by the final (eighth) session; importantly, basal EEG measured before sessions did not differ between the first and last session, and there was no significant long-term baseline EEG change after four weeks, suggesting that repeated aromatherapy massage can yield robust acute band shifts without necessarily producing durable resting-state baseline alterations in the spectral measures captured [29]. In Shirodhara (preceded by full-body Abhyanga and followed by a 45 min forehead oil drip), EEG analysis reported increased alpha post-procedure, again reflecting acute post-session spectral modulation under a standardized delivery apparatus, but without providing evidence of long-term baseline change in the summarized outcome [32].

Infant and neonatal studies measured spectral power differently (relative power, maturation indices, aEEG continuity) but still documented massage-related modulation of band activity and trajectories over time. In very preterm infants receiving maternal massage from 34 weeks gestational age to term-equivalent age, the primary endpoint (global relative beta power) did not differ between massage and standard care; however, secondary analyses found higher alpha relative power in central regions, and the number of sessions correlated with more mature profiles (higher relative power in beta/alpha/theta and lower delta), indicating a dose-linked relationship between cumulative tactile exposure and spectral maturation markers [34]. In a separate preterm cohort receiving massage three times daily for ten days, longitudinal EEG comparisons showed that control infants exhibited significant decreases in global delta and alpha power over time (consistent with expected developmental trajectories), whereas massaged infants showed no significant worldwide decline and displayed region-specific increases in central delta/theta power with decreases in temporal delta/alpha; between-group analyses demonstrated time-by-group interactions for global delta and for central delta and beta power, indicating that massage can alter the direction or magnitude of spectral change across early development windows [35]. A related VLBW infant study using amplitude-integrated EEG found a tendency toward more continuous background patterns and fewer delayed maturation scores in the massage group at 34 weeks PMA, supporting the feasibility of capturing maturation-related electrophysiological differences using prolonged bedside recordings rather than short resting-state segments [49]. In infants of depressed mothers, EEG was analyzed in the 3–13 Hz range and emphasized frontal asymmetry rather than full-band spectra, but the protocol recorded baseline, during, and post-massage periods and demonstrated measurable shifts in frontal activity balance during tactile stimulation, consistent with the broader observation that electrophysiological changes can occur rapidly during the intervention period itself [48].

Finally, several studies localized spectral power changes to specific regions or used source estimation rather than scalp-wide summaries, expanding how band power effects were expressed. In elderly individuals under long-term care, hand and foot massage both increased alpha-band activity. Still, in different cortical sources, hand massage increased alpha in the left insular cortex, while foot massage increased alpha in the bilateral posterior cingulate cortex, with resting-state EEG recorded before and after 15 min sessions and analyzed via eLORETA [39]. In occupational leg swelling, hand massage increased alpha activity in the left anterior cingulate cortex compared with machine massage despite similar peripheral swelling reduction, indicating that spectral power effects can be present at the cortical source level even when peripheral physiological outcomes are comparable across delivery modes [47]. In a facial massage study with repeated recording across days and limited electrode locations, alpha power showed a consistent post-session decrease (alpha attenuation) with slight theta increases and beta decreases, and alpha coherence between occipital electrodes decreased slightly after massage, demonstrating that spectral power and inter-hemispheric measures can shift in tandem and that directionality (alpha increase vs. decrease) may depend on the specific massage type, montage, and experimental setting (including repeated habituation procedures) [27].

#### 3.1.7. Functional Connectivity, Coherence, and Context Dependence

A subset of the included EEG studies moved beyond band-limited power to quantify functional interactions between channels or regions, most commonly using (i) phase-based measures, (ii) spectral coherence in canonical bands, or (iii) connectivity-sensitive complexity metrics such as the permutation disalignment index (PDI). Across these studies, connectivity outcomes were consistently context dependent, varying as a function of recording condition (eyes open vs. eyes closed; resting vs. task), massage stage (pre, during, post), delivery mode (hands-on vs. mechanical), and participant group (healthy vs. pain/clinical cohorts). Notably, the directionality of connectivity change was not uniform: some interventions increased within-network synchrony consistent with a more unified resting state, while others reduced synchrony—particularly in conditions requiring sensory processing or where stimulation was more mechanical and less socially/affectively modulated.

##### Cross-Correlation Synchronicity Reveals State-Dependent Network Reconfiguration (Eyes Closed vs. Eyes Open)

The most explicit demonstration of context dependence at the network-interaction level came from the cervical-collar massage study [20], which explicitly compared pre–post changes under two standardized resting conditions. Using a 21-channel EEG with a 10–20 montage, the authors applied cross-correlation analysis to quantify synchronicity between pairs of cortical regions. They found that massage altered inter-regional coupling in opposite directions depending on whether participants rested with eyes closed or eyes open. During eyes-closed rest, post-massage coupling increased between frontal and central regions in the left hemisphere, indicating strengthened inter-regional synchronicity consistent with a more unified and passive cortical state [20]. In contrast, during eyes-open rest, post-massage coupling decreased between central and occipital regions in the right hemisphere, a pattern interpreted as reduced unnecessary coupling and a functional shift toward greater sensory responsiveness under visual engagement [20]. These results are directly relevant for cross-study comparability because they demonstrate that even with the same participants, the same massage protocol, and the same EEG montage, connectivity effects can reverse depending solely on resting context (eyes open vs. eyes closed). Accordingly, connectivity findings across studies cannot be meaningfully compared unless the recording state and sensory context are aligned.

##### Coherence Outcomes Differ Markedly by Delivery Mode and Evolve Across Massage Stages

A second primary source of context dependence was massage delivery mode, most prominently in the study comparing hands-on massage by a therapist versus mechanical massage using a device applied to a sequence of four acupoints (3 min per acupoint) with an EEG recorded before, during, and after stimulation using a 32-channel system [26]. Here, connectivity was operationalized via EEG coherence, computed across rhythms (δ, θ, α, β). The study reported that hands-on massage maintained relatively stable coherence patterns across stages, suggesting preserved functional coordination during tactile manipulation [26]. In contrast, the mechanical massage condition produced greater coherence variability and more pronounced coherence reductions, particularly in alpha and beta bands, interpreted as a disruption or destabilization of functional network organization during mechanically delivered stimulation [26]. Stage-dependent effects were also evident: coherence alterations were not confined to the post period but emerged dynamically across the “during” phase, indicating that coherence measures can index the immediate network response to ongoing tactile input rather than only a post-treatment aftereffect [26]. These data complement the cross-correlation findings in [20] by showing that functional coupling depends not only on the participant’s sensory state but also on how stimulation is delivered (human touch vs. device-driven patterns), with mechanical delivery producing larger coherence perturbations in higher-frequency rhythms associated with attention and active processing.

##### Inter-Hemispheric Coherence Changes Accompany Relaxation-Related Spectral Shifts in Facial Massage

Connectivity changes were also reported in a more spatially limited montage in the facial massage experiment [27], which recorded spontaneous EEG from Fz, O1, and O2 and computed both spectral power and coherence. While the primary spectral result was a consistent post-massage alpha attenuation, coherence analysis showed that alpha coherence between O1 and O2 decreased slightly after facial massage [27]. This reduction in occipital inter-hemispheric coherence is consistent with decreased regional synchrony during a relaxed, low-demand state. It further illustrates that relaxation-associated effects can manifest either as increases in coherence (as in the eyes-closed fronto-central coupling increase in [20]) or as decreases (as in occipital alpha coherence in [27]), depending on which regions are sampled and which functional processes dominate the recording context (visual system coupling vs. fronto-central resting integration).

##### Connectivity-Sensitive Complexity Metrics (PDI) Show Clinical-State-Dependent Coupling Changes After Massage

Two pain-focused studies extended connectivity assessment using entropy- and alignment-based metrics that capture inter-channel coordination rather than simple band power alone. In the skeletal muscle pain study using motor imagery (MI) and motion execution (ME) tasks [36], connectivity was quantified using the permutation disalignment index (PDI), and significant post-massage changes were reported, particularly in the beta rhythm [36]. Because recordings were acquired across multiple task states (rest, MI left/right bending, ME left/right bending), the observed PDI changes indicate that massage altered inter-channel coupling not only at rest but also during complex sensorimotor cognitive states. Thus, context dependence in this study is twofold: connectivity shifted as a function of massage and as a function of task state, implying that connectivity endpoints may be sensitive to whether the brain is engaged in imagery/execution versus passive rest.

In the lumbar disc herniation study [37], connectivity was also assessed via PDI in addition to multiple entropy measures and rhythm energy. Notably, the direction of coupling change differed by group: massage enhanced cortical connectivity (via PDI) in LDH patients but reduced connectivity in healthy controls [37]. This group divergence is critical for synthesis because it indicates that baseline clinical state can determine whether massage consolidates functional coupling (potentially reflecting analgesia-related stabilization) or reduces coupling (potentially reflecting relaxed disengagement) under similar recording and intervention timing. Together, refs. [36,37] show that connectivity effects are not merely noise around spectral changes; rather, they may reflect clinically meaningful shifts in how distributed regions coordinate during rest and action-related cognition.

##### Synchronization Metrics in Resting-State EEG Further Indicate Region-Specific Network Modulation

In addition to explicit coherence/correlation/PDI studies, one randomized trial comparing foam rolling and manual massage analyzed resting-state EEG in terms of alpha and beta synchronization, reporting increases in synchronization after both interventions with differing spatial distributions (foam rolling predominantly frontal; manual massage predominantly parietal) [33]. Although this study emphasized synchronization rather than pairwise coherence, it supports the broader pattern that massage can modulate the organization of resting-state rhythms in a regionally specific manner and that “relaxation-related” network effects may not be uniform across the scalp but depend on whether the intervention is actively self-administered (foam rolling) or passively received (manual massage) [33]. These results align with the delivery-mode dependence seen in [26], albeit using different operational metrics.

#### 3.1.8. Regional and Lateralized Effects (Somatosensory Mapping, Frontal Asymmetry)

Across the included EEG studies, several interventions produced topographically specific and/or hemispherically lateralized effects, indicating that massage not only shifts global arousal markers but can also modulate activity in circuits consistent with somatotopic organization, frontal affective asymmetry, and region-dependent sensitivity to tactile input. These regional and lateralized results were reported using different EEG endpoints—including band-limited power, asymmetry indices, cross-correlation/coherence patterns, and source-localized alpha activity—and they emerged in both healthy and clinical populations.

##### Somatosensory Mapping: Contralateral Sensorimotor Modulation During Unilateral Stimulation

The most direct evidence for somatotopic, lateralized modulation was provided by the unilateral Swedish massage study in healthy right-handed adults [22]. In that protocol, EEG was recorded during baseline eyes-closed rest and during massage applied unilaterally to the arm/forearm/hand (20 min) and face/neck (5 min), with each side treated separately. In addition to a generalized pattern of reduced slow-wave power (delta/theta) and increased alpha power across bilateral frontal and central regions, a localized lateralized effect was detected in the beta band: beta power decreased significantly over the right central scalp site (C4) during left-arm massage [22]. Because C4 overlies the right sensorimotor cortex, which processes somatosensory input from the left side of the body, this finding is consistent with a contralateral inhibitory modulation within the somatosensory system during ongoing tactile stimulation. The authors interpreted this pattern as evidence that unilateral massage may first exert a localized effect in the contralateral somatosensory cortex and then propagate more generalized relaxation effects bilaterally. This study, therefore, provides a mechanistic anchor for claims of region-specific neural modulation: EEG changes were not only global but were also spatially congruent with the stimulated body side, consistent with known cortical representations.

Complementary support for region-specific modulation is also present in studies where the massage site differed, but the EEG response was anatomically plausible. For example, cervical-collar massage altered inter-regional synchrony involving frontal and central regions (eyes closed) and central–occipital regions (eyes open) [20], which is consistent with neck/upper shoulder stimulation influencing fronto-central networks involved in sensorimotor integration and vigilance, with additional context-dependent effects on posterior sensory processing when eyes are open. Although [20] did not explicitly operationalize somatotopy, it reinforces the principle that massage-related EEG effects can be regionally patterned rather than diffuse.

##### Frontal EEG Asymmetry: Affect-Related Lateralization Shifts After Tactile Interventions

Several studies assessed frontal EEG asymmetry, a well-established electrophysiological marker linked to affective style and motivational valence, typically operationalized using alpha power asymmetry (given the inverse relationship between alpha power and cortical activation). In stressed individuals, a neck MLD intervention produced an asymmetry shift toward relatively greater left-frontal activation after treatment [25]. Specifically, the asymmetry index in the alpha band shifted from a pattern dominated by greater right-sided activation (associated with negative affect/stress) toward a more left-dominant pattern (associated with positive affect), with the strongest statistically significant change observed at the F7–F8 electrode pair [25]. Although Fp1–Fp2 and F3–F4 did not reach significance, both showed changes in the same direction, supporting a coherent shift toward left-lateralized frontal engagement following a short (15 min) tactile intervention.

A similar affect-relevant lateralization effect was reported in the intensity manipulation study comparing moderate-pressure massage, light-pressure massage, and vibration [30]. Moderate-pressure massage was associated with a shift toward greater left-frontal asymmetry, alongside decreased heart rate and EEG patterns interpreted as relaxation physiology. This finding is notable because it suggests that frontal asymmetry is not only sensitive to technique type (e.g., MLD) but also to pressure intensity. That intensity may determine whether tactile stimulation engages affective frontal circuits in a direction consistent with a positive emotional state.

Frontal asymmetry effects were also documented in early development. In infants of depressed mothers, massage therapy reduced the characteristic right-frontal EEG asymmetry associated with depression-like affective profiles [48]. During the massage and immediately after the session, the right-dominant frontal pattern diminished, indicating a shift toward more balanced frontal activity. Importantly, this effect was specific to frontal sites (F3/F4) and was not observed in parietal regions (P3/P4), supporting anatomical specificity and reinforcing that tactile stimulation can acutely influence frontal systems implicated in emotional regulation even in very early life. While infant EEG frequency ranges differ from adult band conventions, the directionality and localization are consistent with the adult asymmetry literature: massage shifted frontal balance away from a pattern associated with negative affect risk.

##### Regional Specificity from Source-Localized Alpha Activity: Insula, ACC, and PCC

Several studies used source imaging approaches (eLORETA) to identify cortical generators of band-limited changes, providing a more direct link between scalp-recorded EEG effects and regional brain activity. In elderly individuals under long-term care, a crossover comparison of hand versus foot massage found that both interventions increased subjective pleasantness/relaxation. Still, they differed in regional alpha generators: hand massage increased alpha activity in the left insular cortex, whereas foot massage increased alpha in the posterior cingulate cortex (PCC) bilaterally [39]. These findings demonstrate that regional specificity can emerge even when self-reported outcomes appear similar, and they suggest that different body sites may preferentially engage distinct cortical hubs (insula for interoceptive/emotional awareness; PCC for self-referential processing).

A related dissociation between manual and machine delivery was reported in the occupational leg swelling study [47]. Both hand massage and machine massage reduced leg volume. Still, only hand massage increased alpha activity in the left anterior cingulate cortex (ACC), and it produced higher ratings of pleasantness, relaxation, and refreshment [47]. This suggests a lateralized, region-specific cortical signature associated with human-delivered touch that is not reproduced by machine-based stimulation, even when peripheral physiological benefits are comparable. The ACC is frequently implicated in affective regulation and evaluation of salient bodily states. Although these source-localized studies report alpha changes rather than broad spectral shifts, they are central for interpreting regional and lateralized effects: they demonstrate that massage-related EEG modulation is not restricted to scalp-wide power averages but can be mapped to specific cortical regions with known roles in affective and self-related processing.

##### Developmental Regional Effects: Central Lead Sensitivity and Maturation-Linked Topography in Preterm Infants

Regional effects were also evident in neonatal and preterm cohorts, where the primary interpretive framework is maturation rather than adult arousal. In very preterm infants receiving maternal massage from 34 weeks of gestational age to term-equivalent age, the primary outcome (global relative beta power) did not differ between groups. Still, infants in the massage group showed significantly higher alpha relative power in the central region, suggesting localized maturation-related enhancement in central networks [34]. Additionally, dose-dependent correlations linked higher session counts to increased relative beta/alpha/theta and decreased delta, indicating that regional spectral maturation indices were sensitive to cumulative tactile exposure.

In a separate preterm infant trial, longitudinal spectral analysis showed that massage moderated expected power declines and produced region-specific changes, including increased delta/theta power in central leads and decreased delta/alpha in temporal leads [35]. Between-group analyses identified time-by-group interactions for central delta and beta power, again implying that the central scalp region—broadly corresponding to sensorimotor and midline cortical areas—may be susceptible to enriched tactile/kinesthetic stimulation in preterm development. These regional patterns are not identical to adult somatotopy. Still, they support the broader conclusion that massage effects can be spatially localized and may preferentially engage central networks across life stages.

#### 3.1.9. Source-Localized EEG and DMN-Adjacent Hubs (Posterior Cingulate Cortex and Anterior Cingulate Cortex)

Only a small subset of the EEG massage literature used source reconstruction to move beyond scalp-level band power and infer the likely cortical generators of massage-related activity changes. Among the studies summarized here, two investigations applied exact low-resolution brain electromagnetic tomography (eLORETA) to resting-state EEG data. They reported alpha-band changes localized to midline cortical regions commonly discussed in relation to self-referential and affective processing, including the posterior cingulate cortex (PCC) and anterior cingulate cortex (ACC). These studies are particularly relevant to the reviewer’s concern about identifying brain targets such as the default-mode network (DMN) because the PCC is a canonical DMN hub and the ACC is frequently implicated in DMN–salience interactions and affective regulation. While these source-localized results do not establish network-level “DMN modulation” in the same way as fMRI connectivity analyses, they provide the most spatially specific EEG evidence in the dataset that massage can differentially engage DMN-adjacent regions depending on stimulation site and delivery mode.

##### Hand Versus Foot Massage in Elderly Long-Term Care: PCC Recruitment Differs by Massage Site

In a crossover study of elderly individuals under Japan’s Long-Term Care Insurance system (mean age ~82), participants received both hand massage and foot massage in counterbalanced order, with a one-week washout between sessions [39]. Each intervention lasted 15 min and used a standardized, non-reflexology technique with gentle stroking and oil to reduce friction. Resting-state EEG was recorded immediately before and after each session and analyzed using eLORETA, with the primary neurophysiological endpoint being changes in alpha-band (8–13 Hz) activity at the cortical source level. Subjective ratings of pleasantness, relaxation, and refreshment improved following both massage types and did not differ significantly between hand and foot massage, indicating comparable perceived benefit [39]. However, the source-localized EEG results revealed distinct regional signatures: hand massage was associated with increased alpha activity in the left insular cortex, whereas foot massage was associated with increased alpha activity in the posterior cingulate cortex, bilaterally [39].

##### Manual Versus Machine Massage for Occupational Leg Swelling: ACC Alpha Increase Is Specific to Hand Massage

A second eLORETA study examined 18 healthy middle-aged women (mean age ~47.5) with occupational leg swelling in a randomized crossover design comparing hand massage to machine massage applied to the right leg [47]. Both interventions were delivered for 15 min, and each participant received both conditions separated by a two-week interval. Peripheral outcomes showed that both hand and machine massage significantly reduced lower leg volume measured by water displacement, indicating that both were physiologically effective for edema reduction [47]. However, subjective ratings differed: hand massage elicited significantly higher ratings of pleasantness, relaxation, and refreshment compared with machine massage [47]. Resting-state EEG was recorded before and after each intervention, focusing on alpha-band activity, and eLORETA was used to estimate cortical sources.

The central neural finding was that hand massage, but not machine massage, increased alpha-band activity in the left anterior cingulate cortex (ACC) [47]. This result is significant for two reasons. First, it suggests that even when peripheral physiological outcomes are comparable, the cortical signature of manual touch may be distinct from that of mechanized stimulation. Second, the ACC is a midline region implicated in affective appraisal, integration of bodily state, and regulation of autonomic and emotional responses—processes that align closely with the subjective differences observed between conditions. The lateralization to the left ACC also parallels the broader pattern seen in several studies where massage shifts frontal/affective lateralization toward left-sided activity, although the ACC finding here comes from source modeling rather than a scalp asymmetry index. Taken together, these results suggest that manual massage may recruit affective midline circuitry more robustly than machine massage, potentially reflecting differences in sensory predictability, pressure dynamics, social-affective touch components, or subtle therapist-driven adaptation during the session.

#### 3.1.10. Source-Localized EEG and DMN-Adjacent Hubs (PCC/ACC)

Only two studies in the summarized EEG dataset used source reconstruction (eLORETA) to localize massage-related changes in cortical activity, but both identified effects in midline regions relevant to DMN-centered models, particularly the posterior cingulate cortex (PCC) and anterior cingulate cortex (ACC).

In an elderly long-term care crossover study (15 min sessions), both hand and foot massage increased subjective pleasantness/relaxation. Still, eLORETA showed site-specific alpha-band generators: hand massage increased alpha in the left insula, whereas foot massage increased alpha in the PCC bilaterally [39]. In a randomized crossover study of occupational leg swelling (15 min sessions), both hand and machine massage reduced leg volume, yet only hand massage improved subjective ratings and produced an eLORETA-detected increase in alpha activity in the left ACC; this ACC effect was not observed after machine massage [47].

#### 3.1.11. Entropy/Complexity-Based EEG Outcomes in Pain and Motor Paradigms

A distinct subgroup of studies quantified massage-related neural effects using entropy and complexity measures, rather than (or in addition to) conventional band power. These approaches treat EEG as a dynamical signal and assess changes in irregularity, predictability, and information content within the time series or within frequency-specific representations. Across the pain-focused and motor-task paradigms summarized here, entropy-based outcomes were consistently sensitive to massage, often showing post-massage reductions in complexity (though the affected rhythms and spatial distributions varied by cohort and analytic method). In addition, several studies combined entropy features with connectivity-sensitive indices and machine learning classification, demonstrating that massage-related state changes could be detected at high accuracy from EEG feature sets.

##### Skeletal Muscle Pain with Motor Imagery/Execution: Broad Post-Massage Reductions in Alpha-Band Complexity Across Task States

In a large cohort of participants with skeletal muscle pain (*n* = 71; mean age ~54), EEG was recorded using a 14-channel Emotiv EPOC+ system (128 Hz; 0.5–40 Hz band-pass) before and after a 30 min professional massage [36]. The protocol explicitly tested multiple functional states: quiet resting (eyes closed), motor imagery (left and right trunk bending), and motion execution (left and right trunk bending), with each state repeated post-massage. After artifact removal (including MEMD + CCA for EMG and wavelet-based ICA for ocular artifacts), signals were segmented into 4 s epochs and analyzed using sample entropy (SampEn) and permutation entropy (PermuEn), alongside additional features for classification (e.g., common spatial patterns).

The principal result was that SampEn and PermuEn values in the alpha band decreased significantly in nearly all EEG channels after massage, and this reduction was observed across all five experimental statuses (rest, MI left, MI right, ME left, ME right) [36]. Topographic maps presented in the study indicated that the reduction in signal complexity was widespread rather than focal. In parallel, the study reported changes in an inter-channel measure, the permutation disalignment index (PDI), with significant alterations particularly in the beta rhythm after massage, suggesting that complexity reductions occurred alongside detectable changes in coupling/coordination patterns [36].

The study also evaluated whether EEG could discriminate motor states using machine learning and deep learning models before and after massage. Using entropy-derived features and other representations, classification performance was high overall (e.g., BiLSTM AUC ~0.89 before and ~0.88 after massage), indicating that massage altered signal complexity while leaving task discriminability preserved mainly in the strongest models [36]. Additionally, comparisons between MI and ME indicated that ME generally produced higher SampEn values for delta/alpha/beta rhythms than MI, highlighting that complexity metrics were sensitive to task demands both before and after massage.

##### Lumbar Disc Herniation Versus Healthy Controls: Rhythm- and Group-Dependent Entropy Changes After Chinese Massage

A second study applied a broader entropy framework to evaluate immediate responses to a 15 min Chinese massage focused on the lower back and hips in lumbar disc herniation (LDH) patients (*n* = 26) and healthy controls (*n* = 24), again using a 14-channel Emotiv EPOC+ headset [37]. EEG was analyzed in canonical rhythms (delta/theta/alpha/beta) and processed through multiple entropy algorithms, including approximate entropy (ApEn), sample entropy (SampEn), wavelet entropy (WaveEn), Hilbert–Huang transform marginal spectrum entropy (HHTMSEn), permutation entropy (PE), fuzzy entropy (FuzzyEn), and inherent fuzzy entropy (IFE), as well as normalized energy measures. The authors also examined inter-channel coupling using PDI and trained machine learning classifiers to distinguish groups and massage states across different epoch lengths (1024, 512, and 256 points).

In LDH patients, the post-massage pattern emphasized changes in the delta rhythm, with decreases in multiple entropy measures (ApEn, SampEn, WaveEn, PE, FuzzyEn, IFE) coupled with a significant increase in delta energy, reported as predominantly left-hemispheric in topographic presentations [37]. In addition, theta and alpha rhythm energy levels significantly decreased after massage in the LDH group. In healthy controls, the directionality differed: delta entropy measures increased after massage. At the same time, alpha/beta complexity decreased, indicating that the same intervention produced group-specific complexity signatures rather than a uniform global response across participants [37]. Connectivity-sensitive analysis also diverged by cohort, with the study reporting that massage enhanced cortical connectivity in LDH patients but reduced it in healthy controls based on PDI outcomes [37]. Finally, classification analyses using entropy features achieved high accuracy (often >90%) in distinguishing LDH versus healthy participants and pre- versus post-massage states, supporting the discriminative value of entropy-based representations for capturing massage-induced changes in clinical pain contexts [37].

##### Specific Low Back Pain: Decreased Signal Complexity After Massage Using ApEn and HHT-Based Spectrum Entropy

A third pain cohort study focused on specific low back pain (SLBP) associated with disc herniation (L4–L5 or L5–S1) and quantified complexity changes after a 25 min massage session using two primary measures: Approximate Entropy (ApEn) computed on EEG time series and Hilbert–Huang Transform marginal spectrum entropy (HHTMSEn) computed on frequency-distribution representations [46]. EEG was recorded from 14 channels (Emotiv; resting state with eyes closed), and wavelet packet decomposition was used to extract rhythm-specific activity (δ 1–3 Hz, θ 4–7 Hz, α 8–13 Hz, β 14–30 Hz).

Across the overall EEG signals, both ApEn and HHTMSEn decreased significantly after massage in multiple channels, including frontal and fronto-central sites and extending to temporal, parietal, and occipital channels (the study highlighted significant changes in channels such as F3, FC5, T7, P7, O2, P8, T8, FC6) [46]. When analyzed by rhythm, ApEn reductions were significant in delta and alpha bands post-massage, indicating that these rhythms contributed strongly to the observed complexity decrease. HHTMSEn analyses additionally identified substantial reductions in beta rhythm entropy, suggesting that complexity changes were not confined to slow-wave activity and could involve higher-frequency dynamics depending on the entropy formalism applied [46]. Thus, within the same dataset, different entropy definitions emphasized partially overlapping but non-identical spectral contributors to the massage effect.

#### 3.1.12. Evidence Relevant to Dose–Response Relationships

Across the summarized EEG studies, explicit testing of dose–response relationships was uncommon, mainly because many investigations used single-session pre–post designs and reported massage exposure only as session duration (minutes) without systematic manipulation of intensity or cumulative dose. Nevertheless, several studies provided direct or indirect evidence relevant to dose–response, including (i) neonatal/preterm cohorts where cumulative exposure varied naturally or by protocol and was linked to electrophysiological maturation outcomes, (ii) multi-week adult programs where repeated sessions enabled comparison of early versus late responses (adaptation/attenuation of acute effects), and (iii) studies in which outcomes differed as a function of delivery mode or stimulation pairing (suggesting that “effective dose” may be shaped by modality composition rather than time alone). Importantly, the strongest dose–response evidence came from infant/preterm studies, while adult studies more often suggested non-linear adaptation of acute EEG markers over time.

##### Preterm/Infant Cohorts: Cumulative Tactile Exposure Correlates with Electrophysiological Maturation Indices

The clearest dose–response signal was reported in very preterm (VPT) infants receiving structured maternal massage in a randomized controlled trial spanning from 34 weeks’ gestational age to term-equivalent age (TEA) [34]. Infants in the intervention group were prescribed 15 min sessions twice daily, and an EEG was recorded at TEA during active sleep. While the primary endpoint—global relative beta power—did not significantly differ between massage and standard care groups, several results were directly relevant to dose–response. First, secondary analyses indicated localized group differences (higher alpha relative power in central regions) consistent with enhanced maturation. Second, and most importantly for dose–response, the study reported that the number of massage sessions received was positively associated with more mature spectral profiles: greater cumulative exposure correlated with higher relative power in beta, alpha, and theta bands and lower relative power in delta, a pattern interpreted as maturation-consistent. A per-protocol analysis—restricting the intervention group to infants who received at least 15 sessions—further strengthened these effects, yielding significant increases in beta and alpha indices compared with controls [34]. Thus, within the same trial, electrophysiological outcomes tracked not only assignment but also the realized “dose” of tactile stimulation, supporting a dose–response association rather than a binary treatment effect alone.

A second preterm EEG study also provided dose-relevant longitudinal evidence by comparing changes in spectral power over time in infants randomized to massage versus standard care [35]. Massage began around 10 days after birth and was delivered three times daily for ten days (10 min tactile stimulation prone + 5 min passive limb movements supine). EEG was recorded at approximately one week (T0) and four weeks (T1) of age during active sleep. The key dose-relevant finding was not expressed as an explicit “dose–response correlation,” but rather as a time-by-group interaction indicating that repeated massage exposure altered the developmental trajectory of EEG spectral power relative to controls. Controls showed significant decreases in global delta and alpha power from T0 to T1, consistent with expected postnatal trends; massaged infants did not show the same worldwide decline and instead exhibited region-specific changes (notably increased delta/theta power in central leads and decreased delta/alpha power in temporal leads). Between-group analyses identified significant interactions for global delta power and for central delta and beta power, indicating that massage altered the magnitude and/or direction of spectral change across the intervention window [35]. Although not framed as a graded dose model, these findings demonstrate that repeated exposure across days can shift the slope of electrophysiological development—an inherently dose-related phenomenon because it depends on cumulative stimulation over time rather than a single exposure.

Dose-relevant evidence was also present in a study using amplitude-integrated EEG (aEEG) in very-low-birth-weight infants [49]. The massage protocol (twice daily, nurse-delivered) was embedded within a larger randomized trial, and a subset of infants underwent prolonged (12 h) aEEG recording at 34 weeks postmenstrual age. Compared with controls, a higher proportion of massaged infants displayed continuous background patterns, and fewer showed delayed maturation scores. While the analysis was limited by sample size and did not quantify individual cumulative exposure as a predictor, the pattern of more mature aEEG backgrounds in the massage group is consistent with the concept that repeated tactile stimulation contributes to electrophysiological maturation. This study, therefore, supports the feasibility of using extended EEG-derived metrics as dose-sensitive outcomes in neonatal settings, even though formal dose modeling was not performed [49].

Taken together, infant/preterm studies provide the most direct evidence in the dataset that “dose” matters in EEG outcomes. Evidence appears in two complementary forms: (i) graded correlations between the number of sessions and maturation indices [34] and (ii) longitudinal time-by-group effects indicating altered developmental trajectories across repeated exposure windows [35], supported by convergent maturation trends in aEEG [49].

##### Adult Repeated-Session Programs: Attenuation of Acute EEG Responses Across Time Despite Stable Baselines

In adults, repeated-session designs did not generally model dose as a continuous predictor, but they did provide evidence that the electrophysiological response to a fixed session may change across a program—suggesting adaptation, habituation, or ceiling effects rather than linear dose–response scaling.

The aromatherapy massage trial (40 min sessions, twice per week for four weeks, a total of eight sessions) explicitly compared acute EEG changes early versus late in the program [29]. Shortly after a session early in the intervention, alpha activity increased, and delta activity decreased relative to pre-session values. However, the study noted that the magnitude of the acute EEG alteration after a single session was weaker in the final (eighth) session than in the first session. Importantly, resting EEG measured before sessions did not differ between the first and eighth treatments, and ANCOVA analyses did not show significant differences in basal EEG patterns after four weeks. Thus, in this dataset, increasing cumulative exposure did not translate into progressively larger resting-state EEG shifts; instead, it was associated with attenuation of the immediate post-session EEG response, while baseline pre-session EEG remained stable [29]. This pattern is dose-relevant because it suggests that repeated exposure can modify responsiveness to each session in a non-linear manner and implies that acute EEG markers may not be appropriate proxies for cumulative benefit without accounting for adaptation.

A workplace chair massage program (twice weekly over five weeks) also reflects repeated exposure. However, the study sampled EEG primarily at the first and last day of the program (pre-/post-session at each time point) rather than session-by-session [28]. Both massage and relaxation control groups showed increased frontal delta immediately after sessions at both time points, indicating a robust acute relaxation component not specific to massage alone. However, only the massage group showed decreases in frontal alpha and beta power and improvements in math computation performance, along with larger reductions in anxiety. Although the study did not explicitly test whether EEG effects strengthened with cumulative sessions, its design indicates that EEG and performance endpoints can remain detectable after repeated exposure across weeks, supporting the plausibility of longer-term intervention studies. At the same time, the absence of a formal dose model means that this study contributes indirectly to dose–response discussion by demonstrating that repeated programs can produce consistent measurable post-session EEG signatures at distant time points [28].

##### Dose as “Effective Stimulation”: Modality Composition and Delivery Mode as Determinants of Dose-Equivalent Outcomes

Several studies suggest that “dose” may be better conceptualized not only as minutes or number of sessions but also as effective stimulation intensity and composition, because adding or changing stimulation components can produce outcomes not achieved by massage duration alone.

In a crossover fatigue study using a mechanical massage chair, mechanical massage alone did not significantly alter EEG-derived fatigue indices (theta/beta, (theta + alpha)/beta). In contrast, the same mechanical massage paired with binaural beats and music produced significant reductions in these indices and improvements in selected cognitive measures [41]. Since session duration was held constant across conditions (20 min), the differential outcome implies that the effective “dose” in terms of neural impact depends on multimodal pairing rather than time. Similarly, manual versus mechanical delivery comparisons showed that while peripheral outcomes (e.g., swelling reduction) could be comparable across delivery modes, only hand massage produced cortical alpha increases in ACC and higher subjective relaxation/pleasantness [47], again suggesting that the quality and character of stimulation (human-delivered vs. machine-delivered touch) influences effective neural “dose” even when duration is identical.

Finally, the controlled intensity comparison (moderate-pressure vs. light-pressure vs. vibration) demonstrated that intensity class can reverse the physiological directionality of EEG change within an identical 10 min duration [30]. While this study is primarily an intensity manipulation, it is directly relevant to dose–response because it indicates that duration alone cannot define dose; pressure intensity is a dose dimension capable of producing qualitatively different neurophysiological states. Thus, the same session length can represent different effective doses depending on pressure and mechanoreceptive recruitment.

### 3.2. fMRI Studies

**Figure 3:**
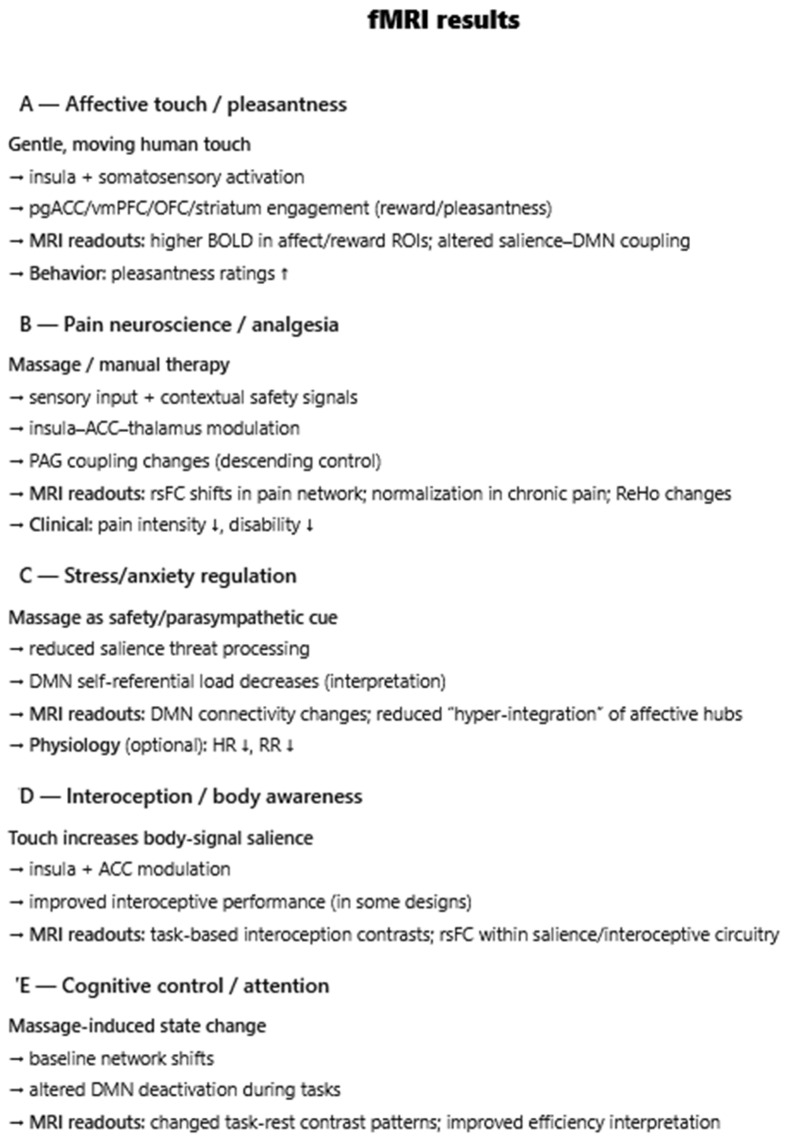
A graphical summary of the fMRI results.

The included fMRI studies are presented in Table 2. A graphical summary of the fMRI results is provided in Figure 3.

#### 3.2.1. Participant Characteristics

Across the 12 included fMRI studies [50,51,52,53,54,55,56,57,58,59,60,61], there were 409 participants: 326 healthy adults and 83 patients. Patient cohorts comprised 27 with cervical spondylosis [58], 27 with lumbar disc herniation [59], and 29 with chronic low back pain [61]; all three pain studies enrolled right-handed patients. Healthy samples (all right-handed when stated) included *n* = 40 (mean age 32.33) randomly assigned to Swedish massage, reflexology, wooden-object control, or rest [50]; *n* = 32 Japanese volunteers (mean age 22.2) in a double-blind reflexology expectancy design [51]; *n* = 25 (mean age 22) receiving reflex-area stimulation with a wooden stick [52]; *n* = 16 (mean age 30.2) in a pleasant-touch factorial paradigm [53]; *n* = 38 total in a pre/post-massage vs. rest design (27 massage, 11 rest; age not reported) [54]; *n* = 30 sex-balanced, mean age ~30, in a randomized double-blind crossover of foot reflexology vs. sham [55]; *n* = 20 male, right-handed (analyzed as 15 smokers, 5 nonsmokers; overall mean age 38.6, smokers 37.4, nonsmokers 38.8) during hallux reflexology [56]; *n* = 46 male (mean age 21.22) in a randomized, placebo-controlled pharmaco-fMRI study of oxytocin and massage (real and imagined; manual and machine) [57]; and *n* = 24 healthy adults assigned after induced myalgia to SMT (*n* = 6, mean 20.7), mobilization (*n* = 8, mean 21.2), or therapeutic touch (*n* = 10, mean 22.5) [60]. Matched healthy controls were also scanned in two patient studies: *n* = 27 (mean age 39) alongside cervical spondylosis patients [58] and *n* = 28 (mean age 31.8) alongside lumbar disc herniation patients [59]. Handedness was specified as right-handed in [50,52,56,59], and [61]; sex distribution was male-only in two cohorts [56,57] and explicitly balanced by sex in one [55]; other reports did not provide sex counts. Designs ranged from randomized parallel-group trials [50,60,61], double-blind or crossover trials [51,55,57], to pre/post or case–control rs-fMRI comparisons with healthy controls [54,58,59].

#### 3.2.2. Types of Massage Interventions

Interventions clustered into scanner-compatible foot/forearm protocols, brief pre-/post-massage, and multi-session clinical manual therapies. In [50], participants received right-foot Swedish massage, reflexology, wooden-object rubbing (tactile control), or no-touch rest while in the scanner; stimulation was delivered during rest and a Go/No-Go task. In [51], reflexological toe-base stimulation (bases of the 2nd/3rd toes of both feet) was applied under double-blind conditions with correct vs. pseudo-information about eye/shoulder reflex areas; touch was standardized within 5 s blocks. In [52], three left-foot reflex areas (eye, shoulder, small intestine) were each stimulated in counterbalanced runs using a wooden stick (5 s blocks), isolating somatotopic effects of putative reflex maps. In [53], a pleasant-touch (“touch massage”) factorial manipulated effector (human hand vs. rubber glove) and kinematics (moving vs. stationary) on the forearm, with 2.5 N pressure and 1.5 cm/s velocity; all four conditions were rated for pleasantness. In [54], the experimental group received a 5 min calf-and-foot massage (social touch) between two resting-state scans, whereas controls rested without touch. In [55], a randomized, double-blind, crossover study compared 10 min left-foot reflexology (FR) with sham massage (SM); rs-fMRI and well-being/physiology were obtained pre- and post- each session. In [56], hallux reflexology targeted three specific big-toe points (linked by theory to the head/neck) during scanning; each point was pressed for 45 s, alternating feet, in smokers and nonsmokers under double-blind conditions. In [57], within-subject pharmaco-fMRI combined intranasal oxytocin vs. placebo with real and imagined foot massage, contrasting manual (masseur) and machine delivery (four massage conditions: real/imagined × manual/machine). In [58], patients with cervical spondylosis underwent tuina (traditional Chinese manual therapy) every two days for two weeks (6 sessions), with clinical scales and rs-fMRI pre/post. In [59], lumbar disc herniation patients received an identical tuina course (6 sessions/2 weeks) with rs-fMRI before and after treatment. In [60], after exercise-induced low-back myalgia, participants were randomized (48 h later) to a single session of spinal manipulation (SMT), spinal mobilization (MOB), or therapeutic touch (TT); rs-fMRI was acquired pre-/post-immediately. In [61], chronic low back pain patients received osteopathic manipulative treatment (OMT) vs. sham across four weekly sessions, with fMRI at baseline (T0), after the first session (T1), and one month later (T2) during interoceptive (heartbeat) and exteroceptive (sound) tasks.

#### 3.2.3. Types of fMRI Measurements

Across the 12 studies, BOLD fMRI was acquired mostly at 3 T (Philips Achieva/Verio, GE MR750, Siemens Skyra) with conventional T2*-EPI sequences (typical TR 1.8–3.0 s, TE ≈ 30 ms, 3–5 mm slices), and once at 1.5 T (Siemens Magnetom Symphony). Ref. [50] combined resting and Go/No-Go task fMRI during right-foot stimulation on a 1.5 T system (EPI: TR = 3000 ms, TE = 40 ms, FA = 90°, 64 × 64, FoV 24 × 24 cm, 5 mm, 167 volumes/501 s; 3D-FLASH structural TR/TE = 11.2/21 ms, FA = 60°, FoV 26 × 26 cm, 256 × 256, 5 mm). Processing in SPM8 included realignment (motion parameters entered as regressors), T1 co-registration, MNI normalization (2 mm), 10 mm FWHM smoothing, 128 s high-pass filtering, canonical HRF GLM, first-level contrasts for Rest and Go/No-Go, and second-level one-sample *t*-tests with FWE correction; a functional localizer (Rest > Go/No-Go) defined sACC and RSC/PCC ROIs (MarsBaR), with PANAS positive affect and early reaction times as covariates. Ref. [51] acquired whole-brain 3 T EPI (TR = 2500 ms, TE = 30 ms, FA = 80°, 42 slices, FoV 192 mm, 64 × 64, 3 mm, no gap; 260 scans) plus Mprage (TR/TE = 6.5/3 ms, FA = 8°, FoV 240 mm, 1 mm). SPM8 preprocessing (realignment; co-registration; segmentation-based MNI normalization; 8 mm smoothing) fed a first-level GLM with 5 s blocks for R(correct), R(pseudo), L(correct), L(pseudo); global intensity normalization; 128 s high-pass. A second-level 2 × 2 rm-ANOVA (laterality × information accuracy) with FWE *p* < 0.05 voxel-wise used an inclusive postcentral gyrus mask (SPM Anatomy Toolbox); a 3-way rm-ANOVA added sex; ROI time-courses (MarsBaR, 4 mm spheres, plus a priori shoulder SI coordinates) yielded percent-signal-change plots. Ref. [52] (SPM2) discarded 5 dummy scans, realigned and slice-timed to the 16th slice, normalized to MNI, and 10 mm smoothed; a GLM modeled 5 s blocks for eye/shoulder/SI reflex areas with proportional scaling and 94 s high-pass; subtraction images isolated area-specific effects (e.g., (eye × 2) − (shoulder + SI)). One-sample *t*-tests constrained by an AAL postcentral gyrus mask and auxiliary task-vs-task masks (*p* < 0.05 uncorrected) were thresholded at FWE *p* < 0.05 voxel-wise; peak-based ROIs supported paired *t*-tests. Ref. [53] used GE 3 T EPI (TR = 2000 ms, TE = 30 ms, FA = 80°, 96 × 96, 37 oblique axial slices, 3.9 mm) during a forearm pleasant-touch factorial (human/rubber × moving/stationary) with SPM analysis. Ref. [54] collected pre/post resting-state runs on Philips 3 T (functional runs 360 images; TR = 2000 ms, TE = 30 ms, in-plane 2 × 2 mm, 4 mm slices, 76 × 74 matrix; T1 1 × 1 × 1 mm). SPM12 preprocessing preceded ROI-parcelation (AAL3, 144 regions); Pearson ROI-to-ROI edges (*p* < 0.05) informed graph-theory metrics (node strength, betweenness/eigenvector centrality, clustering, global/local efficiency). Ref. [55] (Philips 3 T, 32-ch) contrasted 10 min foot reflexology vs. sham with rs-fMRI (**EPI TR/TE = 2000/30 ms, FA = 90°, FoV 240 × 240 × 123 mm, 41 axial slices, 3 mm isotropic voxels, 300 volumes/10.8 min; MPRAGE 1 mm). CONN (v21a) preprocessing (realign, slice-timing, structural segmentation/normalization, functional normalization, outlier detection, smoothing) and denoising (linear confounds, 0.008–0.09 Hz) supported seed-based and ROI-to-ROI analyses for DMN/SMN/SN/ECN (Willard atlas) plus a pain-network of 23 ROIs (Harvard–Oxford; insula and VTA coordinates from literature). Ref. [56] delivered hallux reflexology during Siemens Skyra 3 T EPI (TR = 3000 ms, TE = 30 ms, FoV 200 mm, 5 mm slices) with high-res MPRAGE (TR = 1900 ms, TE = 2.30 ms, FoV 230 mm, 0.8 mm slices) and field-map distortion correction (TR = 737 ms, TE = 4.92 ms, FoV 320 mm, 3 mm). Ref. [57] used GE 3 T EPI (TR = 2000 ms, TE = 30 ms, 43 slices, 3.2 mm, FoV 220 × 220 mm, 64 × 64, FA = 90°) plus a 3D SPGR structural (TR = 6 ms, 1 mm) under SPM12 (discard 5 volumes; 6-parameter motion correction; co-registration; segmentation-based normalization; 8 mm smoothing). First-level design included six regressors (real/imagined manual vs. machine massage; rest before real), with head-motion covariates; second-level one-sample/paired *t*-tests used FDR *p* < 0.025 peak-level; 6 mm sphere parameter estimates supported correlations with basal oxytocin and trait autism. Ref. [58] (GE 3 T) performed rs-fMRI (**TR = 2000 ms, TE = 30 ms, 33 slices, 3.5 mm, 80 × 80, FoV 224 × 224 mm, 240 volumes) and T1 (TR/TE = 7.7/3.1 ms, 1 mm). DPARSF preprocessing (motion exclusion > 2 mm/2°, detrend, band-pass, nuisance regression) preceded ReHo computation (voxel-wise Kendall’s W over 26 neighbors), z-transform, and smoothing; ReHo-symptom correlations used VAS/NDI. Ref. [59] (Siemens Verio 3 T, 12-ch) acquired rs-fMRI (**TR/TE = 2000/30 ms, 43 interleaved axial slices, 64 × 64, FoV 220 × 220 mm, FA = 90°, 3.2 mm, gap = 0, 230 volumes) and MPRAGE (**TR/TE = 8100/3.1 ms, 1 mm). RESTplus v1.25 preprocessing (remove first 10 volumes; slice-time; realign; T1-based normalization; detrend; nuisance regression incl. Friston-24; 0.01–0.08 Hz filter; 6 mm smoothing for FC) yielded ReHo (K = 27 neighbors, standardized by global mean) and both static FC (seed-to-voxel) and dynamic FC (DynamicBC, sliding windows 50 TR/100 s, 98% overlap, step = 1 TR; variability as CV, z-standardized). Ref. [60] ran pre/post rs-fMRI on Philips 3 T, 32-ch (EPI TR = 2000 ms, 3 × 3 × 3 mm voxels, 38 slices, FOV 240 × 240 × 114 mm; scan length 5:42) with physiological recording; SPM12 preprocessing (slice-time; realign/reslice; co-register; MNI warp; 6 mm smoothing) plus ART outlier detection (global signal > 3 SD, translation > 0.5 mm, rotation > 0.01°; outliers censored in GLM). CONN denoising used CompCor, 0.01–0.1 Hz band-pass, and nuisance regressors (ventricle/white-matter signals, six motion parameters, ART outliers); ROI-to-ROI FC (Fisher-z) was computed among ACC, PCC, aINS, pINS, thalamus, SI, SII, PAG (9 mm spheres; 6 mm for thalamus/PAG). Ref. [61] combined task fMRI (IA heartbeat vs. EA sound) on Philips 3 T (EPI TR = 1.8 s, TE = 30 ms, 80 × 80, 3 × 3 × 3.5 mm, 185 volumes; T1 1 mm) with physiological recording; AFNI preprocessing (discard 5 vols; 3dDespike; slice-timing; motion align to vol-6; MNI normalization; 6 mm smoothing; high-pass 0.013 Hz) fed subject-level GLMs (two-gamma HRF; response periods modeled as no-interest). Group-level 3dMVM (2 × 3 × 2: OMT vs. sham × T0/T1/T2 × IA vs. EA) used FDR *p* < 0.05 correction; ROI analyses used independent coordinates (bilateral insula, cingulate, striatum, right MFG; 9 mm spheres).

#### 3.2.4. fMRI Results

##### Default-Mode-Related Effects of Massage and Touch

In [50], Swedish right-foot massage increased resting-state BOLD within two default-mode network (DMN) nodes—the subgenual ACC (sACC; x = 0, y = 30, z = −2) and retrosplenial/posterior cingulate cortex (RSC/PCC; x = 14, y = −52, z = 18)—identified by a group functional localizer (Rest > Go/No-Go). The sACC effect disappeared during the task, whereas RSC/PCC modulation persisted more broadly: reflexology mainly increased RSC/PCC at rest, and object massage increased RSC/PCC during the task. In a resting-state network study with a brief calf/foot massage [54], post-massage scans showed a relative reduction in connectivity within a 25-edge subnetwork (vs. rest-only controls), with hubs in the left anterior pulvinar thalamus and right pregenual ACC exhibiting lower clustering and node strength; whole-brain global metrics trended toward lower integration but were not significant. In a pharmaco-fMRI design [57], intranasal oxytocin selectively enhanced manual massage pleasantness and amplified DMN-related activations (medial prefrontal cortex, parahippocampal gyrus, PCC/precuneus). Two multi-session clinical studies further implicated medial DMN nodes: lumbar disc herniation patients showed reduced ReHo at baseline in the left orbital middle frontal gyrus (mPFC/DMN) with normalization after tuina [59], and cervical spondylosis patients exhibited abnormal ACC/PCC activity that shifted after tuina alongside symptom change [58].

##### Somatosensory Cortex and Somatotopy Under Reflexology

Two mechanistic reflexology experiments converged on SI. In [51], stimulating the (putative) eye reflex area at the bases of the 2nd/3rd toes consistently activated the left middle postcentral gyrus (face/eye representation) independent of expectancy (correct vs. pseudo-information) and sex; contralateral foot SI was also engaged. Lateralization deviated from canonical contralateral mapping (eye-area stimulation on either foot still favored left face-SI), arguing for a specialized cortical handling of reflexological touch rather than belief-driven effects. In [52], left-foot stimulation of eye, shoulder, and small intestine reflex areas yielded somatotopically appropriate SI peaks: eye → left middle SI (face/eye); small intestine → left superior SI (trunk/abdomen); shoulder showed a non-significant trend in right SI (upper-limb zone). Common activations reflected tactile foot processing. A clinical-style reflexology protocol [56] replicated contralateral pre- and postcentral gyrus responses to hallux stimulation and additionally recruited the thalamus and distributed cortical areas; smokers vs. nonsmokers did not differ.

##### Affective Touch, Reward, and Salience Circuitry

A factorial pleasant-touch study [53] showed that moving touch (1.5 cm/s, 2.5 N), regardless of effector, robustly engaged bilateral insula and SI. In contrast, human-hand moving touch uniquely recruited pregenual ACC (pgACC)—a region linked to hedonics, placebo/opioid analgesia, and pleasant warmth/taste—mirroring the highest pleasantness ratings. In [57], oxytocin potentiated manual (but not machine) massage effects in reward (orbitofrontal cortex, dorsal striatum, ventral tegmental area), social cognition (superior temporal sulcus, inferior parietal lobule), and salience/emotion (amygdala, ACC, insula), alongside DMN components; effects were independent of masseur gender and partially reproduced under imagined massage (with weaker motor-system involvement). Exploratory moderation indicated region-specific sensitivity (e.g., precuneus) by autistic-trait level, without altering the overall oxytocin effect pattern.

##### Resting-State Connectivity and Graph-Theory After a Brief Massage

Beyond regional activity, ref. [54] applied ROI-to-ROI connectivity (AAL3) and graph metrics. Following a 5 min calf/foot massage, the experimental group exhibited lower subnetwork connectivity than rest-only controls, notably around the pregenual ACC and anterior pulvinar thalamus, with reduced clustering and node strength in these hubs. Although global efficiency and related metrics showed no significant group differences, trends pointed to a post-massage downshift in network integration, consistent with a relaxation-related decrease in cognitive/affective processing load. Connectivity shifts also involved the putamen and the superior temporal cortex.

##### Pain-Related Networks and Manual/Osteopathic Therapies

An acute, single-session comparison of spinal manipulation (SMT), mobilization (MOB), and therapeutic touch (TT) after induced myalgia [60] found shared and modality-specific resting-state FC changes. Common to all groups, PCC–aINS coupling flipped from weakly inverse to weakly positive, pINS–PAG connectivity increased, and left SI–right pINS connectivity decreased, implying immediate reorganization spanning DMN–salience interfaces, descending pain modulation (PAG), and sensory-discriminative pathways. Modality-dependent effects included right SI–aINS (↑ SMT, MOB/TT) and right SI–PAG (↑ SMT/MOB, ↓ TT). Pain ratings dropped by ~4/101 across groups, without consistent correlations to FC changes; pressure pain thresholds were unchanged.

In multi-session cohorts, tuina modulated intrinsic activity and connectivity in chronic pain. In cervical spondylosis [58], baseline ReHo was elevated in the left middle temporal gyrus, left thalamus, bilateral ACC/PCC, and left inferior parietal and reduced in right gyrus rectus versus controls; after six sessions, ReHo shifted toward normalization (including increases in inferior temporal/occipital and cingulate/parietal regions), with symptom-linked correlations (e.g., baseline right gyrus rectus ReHo positively related to VAS/NDI; post-treatment Δpain negatively related to left inferior temporal ReHo). In lumbar disc herniation [59], baseline ReHo was reduced in the left orbital mPFC (DMN hub), alongside abnormal dynamic FC variance with the fusiform gyrus, orbital IFG, and precuneus; after tuina, ReHo increased, and dFC variance normalized, paralleling improvements in VAS and Oswestry disability scores.

An OMT trial in chronic low back pain [61] using interoceptive (heartbeat) vs. exteroceptive (sound) tasks showed time- and task-specific modulation: after the first session, interoceptive hubs (bilateral insula, ACC, striatum) tended to increase while the right MFG decreased; by one month (T2), these regions showed a marked reduction in activation relative to both baseline and sham, and heartbeat-tracking accuracy improved—consistent with greater processing efficiency within interoceptive/salience networks.

##### Reflexology vs. Sham and Nonspecific Tactile Effects

A randomized crossover [55] directly compared foot reflexology (FR) with sham massage (SM). Both equally altered intrinsic connectivity: DMN reductions in PCC/precuneus, SMN increases among tactile/motor regions, and mixed changes within a proposed Neural Network Correlates of Pain (e.g., insula, cingulate, thalamus). No FR–SM differences emerged in connectivity, physiology (heart/respiratory rates), or well-being; both improved well-being and lowered heart rate, indicating nonspecific relaxation/touch as the principal driver in healthy participants.

##### Integrative Summary

Taken together, massage and related manual/tactile interventions produce (i) state-dependent DMN modulation (sACC/pgACC/PCC/mPFC), (ii) robust SI engagement with somatotopic organization under reflexology (largely expectancy-independent), (iii) affective touch recruitment of insula/ACC with pgACC and mesocorticolimbic reward amplifications for pleasant human touch, enhanced by oxytocin, and (iv) resting-state reconfiguration across DMN–salience–sensorimotor–descending pain circuits (including PAG and thalamus). In chronic pain, multi-session manual therapies (tuina, OMT) normalize ReHo/FC in DMN and salience/interoceptive networks in tandem with clinical improvement. Where tested directly, specific reflexology effects did not exceed sham, underscoring the prominent role of nonspecific tactile/relaxation mechanisms in healthy cohorts.

### 3.3. fNIRS Outcomes

**Figure 4:**
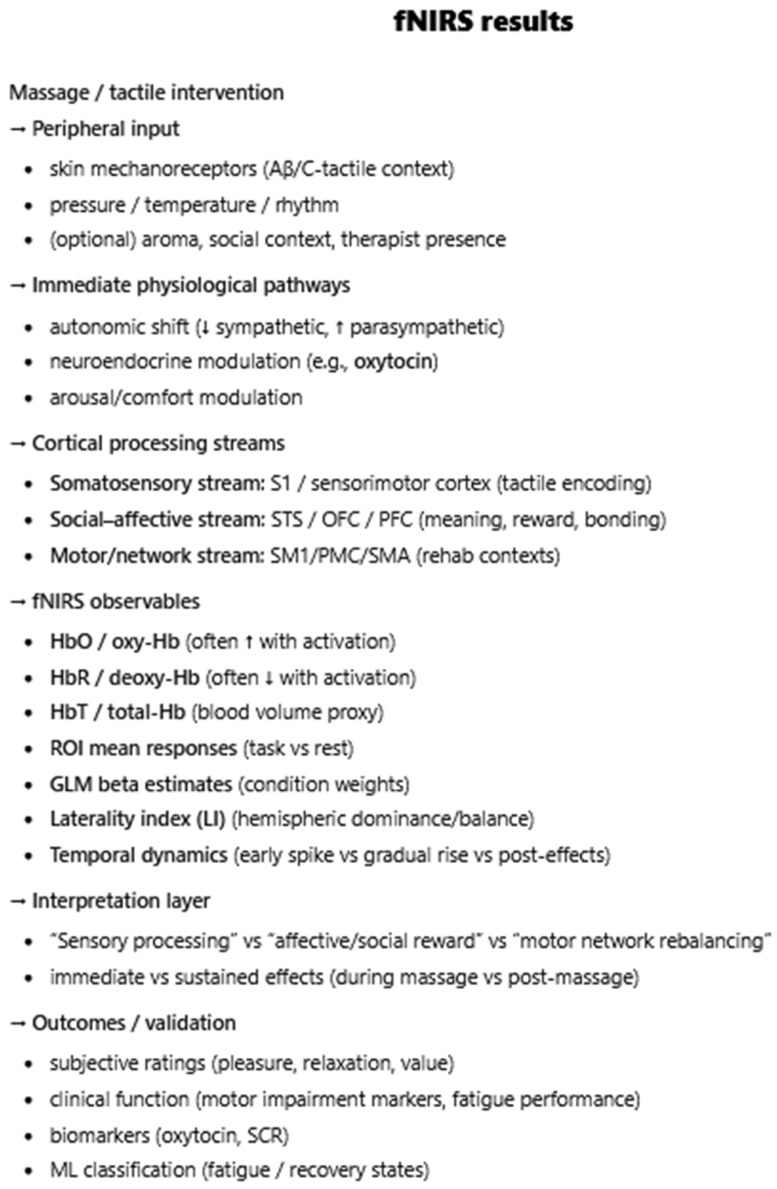
A graphical summary of the fNIRS results.

The included fNIRS studies are presented in Table 3. A graphical summary of the fNIRS results is provided in Figure 4.

#### 3.3.1. Participant Characteristics

Across the included studies, samples span the lifespan and clinical spectrum, from healthy infants to older adults and post-stroke patients. Study [62] enrolled 40 healthy Chinese men (mean age 21.78 years), all with prior foot massage experience; 4 were excluded for fNIRS technical issues, yielding n = 36 for analysis. Study [63] investigated four older women (all ≥65 years). Study [64] comprised 18 adults: 10 individuals with first-onset unilateral ischemic stroke and upper-limb motor dysfunction (mean age 58 years) plus 8 neurologically healthy, right-handed controls (mean age 49.25 years); sex distribution was not reported for either subgroup. Study [65] targeted pediatrics, reporting 20 full-term, healthy infants assessed in a randomized, within-infant design; an initial pilot involved two 11-week-old female infants; beyond the pilot, overall infant sex composition was not specified. Study [66] tested 10 healthy young adults (5 women, 5 men, ages 18–30) with no history of psychological disorders and no neurological medication.

#### 3.3.2. Types of Massage Interventions

Across studies, interventions varied by delivery mode (manual vs. mechanical), adjuncts (aroma, heat, auditory entrainment), target (general foot care vs. acupoint stimulation), and timing. In [62], each participant received two 10 min foot massages—hand-administered and machine-administered—with order counterbalanced, and participants were blindfolded; analyses modeled 20 s massage blocks and 10 s post-massage rest blocks within each 10 min session. In [63], three professional nurses delivered four foot-care conditions to each elderly participant: footbath, standard foot massage, footbath + aroma oils, and aroma-oil massage (blend: Lavandula angustifolia, Tea Tree, Ravensara, Palmarosa, Chamomile); stimulation alternated right/left foot in 60 s task/20 s rest cycles, repeated three times. In [64], therapists applied tui na at the Hegu (LI4) acupoint on both hands using the one-finger Zen technique at ~120 manipulations·min^−1^, following six cycles of 20 s rest → 20 s tui na → 30 s rest; comparisons emphasized affected vs. less-affected limbs in stroke. In [65], infants received, on separate occasions, a standardized massage protocol of 1 min segmental strokes over limbs and trunk and reflex locomotion (Vojta) therapy via pectoralis pressure points to elicit innate movement patterns; each session included a 5 min pre-baseline, a during-intervention recording, and a 5 min post-baseline. In [66], a heated mechanical foot reflexology massage (35 °C) was paired with binaural beats (tones of 256 Hz and 240 Hz → 16 Hz beta frequency) as a single combined session between pre- and post-fatigue cognitive testing.

#### 3.3.3. Types of fNIRS Measurements

Across studies, fNIRS was applied with precise, device-specific configurations, block paradigms, and analysis pipelines tailored to population and target cortex. In [62], a CW6 system (Techen, Milford, MA) with 27 optodes (12 sources, 15 detectors; 3.0 cm source–detector spacing) emitted 690/830 nm light and measured oxy-Hb/deoxy-Hb over six ROIs: bilateral orbitofrontal cortex (subdivided into lOFC, mlOFC, mOFC), bilateral medial S1 (foot/leg representation), and bilateral STS (aSTS/pSTS). Optodes were positioned to 10–20 landmarks, hair was parted, and a Polhemus 3D digitizer recorded coordinates. Data were processed in NIRS-SPM 4.1 with Wavelet-MDL de-trending to remove global physiological trends; a GLM modeled 20 s hand/machine massage events and 10 s post-massage rests via boxcars convolved with a canonical HRF. ROI analyses averaged channels within each ROI; contrasts (hand vs. rest, machine vs. rest, hand vs. machine) used paired *t*-tests with initial *p* < 0.05 (uncorrected) and Bonferroni correction across ROIs; correlations with behavior/questionnaires used bootstrapping, and mediation tested S1 → pSTS → OFC pathways by bootstrap. In [63], a Shimadzu FOIRE-3000 recorded oxy-Hb, deoxy-Hb, and total-Hb from 46 channels spanning the prefrontal cortex (Chs 1–22) and somatosensory cortex (Chs 23–46) during 60 s task/20 s rest alternations of right/left-foot stimulation, repeated three times. Channel-wise time courses were visualized as trend graphs, and moving-average smoothing supported comparisons between standard and aroma-oil massage, highlighting condition-specific fluctuations in selected prefrontal (e.g., Chs 8–9) and somatosensory (e.g., Chs 33, 35) channels. In [64], a NirScan-8000A (11 sources, 10 detectors; 730/850 nm; 19 Hz sampling) formed 32 channels over sensorimotor areas, with optode locations digitized via Patriot and channels parceled into PMC_ipsi/PMC_contra, SM1_ipsi/SM1_contra, SMA, and SAC. Focusing on oxy-Hb, preprocessing in NIRS-KIT and NIRS-SPM 3.2 applied TDDR motion correction, DCT high-pass (128 s) for drifts, and HRF smoothing. A GLM (boxcar convolved with a Gaussian HRF) yielded beta estimates per channel; significance was *p* < 0.05. Hemispheric engagement was quantified by a lateralization index (LI) = (contralateral − ipsilateral)/(contralateral + ipsilateral) computed from activated-channel counts. In [65], infant recordings used a Cortivision photoncap C20 (Baby kit) bilaterally over the motor cortex, with continuous HbO monitoring during 5 min pre-baseline, intervention, and 5 min post-baseline. SNIRF data were preprocessed in Homer3 using the Gemignani and Gervain pipeline A: intensity→OD (hmrIntensity2OD), OD→concentration (hmrOD2Conc), band-pass 0.01–0.7 Hz (hmrBandpassFilt), PCSrecurse motion correction (tMotion = 0.2, tMask = 1.0, StdevThresh = 50, AmpThresh = 0.1), and block averaging (hmrBlockAvg); optodes and HbO maps were projected to cortex with AtlasViewer, and the effects were tested via mixed ANOVA. In [66], a NIRSport 2 with 20 prefrontal channels (targeting DLPFC, OFC, VMPFC) followed 10–20 placements; raw signals underwent dark-noise testing, low-pass removal of instrumental noise, and a sixth-order Butterworth band-pass (0.05–0.3 Hz) to attenuate cardiac/respiratory components before modified Beer–Lambert law conversion to HbO_2_/HbR. From these time series, 34 temporal/spectral features (e.g., mean, RMS, skewness, kurtosis, entropy, FFT/CWT metrics) were extracted, reduced via PCA, and used for fatigue-state classification with multiple classifiers and an ensemble voter (10-fold cross-validation). Collectively, the measurement strategies emphasize ROI-targeted montages (social/reward, prefrontal, and sensorimotor networks), block-based paradigms, rigorous motion/physiological artifact control, GLM-based inference where appropriate, and, in one study, downstream feature engineering/ML to decode intervention-related cognitive state changes from fNIRS hemodynamics.

#### 3.3.4. fNIRS Results

##### Social-Reward Circuitry During Manual vs. Mechanical Foot Massage

Hand-administered massage produced significantly greater oxy-Hb increases in posterior STS (pSTS) and mediolateral OFC (mlOFC), whereas machine-administered massage failed to activate these regions and was associated with a reduction in mlOFC activity. The primary somatosensory cortex (S1, foot/leg area) showed no differential activation between massage types. ROI-wise GLM contrasts (hand vs. rest; machine vs. rest; hand vs. machine) at an initial *p* < 0.05 with Bonferroni correction across ROIs supported these effects. Across subjects, pSTS and mlOFC activation during hand massage correlated positively with pleasure ratings and willingness to pay; mlOFC activation also correlated positively with AQ scores. A mediation analysis indicated that S1 changes influenced mlOFC indirectly via pSTS (S1 → pSTS → OFC), consistent with sensory input routed through social-cognition circuitry before valuation [62].

##### Aromatherapy Foot Care vs. Standard Massage in Older Adults

With a 46-channel montage over prefrontal and somatosensory cortices, both standard and aroma-oil massage increased oxy-Hb (and total-Hb) and decreased deoxy-Hb in frontal channels (notably Ch13). Standard massage elicited weaker or more gradual oxy-Hb responses in the somatosensory cortex (e.g., Ch33), while aroma-oil massage produced larger, more variable oxy-Hb modulations. Moving-average trends showed standard massage yielding a gradual within-block rise, whereas aroma-oil massage evoked an early sharp oxy-Hb peak followed by a relaxation-related decline. Direct condition contrasts highlighted prefrontal Ch8–9 (greater oxy-Hb fluctuation with aroma) and somatosensory Ch33/Ch35 (aroma: gradual increase with cyclic variation; standard: more linear decline). Right–left differences were minimal; more somatosensory channels exhibited significant change overall, underscoring robust tactile engagement by foot care [63].

#### Sensorimotor Lateralization During Tui Na at Hegu in Stroke vs. Controls

During stimulation of the less-affected arm in stroke patients, contralesional SM1 predominated—mirroring healthy controls, who showed contralateral dominance regardless of the hand stimulated. In contrast, tui na on the affected arm lacked clear contralateral dominance. Channel-wise GLM (*p* < 0.05) and a lateralization index (LI = (contralateral − ipsilateral)/(contralateral + ipsilateral)) computed from activated channels confirmed significantly different LI values between affected and less-affected arms, with wide LI variability for the affected side. These patterns indicate disrupted hemispheric balance in sensorimotor circuits that fNIRS can resolve during real-time manual stimulation [64].

##### Infant Cortical Dynamics: Massage vs. Reflex Locomotion Therapy (Vojta)

Minute-by-minute HbO trajectories over the bilateral motor cortex differentiated the two interventions. Massage: bilateral HbO decrease (min 1) → bilateral increase (min 2); left-dominant increase (min 3) → bilateral decreases (min 4) → left-dorsal increase/right decrease (min 5); post-intervention increases in right hemisphere and ventral left. Reflex Locomotion: bilateral increase (min 1) → bilateral decrease (min 2, stronger left) → return to baseline (min 3) → bilateral increase (min 4, stronger right) → sharp right drop/left rise (min 5); post-intervention decreases confined to left. Mixed ANOVA confirmed main effects of intervention and time, indicating sharper, transient hemodynamic surges for reflex locomotion vs. more gradual/sustained engagement for massage [65].

##### Prefrontal Oxygenation and Fatigue Recovery with Mechanical Massage + Binaural Beats

Following a single session of heated mechanical foot reflexology (35 °C) paired with 16 Hz binaural beats, the prefrontal montage showed a marked increase in HbO_2_ with a concurrent decrease in HbR across DLPFC/OFC/VMPFC channels, consistent with enhanced regional cerebral blood flow and greater neuronal activity during repeated cognitive tasks. fNIRS time series (after band-pass filtering and MBLL conversion) yielded 34 temporal/spectral features; models trained on these features accurately discriminated fatigued vs. recovered states, with Random Forest the strongest single classifier (~94.6% accuracy) and an ensemble voter performing best overall, aligning the hemodynamic improvements with behavioral error reductions [66].

##### Cross-Study Synthesis

Manual, socially salient touch preferentially engaged social-reward networks (pSTS/OFC) without altering S1 differentially [62]; aroma augmented and reshaped prefrontal/somatosensory dynamics in elders [63]; lateralization metrics exposed stroke-related hemispheric asymmetries during acupoint stimulation [64]; infancy data resolved therapy-specific temporal signatures over motor cortex [65]; and prefrontal HbO/HbR shifts supported computational decoding of fatigue recovery after tactile–auditory intervention [66]. Collectively, fNIRS captured region-specific, time-resolved hemodynamic fingerprints of massage across populations and paradigms.

## 4. Discussion

Massage is a frequently performed form of rehabilitation. Many studies have reported the effectiveness of massage in various contexts. Massage can reduce pain, stress, anxiety, and depression and improve functioning in a range of neurological and psychiatric conditions. This mechanistic review synthesizes all studies examining the effect of massage on brain activity measured using three neuroimaging techniques: EEG, fMRI, and fNIRS. The number of included studies demonstrates that researchers intensively explore this topic. The results indicate that different massage techniques influence brain activity, often in various ways. The mechanisms of this effect are poorly understood. An analysis of the results, along with possible neural mechanisms, is presented below. It should be noted that any observed changes in brain activity with massage are correlational in nature. Neuroimaging outcomes reflect associative neural patterns and do not establish causality or direct mechanistic action. Therefore, conclusions about how massage “affects” the brain must remain tentative and interpretive, pending confirmation from controlled studies.

### 4.1. EEG-Based Massage Mechanisms

#### 4.1.1. Affective Touch, Interoception, and Psychological Effects

Affective (i.e., pleasant, socially meaningful) touch is now understood to be a distinct sensory channel: slow, gentle stroking at skin temperature preferentially drives unmyelinated C-tactile (CT) afferents that project to the posterior insula and limbic/autonomic hubs implicated in emotion and homeostasis. This framework—interoceptive touch engaging insula–ACC–hypothalamus circuits and modulating oxytocin, vagal tone, and stress systems—has been synthesized for clinical use under “touch medicine”. In massage EEG datasets, the recurring signatures (α facilitation with β/γ quiescence, left-frontal α asymmetry) align with this model.

Carefully controlled brush-stroking on hairy skin yields a somatosensory ERP that is velocity-tuned for pleasantness (inverted-U peaking in the CT range): a contralateral negativity ~400 ms after onset and later components that scale with perceptual affect [67]. Beyond time-locked ERPs, CT-optimal stroking modulates ongoing oscillations—relative α and higher bands—demonstrating that affective touch is captured in EEG as both evoked and induced activity, not merely subjective ratings [68]. Vicarious (observed) CT-optimal stroking recruits the posterior insula as well, tying the “affective” code to the interoceptive cortex even when touch is seen rather than felt [69]. These features provide a mechanistic context for massage protocols: slow strokes should elicit SI/SII ERPs and induce α/beta organization consistent with relaxed, low-threat processing.

The heartbeat-evoked potential indexes interoceptive state and attention. HEP amplitude increases when participants attend to cardiac sensations and subjectively report interoceptive changes; respiration gates this effect (larger during exhalation), revealing a tight link among breathing, cardiac signals, and cortical interoception [70,71]. Meta-analytic work confirms HEP as a convergent EEG marker of interoception across manipulations and tasks [72]. Causally, modulating the human insula with non-invasive focused ultrasound shifts HEP, underscoring the insula’s role as a driver of interoceptive EEG responses [73]. Interoceptive inference accounts further show HEP changes following expected vs. unexpected emotional stimuli, linking visceral predictions, emotion, and EEG [74]. Given massage’s CT-heavy input and respiration slowing, this literature predicts post-massage HEP enhancement (especially during exhalation) alongside α-dominant, low-arousal spectra.

FAA is a well-validated cortical proxy for approach–avoidance motivation [75]. Exogenous oxytocin—an endocrine hallmark of affiliative touch [76]—increases left-sided FAA during live eye contact, consistent with heightened approach/positive affect [77]. Massage studies frequently report leftward FAA shifts; the oxytocin→FAA linkage in independent EEG work strengthens the interpretation that affective touch engages pro-social approach systems rather than mere quiescence. Converging fMRI/behavioral reviews of moderate-pressure massage show engagement of the hypothalamus, amygdala, and ACC (endocrine/autonomic regulators), providing an anatomical substrate for the observed FAA and α effects [78].

Interpersonal touch (e.g., handholding) yields EEG inter-brain coupling that scales with analgesia—an objective marker that social touch coordinates brain states and reduces pain [79]. Replications with hyperscanning extend this to relationship context and alpha-band synchrony dynamics, reinforcing that affective touch shapes single-brain rhythms and dyadic regulation [80]. This helps explain why therapist-delivered massage, rich in social-affective cues, often produces stronger relaxation/analgesia signatures than purely mechanical stimulation.

Across non-massage EEG paradigms, (i) CT-optimal stroking evokes velocity-tuned somatosensory ERPs and reorganizes α/μ rhythms; (ii) HEP indexes interoceptive attention/state and is insula-dependent; (iii) oxytocinergic engagement shifts FAA leftward; and (iv) interpersonal touch synchronizes brains and attenuates pain. These findings provide a principled account of massage EEG: CT-rich, socially safe contact drives interoceptive–limbic circuits (insula/ACC/hypothalamus), elevates oxytocin and vagal tone, and yields a calm-alert cortical state (posterior/insula-linked α sources, β/γ downshift, leftward FAA) that supports analgesia and positive affect.

#### 4.1.2. Mechanotransduction and Cellular Signaling

Massage applies controlled shear and compressive forces to skin, fascia, and muscle that are transduced by (a) mechanosensitive ion channels on cutaneous and deep receptors, (b) integrin–cytoskeletal complexes on parenchymal cells (e.g., fibroblasts, myocytes), and (c) vascular/endothelial elements. At the peripheral sensor level, low-threshold mechanoreceptors (LTMRs) express PIEZO2, the principal mammalian touch transducer; in Merkel-cell complexes and other LTMR end organs, PIEZO2-mediated currents depolarize the afferent and/or accessory cells, driving reliable Aβ SA1 firing to the dorsal column–lemniscal pathway and onward to thalamocortical circuits. Accessory cells (e.g., Merkel cells) are now recognized as active mechanosensors that themselves express PIEZO2 and trigger transmitter release and afferent spiking under indentation, refining the gain and temporal precision of tactile coding [81,82,83,84]. In parallel, non-neuronal mechanotransduction in massaged tissue (integrins → FAK/ERK → transcriptional programs) modulates mitochondrial and extracellular-matrix turnover and suppresses pro-inflammatory cascades (e.g., NF-κB), a pathway repeatedly demonstrated in controlled massage-mimetic models (cyclic compressive loading) [85]. This tissue program explains observed reductions in secondary injury signaling and supports faster functional recovery after eccentric damage.

Mechanically driven afferent streams have predictable EEG signatures in humans, establishing the bridge from cutaneous mechanotransduction to macroscopic brain dynamics that we observe around massage:

(a) Frequency-following (SSSEP): Rhythmic vibrotactile input to glabrous skin elicits steady-state somatosensory evoked potentials in scalp EEG that track the stimulus frequency (≈20–30 Hz), with clear peaks over contralateral sensorimotor cortex; responses scale with attention and remain stable for ≥40 min when delivered pneumatically (i.e., purely mechanical input) [86,87,88].
(b) Sensorimotor mu/β modulation (ERD/ERS): Brief tactile pulses produce a canonical β (15–30 Hz) suppression ~300 ms post-stimulus followed by a β rebound ~700–800 ms, indexing transient disinhibition and subsequent re-inhibition of thalamocortical sensorimotor loops; mu (∼10 Hz) shows concurrent desynchronization during processing. These effects are robust across EEG/MEG and sensitive to alertness/attention [89,90,91,92].
(c) Gamma entrainment by mechanical input: Whole-body or focal vibrotactile stimulation at 40 Hz can entrain cortical gamma (primary somatosensory/motor) and has emerging therapeutic applications (e.g., gamma-entrainment trials for neurodegeneration), demonstrating that purely mechanical drive can organize fast cortical oscillations system-wide [93,94].

These non-massage EEG phenomena imply that massage’s temporal structure and force profile (stroke cadence, pressure cycles, intermittency) should shape cortical activity via the same somatosensory channels. Slow, quasi-periodic strokes (∼0.1–1 Hz) would be expected to phase-modulate ongoing alpha/mu and facilitate slow-wave components (δ/θ), while intermittent firmer compressions transiently suppress and then rebound β activity over contralateral sensorimotor regions—precisely the directionality reported in several massage EEG paradigms (α ↑ with β ↓; or δ ↑ with β/γ ↓ depending on site/modality).

#### 4.1.3. Neuroendocrine and Neurotransmitter Changes

Massage engages a coordinated neuroendocrine program—down-regulating the hypothalamic–pituitary–adrenal (HPA) axis while recruiting oxytocinergic, vagal, and (secondarily) monoaminergic systems—and these hormonal/neuromodulatory shifts have recognizable EEG correlates that are also seen in non-massage paradigms.

Moderate-pressure massage reliably produces a parasympathetic, anti-stress profile with heart rate/blood pressure reductions and decreased cortisol, especially when pressure and contact are sufficient to engage interoceptive/affective touch pathways. Mechanistic overviews and trials consistently place cortisol ↓ alongside vagal ↑ as primary endocrine signatures of hands-on massage [78,95]. These endocrine changes map to reproducible EEG features of low arousal: in massage datasets, relaxed-alert profiles (α ↑ with β/γ↓, or δ ↑ with β/γ ↓ depending on site/modality) accompany subjective calm. In the broader literature, exogenous cortisol administration increases delta–beta coupling at mid-frontal leads [96]—an EEG marker of stress integration [97,98,99]—connecting HPA output to cross-frequency dynamics directly; by symmetry, massage-related cortisol reductions predict the inverse shift (weaker δ–β coupling, less “stress binding”). Acute psychosocial stress also perturbs frontal alpha asymmetry (FAA), typically pushing activity rightward (i.e., alpha leftward) with recovery dynamics coupled to cortisol time-courses [100,101,102]; FAA thereby functions as a cortical readout of HPA state that moves in the opposite direction during relaxing, moderate-pressure massage.

Contemporary “touch medicine” models highlight C-tactile (CT) afferent input during slow/moderate stroking as a driver of oxytocin release [103] and interoceptive/limbic engagement (insula [104,105], ACC [106]) during caring touch—precisely the circuitry implicated in autonomic and endocrine control [76]. As we know, EEG studies without massage confirm that boosting oxytocin causally shifts cortical dynamics in directions that mirror massage: a single intranasal dose increases left-frontal approach-related FAA during live eye contact, indicating enhanced approach motivation/positive affect, the same polarity shift often seen after massage in the dataset in this review. At rest, oxytocin stabilizes EEG microstates (longer state durations) and down-weights an interoceptive/autonomic network (microstate C) in favor of an attention/fronto-parietal network (microstate D)—a millisecond-scale reorganization associated with anxiolysis [107,108,109]. Oxytocin has also been shown to alter EEG cross-frequency interactions and to covary with HRV [110,111,112], reinforcing the tight endocrine–autonomic–cortical coupling that massage appears to exploit via CT-optimal stimulation. In short, oxytocinergic facilitation provides a direct hormonal route by which affective touch lowers defensive autonomic set points and produces the calm-alert EEG motifs cataloged after hands-on massage.

Massage reviews frequently report mood improvement with parasympathetic dominance [113]; although monoamine assays are less consistently sampled in massage trials, the EEG consequences of serotonergic modulation are well characterized in pharmacology. A recent systematic review shows that SSRIs/SNRIs produce reproducible, agent-specific spectral signatures in resting EEG (with overlapping but distinct patterns across drugs), supporting a causal chain from 5-HT reuptake blockade → network-level oscillatory change [114]. Pretreatment alpha power/asymmetry also predicts SSRI response (responders show greater posterior alpha and a characteristic hemispheric pattern), demonstrating that serotonergic state is reflected in baseline EEG and that 5-HT-linked mood regulation has quantifiable oscillatory correlates [115]. These pharmaco-EEG findings give a mechanistic backdrop for massage outcomes: protocols that most strongly downshift arousal and normalize affect (vagal ↑, HPA ↓, oxytocin ↑) tend to yield alpha facilitation with β/γ quieting, i.e., the same direction of change that accompanies effective serotonergic interventions at the network level in depression. (Agent-specific exceptions are expected, hence the value of measuring EEG rather than assuming uniform pathways.)

The locus coeruleus–noradrenergic (LC–NE) system is a principal driver of cortical arousal; elevated NE tone promotes high-frequency activity and vigilance [116,117], whereas α2-agonism or LC suppression shifts EEG toward lower-frequency, less activated states [118]. Massage’s robust β/γ reductions (in many—but not all—protocols) fit a model in which tactile interoceptive input indirectly damps NE-mediated cortical gain via vagal/oxytocinergic pathways, complementing HPA down-regulation. This alignment between NE physiology and oscillatory signatures provides a second, non-serotonergic route through which massage can reduce fast-band activity.

Across endocrine systems, non-massage EEG experiments show (i) cortisol tracks cross-frequency coupling and FAA during stress; (ii) exogenous oxytocin pushes FAA leftward and stabilizes intrinsic EEG networks; and (iii) serotonergic manipulations leave distinct, replicable oscillatory “fingerprints”. In massage, convergent physiological evidence indicates cortisol ↓ with vagal ↑, recruitment of oxytocinergic/interoceptive hubs, and ANS quieting. This triad forecasts the EEG patterns you summarized (α facilitation, β/γ dampening, left-frontal asymmetry shifts, and selective δ/θ increases depending on site/modality). This neuroendocrine–EEG convergence yields precise table predictions for massage research: if a protocol most effectively reduces cortisol and raises oxytocin (and/or enhances vagal HRV), it should (a) decrease δ–β coupling and β/γ power, (b) increase posterior α (or stabilize α sources in insula/ACC/PCC depending on body site), and (c) shift FAA leftward—each effect measurable within minutes post-treatment.

### 4.2. fMRI-Based Massage Mechanisms

#### 4.2.1. Why “Dose–Response” Cannot Yet Be Derived from the Existing MRI Literature

The present MRI evidence base does not allow an empirically grounded dose–response curve (nor an “optimal” dose) for regulating specific networks such as the DMN. Importantly, this limitation is not simply due to “heterogeneity” in a generic sense; rather, it arises from several specific, structural features of the published designs and reporting practices represented in the summarized studies.

##### Massage “Dose” Is Multidimensional, but Most Dimensions Are Not Measured in Objective Units

Unlike pharmacological dosing, manual therapy dosing is inherently multicomponent. At minimum, “dose” can include (i) pressure/force (N or kPa), (ii) contact area (cm^2^), (iii) stroke velocity and frequency (cm/s; Hz) for moving touch, (iv) dwell time per site and total session duration, (v) number of sessions and inter-session interval, and (vi) spatial distribution (single-point vs. multi-site; local vs. regional). The majority of MRI studies summarized here do not quantify these variables in comparable units. In practice, many protocols report where stimulation occurred (e.g., toes, hallux, calf/foot) and sometimes how long the session lasted, but they rarely report force/pressure, contact area, or kinematics in a way that would permit scaling across studies.

This creates a fundamental barrier: even when session duration is known (e.g., short sessions such as 5–10 min in [54,55]), the mechanical exposure could differ substantially across studies and across practitioners. Only a subset of work explicitly standardizes mechanical parameters (e.g., controlled force and velocity stroking in touch massage [53]). Without such quantification, cross-study comparisons cannot determine whether stronger or weaker neural changes reflect higher or lower “dose,” or simply different unmeasured mechanical delivery.

##### The Literature Rarely Uses Parametric Dosing Designs (No Systematic Manipulation of Intensity × Duration)

Dose–response inference requires within-study variation in dose under controlled conditions. Across the summarized MRI studies, most designs compare different techniques or conditions (e.g., Swedish vs. reflexology vs. object touch vs. rest [50]; real vs. sham [55]; manual vs. machine and real vs. imagined massage with pharmacological modulation [57]) rather than dose levels of the same technique. Even when stimulation is delivered in blocks (e.g., 5 s stimulation epochs used for mapping in [51,52]), these designs are intended to identify somatosensory activation patterns. They are not implemented as dose manipulations that vary in intensity or total exposure.

Critically, none of the summarized studies presents a factorial manipulation of pressure intensity and session duration while holding other parameters constant. Therefore, the field currently cannot estimate a dose–response function (monotonic or non-monotonic), cannot test for thresholds, and cannot define an “optimal” point on such a curve for DMN outcomes.

##### “Dose” Is Strongly Confounded with Stimulation Site, Technique, and Social/Affective Context

Even if one attempted to treat “session duration” as a proxy dose variable, the studies are not comparable because “dose” is confounded with site and touch quality, both of which have independent neural consequences. The summarized MRI studies stimulate very different body regions—foot/toes/hallux [50,51,52,55,56,57], forearm [53], calf/foot [54], spine or manual therapy paradigms [60], and clinically targeted regions in tuina/OMT patient studies [58,59,61]. These sites differ in somatosensory representation, receptor density, and the balance of discriminative vs. affective afferent input.

In addition, multiple studies indicate that touch quality and social context modulate neural effects independently of dose. For example, differences between skin-to-skin touch and object-mediated touch are explicitly relevant in [50,53], and manual versus machine massage interacts with neuromodulatory state in [57]. These factors influence salience, reward valuation, and interoceptive processing, which in turn can alter DMN engagement. Consequently, two interventions of identical duration could plausibly produce different DMN outcomes due to differences in social meaning or affective touch engagement—not because of “dose.”

##### Acute Paradigms and Repeated-Course Interventions Measure Different Biological Phenomena

A further structural obstacle is the mixture of acute single-exposure experiments (seconds to minutes; often delivered in-scanner) and repeated-session clinical courses (weeks) within the same evidence base. Acute paradigms are typically designed to detect immediate BOLD changes or short-term rsFC shifts while minimizing motion and standardizing timing (e.g., localized foot stimulation in [50,51,52,55,56,57]). In contrast, multi-session interventions (e.g., tuina across two weeks in [58,59]; weekly OMT across four weeks in [61]) may induce slower processes—learning-like adaptations, changes in pain appraisal, altered interoceptive attention, or homeostatic network recalibration—whose neural signatures are not directly comparable to acute state effects.

Because the underlying biological time constants differ, it is not appropriate to combine acute and repeated-course findings into a single “dose–response” continuum without dedicated designs that bridge these timescales (e.g., acute dose manipulation + longitudinal follow-up).

##### Outcome Measures Differ Across Studies and Are Not Interchangeable as “Effect Size”

Finally, dose–response modeling requires consistent outcome definitions. In the summarized MRI literature, the dependent variables include region-specific BOLD activation (e.g., ROI activity in DMN hubs [50]), somatosensory mapping in SI [51,52,56], resting-state connectivity within and between canonical networks [55], graph-theoretic topology metrics [54], regional homogeneity (ReHo) and static/dynamic connectivity indices in pain populations [58,59], and task-evoked interoception contrasts across multiple timepoints [61]. These metrics capture distinct aspects of brain function (local amplitude, local synchrony, inter-regional coupling, network topology, or dynamic variability), and their magnitudes cannot be treated as a common “neural response” axis. As a result, even if dosing were comparable—which it is not—effect sizes would still not map cleanly onto a unified dose–response curve without endpoint harmonization.

#### 4.2.2. What the Current MRI Evidence Can Support: Bounded, Defensible Inferences

Although current MRI studies do not permit formal dose–response modeling or identification of an “optimal” pressure–duration combination, the available evidence is not merely too heterogeneous to interpret. When the scope of inference is explicitly bounded to what the designs actually test, the summarized studies support several defensible conclusions about (i) which brain systems are reliably engaged by massage/tactile interventions, (ii) how effects differ across acute versus repeated exposures, and (iii) which features of touch appear to moderate neural responses independent of dose. Below, we articulate these inferences in a form that is compatible with the data types and intervention structures used across the MRI studies.

A first bounded inference is that massage-related interventions can modulate intrinsic brain systems implicated in self-referential processing, affective regulation, interoception, and sensory integration, rather than influencing only primary somatosensory representations of the stimulated body site. This is most clearly demonstrated by studies that explicitly interrogate canonical intrinsic networks or network hubs. In [50], Swedish massage applied to the right foot during scanning altered activity within key nodes often discussed in relation to the DMN (sACC and PCC/RSC) during rest, suggesting that even localized peripheral touch can influence central systems linked to affective and self-referential processing. Complementing this, ref. [55] examined resting-state connectivity across several canonical networks (DMN, SMN, SN, ECN) and a proposed pain-related network. Both foot reflexology and a sham massage condition led to measurable connectivity changes within these networks; critically, DMN-related effects were observed in posterior components (PCC/precuneus). These observations justify the statement that DMN nodes are “massage-responsive” in the sense that their activity or connectivity can shift following tactile interventions, even when delivered to distal sites such as the foot. Importantly, however, the directionality and interpretation of these DMN shifts must remain bounded: ref. [50] reported increased BOLD signal in DMN-related hubs during rest, whereas [55] reported reduced DMN connectivity in posterior DMN nodes. The studies are not contradictory; rather, they illustrate that “DMN modulation” is not a single scalar phenomenon and can manifest as changes in regional amplitude, intra-network coupling, or network coupling depending on the measurement approach, baseline state, and analysis framework.

A second bounded inference is that somatosensory cortex engagement is robust and largely stimulus-driven in foot reflexology paradigms and that this component of the neural response appears relatively insensitive to cognitive suggestion under the conditions tested. In [51], reflexological stimulation to specific toe bases elicited consistent activation in SI (postcentral gyrus), including a region corresponding to the face/eye representation, and these patterns were not altered by pseudo-information about reflex maps. Similarly, ref. [52] reported somatotopically organized SI activation when stimulating reflex areas purported to correspond to the eye or the small intestine (with a weaker/non-significant pattern for the shoulder), alongside expected common activation in foot representation regions. The smoking cessation reflexology study [56] likewise demonstrated activation in contralateral precentral and postcentral gyri consistent with foot stimulation, alongside additional variable activations in other regions. Taken together, these studies support a conservative statement: foot-based reflexological stimulation reliably engages SI (and adjacent motor-sensory regions) in patterns consistent with tactile input, and at least some SI responses are not primarily driven by top-down belief manipulation. This does not validate reflexology claims about organ-specific therapeutic pathways, but it does show that reflex-area stimulation can evoke cortical responses beyond a simple “foot-only” representation in some paradigms and that these responses are detectable with fMRI under controlled conditions.

A third bounded inference concerns the affective and social dimension of touch, which appears to recruit additional circuitry beyond generic tactile processing and may act as a major moderator of neural outcomes that could otherwise be misattributed to “dose.” The touch massage factorial study [53] provides unusually clear evidence on this point because it controls force and velocity while manipulating the “humanness” of touch and the presence of movement. Across moving touch conditions, insular and somatosensory cortices were robustly activated, consistent with sensory and affective touch processing. However, the combination of human skin-to-skin touch with movement uniquely engaged the pgACC, a region implicated in reward and affective valuation. This pattern supports a bounded mechanistic inference: pleasant, socially salient touch recruits reward/affective-regulatory nodes (ACC subregions) in addition to core somatosensory–insula processing, and this recruitment depends on touch qualities that are not reducible to intensity or duration alone. The pharmaco-fMRI study [57] further reinforces this interpretation: intranasal oxytocin selectively enhanced the subjective pleasantness and neural responses to manual massage, but not machine massage, increasing activity across reward (orbitofrontal and striatal systems, ventral tegmental area), salience/interoceptive regions (insula, ACC, amygdala), social cognition systems, and DMN components (including mPFC, PCC, and precuneus). Even though oxytocin manipulation is not a “dose” factor, it demonstrates that neuromodulatory and social-affective context can substantially amplify or gate massage-related network engagement, including DMN involvement. This leads to a defensible caution: across studies, differences in DMN or ACC/insula responses cannot be interpreted as dose effects without accounting for whether the touch was socially meaningful, skin-to-skin, pleasant, or pharmacologically modulated.

A fourth bounded inference is that acute massage/touch can alter resting-state network organization, including nodes implicated in sensory integration and affective regulation, even after brief exposures. The network analysis study [54] examined rs-fMRI before and after a brief calf/foot massage and identified differences in network connectivity patterns relative to a resting control group. Notably, hubs including a thalamic pulvinar region and pregenual ACC showed reduced integration/strength post-massage compared with controls, which the authors interpret as consistent with a relaxation-associated reduction in integration within networks supporting attention and affective processing demands. While the directionality and functional meaning of “reduced integration” must be interpreted cautiously (as it could reflect decreased vigilance, altered arousal, or task-unrelated factors), the bounded inference remains: brief lower-limb massage is sufficient to shift resting-state network properties measurably, and these shifts involve thalamic and ACC nodes that are repeatedly implicated across affective touch and pain modulation literature. This converges with [55] in showing that short foot interventions can alter intrinsic network connectivity and with [50] in showing immediate modulation of DMN-related hubs during rest.

A fifth bounded inference becomes possible when considering repeated-session clinical interventions in pain populations: manual therapies administered over weeks can coincide with partial normalization of DMN- and pain-network-related indices, alongside clinical improvement, even though this does not establish a dose–response function. In LDH patients [59], baseline abnormalities included reduced ReHo in a medial prefrontal/DMN hub and altered dynamic functional connectivity variance with regions such as the precuneus; after six tuina sessions over two weeks, these indices shifted toward patterns more similar to healthy controls, and clinical pain/disability improved. In cervical spondylosis [58], pretreatment differences in ReHo included thalamus and cingulate regions frequently associated with pain processing and DMN/pain matrix overlap; tuina treatment was related to changes in these regions and correlations between regional indices and clinical measures. A strictly bounded inference from these data is that repeated manual therapy is associated with measurable changes in spontaneous activity indices within DMN-linked and pain-related regions in chronic pain patients, and that these changes can track symptom improvement. However, the inference must remain explicitly limited: because the studies do not parametrize mechanical intensity or isolate nonspecific therapeutic factors, these changes cannot be attributed uniquely to a specific “dose” component, nor can they specify an optimal schedule. They nevertheless support the plausibility that manual therapy can have central effects beyond peripheral biomechanics, particularly in conditions with established baseline network alterations.

A sixth bounded inference concerns immediate neurophysiological reorganization in pain-processing networks after manual therapy, observable even when behavioral effects are similar across treatment types. Study [60] examined resting-state functional connectivity among ROIs spanning the pain-processing network and descending modulatory systems (including PAG) following different forms of manual therapy after experimentally induced low back myalgia. Across interventions, there were consistent connectivity shifts involving PCC–insula coupling and posterior insula–PAG connectivity, as well as changes in cross-hemispheric sensory–insula coupling. Treatment-dependent differences were also observed in specific connections (e.g., SI–insula and SI–PAG patterns differing across manipulation vs. mobilization vs. therapeutic touch). These data justify a bounded mechanistic statement: manual therapy can rapidly alter functional coupling among nodes implicated in sensory-discriminative pain processing (SI/SII), interoceptive salience (insula), self-referential/DMN-related hubs (PCC), and descending modulation (PAG), and distinct manual therapy modalities can produce partially distinct connectivity signatures even when short-term pain ratings do not clearly differentiate them. This supports the idea that neuroimaging may be sensitive to mechanistic differences not captured by coarse behavioral endpoints in small samples, while simultaneously underscoring that “dose” cannot be inferred because the interventions are not defined by quantifiable pressure/duration parameters.

Finally, the OMT trial [61] supports a further bounded inference about interoceptive network modulation over repeated sessions, which is mechanistically adjacent to (and often interacting with) DMN regulation. In chronic low back pain patients, OMT relative to sham modulated activation in the bilateral insula, ACC, striatum, and right middle frontal gyrus during interoceptive tasks across timepoints, alongside improved heartbeat perception performance. This provides convergent support that repeated manual therapies can influence insula–ACC–frontal systems involved in interoception and salience allocation, which are frequently implicated as mediators of affective touch and pain modulation. While not a direct DMN-only endpoint, this pattern supports a bounded integrative view: manual therapy may influence network balance among DMN, salience/interoceptive, and sensorimotor systems, consistent with the diverse network-level findings. Again, this does not specify an optimal dose; instead, it supports a mechanistic framework in which touch-based interventions can shift how the brain integrates bodily signals, affective valuation, and self-referential processing.

#### 4.2.3. Peripheral Afferents → Cortical Targets: Fast Aβ Touch and Slow CT–Affective Touch

Massage engages two partly distinct tactile streams whose convergence helps explain its characteristic mix of “precise” and “soothing” effects on the brain. First, fast myelinated Aβ low-threshold mechanoreceptors (LTMRs) in skin and subcutis (Merkel/SA1, Meissner/RA1, Ruffini/SA2, Pacinian/RA2) transduce indentation, shear, stretch, and vibration. Their input ascends the dorsal column–medial lemniscus (DCML) pathway to the ventrobasal thalamus (VPL/VPM) and then to the contralateral primary/secondary somatosensory cortices (SI/SII), where it is represented in a finely ordered somatotopy. Ultra-high-field fMRI resolves these orderly maps with sub-millimeter precision for digits, toes, and the foot/leg, and attention or task demands can modulate gain within these maps. Thus, any localized kneading or gliding stroke in massage will strongly recruit SI/SII in a body-part–specific manner via Aβ signaling [119,120,121,122,123].

Running in parallel is a slow, unmyelinated “affective touch” channel carried by C-tactile (CT; C-LTMR) fibers, abundant in hairy skin but largely absent from glabrous skin. Microneurography shows CT units are velocity-tuned—responding optimally to gentle stroking around 1–10 cm/s—and temperature-tuned to skin-warm (~32 °C) contact; perceived pleasantness covaries with CT firing. In a rare individual lacking large-fiber (Aβ) function, CT activation induced a faint but distinctly pleasant tactile sensation, supporting a dedicated hedonic channel. These properties map neatly onto slow, warm strokes commonly used in massage [105,124,125,126,127].

At the cortical level, CT-optimized stimulation reliably recruits posterior/mid-insula, with somatotopic organization for gentle touch, and extends into a broader social-touch network (anterior insula, pSTS) as affective meaning increases. Multiple fMRI studies demonstrate velocity-sensitive insular responses that track pleasantness, while lesion work links right insula integrity to intact tactile pleasure. These observations explain why slow, caress-like massage strokes preferentially engage insular circuitry implicated in interoception and affect [106,128,129,130,131].

Conduction and routing further differentiate the streams. Aβ signals travel rapidly through DCML to SI/SII (supporting precise spatial/discriminative aspects of massage). In contrast, CT signals project via lamina I spinothalamic pathways toward thalamo-insular routes (with debate about specific thalamic relays such as VMpo), aligning CT input with homeostatic/interoceptive targets. Recent human and translational work confirms a C-fiber spinal pathway for pleasant touch without invoking noxious input, reinforcing that soothing massage taps a bona fide affective tactile channel [132,133].

Crucially, modern experiments indicate these streams interact. Peripheral A-fiber blockade in healthy adults nearly abolishes the pleasantness of both CT-targeted brushing and deep pressure, showing that explicit hedonic appraisal depends on intact A-fiber input even when CTs are stimulated. Conversely, deep-pressure touch—mediated primarily by Aβ receptors—can be calming and pleasant, complementing CT-optimized stroking in typical massage routines. Together, this Aβ–CT convergence provides a mechanistic basis for massage: spatially precise, depth-dependent activation of SI/SII coupled to affective, insula-centered coding of slow, warm strokes, yielding an integrated sensory–hedonic signal that sets the stage for downstream modulatory effects [134,135].

#### 4.2.4. Affective Valuation and Reward: OFC–Striatal Coding of Pleasant Touch (And Oxytocin as a Gain Control)

Pleasant social touch—a kind characteristic of slow, warm, skin-to-skin stroking and gentle pressure—engages a valuation circuit centered on the orbitofrontal cortex (OFC) and ventral striatum (nucleus accumbens, NAcc). Classic fMRI work showed that, holding physical intensity constant, affective touch (e.g., velvet) drives OFC responses more strongly than affect-neutral touch (e.g., wood), demonstrating that OFC codes hedonic value rather than simple somatosensory features; dissociable cingulate territories also track pleasant versus painful touch [136,137]. Building on this, human and translational reviews place OFC at the apex of a multimodal reward system that assigns value to primary reinforcers (including touch) and updates those values with context and learning—functions tightly coupled to ventral striatal mechanisms for motivation and reinforcement [138,139]. In parallel, ventral striatal responses participate in reward’s wanting/anticipation component during social and tactile contexts (e.g., romantic caress anticipation), linking motivational drive to subsequent sensory pleasure [140,141].

The bridge from skin to reward is provided by affective touch pathways (e.g., CT-afferent input) that reach the insula and then interface with OFC–striatal valuation. Multiple fMRI studies show that the subjective pleasantness of slow stroking is reflected in cortical activity—spanning posterior/mid-insula and extending into OFC—with behavioral choice and preference tracking these neural signals. Moreover, even the primary somatosensory cortex contributes to discriminating the pleasantness of a caress when social meaning is strong. Together, these findings map a route by which massage-like touch acquires hedonic value in the cortex and striatum [106,142,143].

Within this valuation scaffold, oxytocin (OXT) operates as a context-sensitive gain control for social touch. In a randomized, placebo-controlled pharmaco-fMRI study, intranasal OXT selectively increased pleasantness ratings for interpersonal touch and amplified activity in OFC, pregenual ACC, insula, and precuneus—effects that depended on social context and were attenuated with higher autistic traits [144]. Convergent human work further shows OXT boosts OFC responses and perceived pleasantness to affective touch and enhances coupling between reward and socioemotional networks, indicating a systems-level potentiation rather than a purely sensory effect [145,146]. Mechanistically, animal and cross-species evidence reveal that OXT gates social reward by acting within NAcc and midbrain dopamine circuits: coordinated OXT–serotonin signaling in NAcc is required for social reward, and OXT increases activity of VTA dopamine neurons projecting to NAcc—providing a cellular route for OXT to heighten the motivational and hedonic impact of affiliative touch [147,148]. Human syntheses align with this picture, highlighting OXT–dopamine interactions across the dorsal/ventral striatum and related hubs in social reward [149].

Finally, clinical and individual-difference data underscore that this OFC–striatal–OXT axis shapes how touch is valued. Major depression shows blunted striatal responses to social touch, and higher autistic traits can dampen OXT’s facilitatory effects—helping explain variability in touch-based interventions and supporting the view that manual therapies leverage valuation and motivation systems, not just somatosensation [144,150].

#### 4.2.5. Interoception and Salience: Insula–ACC as a Hub for Body-State Regulation

Massage-related calming and analgesic effects can be framed as consequences of interoceptive updating within the salience network (SN)—a large-scale system anchored in the anterior insula (AI) and dorsal/rostral anterior cingulate cortex (dACC/ACC) that detects biologically relevant signals and coordinates autonomic and cognitive set. Convergent fMRI shows that sensitivity to internal bodily signals (e.g., heartbeat detection) tracks activity and morphology in the right anterior insula, while the ACC co-activates as part of a visceromotor control loop; these regions form the cortical apex of interoceptive awareness [151]. The SN is anatomically and functionally dissociable from default mode and executive networks and is specialized to switch the brain toward internal regulation or goal pursuit depending on bodily salience—an operation causally linked to the right fronto-insular cortex [152,153]. Classic neuroanatomy and modern meta-analyses place AI/ACC within the central autonomic network (CAN) that couples cortex with hypothalamus, brainstem, and spinal autonomic outflow, explaining why changes in bodily state (e.g., parasympathetic upshift during soothing touch) are tightly yoked to insula–ACC activity [154,155].

Mechanistically, interoceptive predictions from agranular visceromotor cortices (including ACC/AI-adjacent regions) are thought to guide autonomic/allostatic set points, with ascending visceral signals used to update prediction error. This account situates massage-evoked slow, warm tactile input as a safety signal that reduces threat prediction and autonomic load [156]. Resting-state and task fMRI linking heart rate variability (HRV) to brain dynamics support this view: spontaneous HRV fluctuations covary with insula-centered connectivity and DMN coupling, while combined HRV–fMRI shows cardiovagal indices map to the hypothalamus, PAG, thalamus, posterior insula, and prefrontal/ACC—canonical CAN/SN nodes through which massage-induced parasympathetic dominance would propagate [157,158].

The AI–ACC hub is not only interoceptive but also regulatory. SN engagement initiates network switches that downshift self-referential DMN activity during states of relaxation and bodily safety; dynamic causal and review work emphasize AI’s role as the gatekeeper of such transitions [159]. Social buffering paradigms demonstrate the same circuitry in action: simple handholding during threat reduces right AI and hypothalamic responses, illustrating how affiliative touch dampens salience-tagged threat processing—precisely the context in which therapeutic touch operates [160]. Interoceptive attention and training (e.g., mindfulness) can recalibrate this hub, with longitudinal fMRI showing altered insula recruitment and connectivity during breath-focused interoception and attenuated insula responses to aversive interoceptive challenges—evidence that top-down modes can tune the same circuits that massage engages bottom-up [161,162].

Finally, large-scale syntheses argue that interoception and allostasis are implemented by a distributed network centered on the insula–ACC that coordinates peripheral physiology with perception and affect—offering a principled route by which slow, pleasant, socially meaningful touch can rebalance autonomic tone, lower vigilance, and facilitate pain control without invoking modality-specific “massage” mechanisms [163].

#### 4.2.6. Default-Mode Network (DMN) Rebalancing Under Relaxation and Safety Signals

Across non-massage paradigms that induce calm or perceived safety—mindfulness/meditation, slow breathing–vagal stimulation, sensory deprivation (“floatation”), and supportive social contact—fMRI consistently shows state-dependent rebalancing of the DMN (medial prefrontal cortex, posterior cingulate/precuneus) via salience–autonomic mechanisms. Experienced meditators exhibit reduced DMN activity and altered coupling during practice and even at rest, with convergent EEG–fMRI and replication in mindfulness-based training and neurofeedback studies, implying a shift away from self-referential/ruminative processing under relaxation [164,165,166,167].

Mechanistically, the salience network (SN)—anchored in the right anterior insula (rAI) and dorsal/rostral ACC—is positioned to switch the brain between internal mentation (DMN) and externally oriented executive control. Causal and intrinsic connectivity work shows rAI-driven gating of DMN executive transitions, framing pleasant, low-threat bodily input as a trigger for SN-mediated deactivation or reconfiguration of medial DMN hubs [152,153].

Autonomic evidence links this switch to parasympathetic (vagal) dominance. HRV—an index of vagal tone—covaries with insula/ACC and DMN dynamics at rest; newer studies explicitly relate insula–DMN connectivity to HRV, supporting an insula-centered relay between bodily calming and DMN rebalancing. Slow breathing enhances vagal activity and, together with respiration-linked fluctuations, modulates resting-state connectivity patterns that include the DMN [168,169,170,171].

Direct vagal afferent stimulation underscores causality: transcutaneous auricular VNS modulates DMN connectivity in clinical cohorts and healthy volunteers, with effects observed in medial hubs and network topology, consistent with a bottom-up route by which “safety signals” reorganize intrinsic activity [172,173].

Sensory downshift paradigms converge on the same motif. Following Floatation-REST (reduced exteroceptive and proprioceptive input), resting-state scans show decreased coupling between somatomotor and DMNs, implying that bodily quieting lowers DMN coupling to sensory systems—an effect directionally similar to relaxation after soothing touch [174].

”Safety” conveyed socially also rebalances large-scale dynamics. Classic handholding work shows attenuated threat responses in regions bridging salience/autonomic control (right AI, hypothalamus). At the same time, pain studies reveal that mind wandering away from pain reduces DMN deactivation and tightens DMN–PAG interactions, linking internal mentation to descending antinociception—both consistent with a calm-state DMN profile during affiliative contexts [160,175].

Finally, subcortical controllers provide infrastructure for DMN retuning. The thalamus (including the pulvinar) functions as an integrative hub with strong coupling to DMN nodes; human 7T mapping further clarifies DMN–subcortical connectivity, offering plausible conduits through which gentle bodily states alter cortical default-mode dynamics [176,177,178].

#### 4.2.7. Descending Pain Control: rACC/vmPFC–PAG Axis and Context/Expectancy

A central route by which massage-like touch can lessen pain is recruitment of the brain’s descending pain-modulatory system (DPMS)—in humans, most consistently indexed by coupling between rostral anterior cingulate/ventromedial prefrontal cortex (rACC/vmPFC) and the periaqueductal gray (PAG), projecting via the rostral ventromedial medulla to the spinal dorsal horn. Foundational neuroimaging work on placebo analgesia demonstrates that expectancy-driven relief engages rACC and brainstem nodes and is opioidergically mediated: naloxone reduces both behavioral analgesia and rACC/PAG responses, while prefrontal signals increase during anticipation and pain-evoked activity diminishes in nociceptive targets (insula/ACC/thalamus). These findings establish that top-down cognitive context can open an endogenous, opioid-sensitive rACC/vmPFC→PAG gate to dampen nociceptive processing—precisely the circuitry a soothing, supportive manual context is poised to activate [179,180,181].

Anatomical–functional syntheses and meta-analyses of human PAG imaging further solidify its role as a convergence hub for pain, autonomic control, and affect; ultra–high-field brainstem fMRI now resolves PAG–RVM dynamics during placebo/nocebo, underscoring a brainstem locus through which cognitive–affective state shapes nociception. At the distal end of the pathway, spinal cord fMRI shows that a placebo reduces dorsal horn responses to identical noxious input, confirming effective descending inhibition at the primary relay. Together, these results map a full top-down cascade—from prefrontal appraisal to brainstem control to spinal gating—through which relaxing interpersonal touch and supportive context can modulate pain [182,183,184].

Contemporary models describe this control in predictive-coding terms: prefrontal systems encode relief expectations/safety predictions that bias ascending nociceptive inference, with rACC/vmPFC–PAG pathways implementing the descending component. Causal and computational work shows that expected value signals in the prefrontal cortex modulate both brain and spinal activity; more broadly, reviews integrate DPMS function with cognitive/affective modulators (attention, reappraisal, anxiety) that are directly relevant to the calm, affiliative milieu of manual therapies. In chronic pain, aberrant PAG–vmPFC connectivity at rest highlights a trait-level shift in this axis that successful interventions can normalize—again consistent with a mechanism where reassuring touch helps restore top-down inhibitory tone [185,186,187,188].

Social and affiliative touch provides an independent window onto the same circuitry. In fMRI, handholding by a trusted partner attenuates threat responses in the right anterior insula and hypothalamus, with stronger relationship quality conferring larger neural dampening; dyadic hyperscanning shows that touch-related pain relief covaries with inter-brain coupling, implicating socio-affective regulation rather than mere distraction. Complementing this, a recent fMRI study using slow, CT-optimal skin stroking reported reduced pain and implicated insula–PAG mechanisms, dovetailing with the idea that pleasant social touch can access descending control. These social-context data align with the interpersonal setting of many massage encounters, where expectancy, trust, and safety cues likely potentiate rACC/vmPFC–PAG gating [79,160,189].

In sum, convergent evidence across the brain, brainstem, and spinal cord supports a context-dependent rACC/vmPFC→PAG pathway as the principal human DPMS route; it is tuned by expectancy, safety, and affiliation and demonstrably capable of reducing nociceptive transmission at the spinal level. By cultivating precisely these states—predictable, safe, socially supportive touch—massage plausibly leverages this axis to achieve analgesia without invoking modality-specific mechanisms [190].

#### 4.2.8. Thalamocortical Hubs (Pulvinar/Mediodorsal) and Large-Scale Network Configuration

A non-massage-specific route by which touch can reshape brain state is via thalamocortical hubs—notably the pulvinar and mediodorsal (MD) thalamus—that coordinate information flow among large-scale cortical networks (DMN, salience, executive, sensorimotor). Human and cross-species work shows the thalamus functions as an integrative hub whose nodal centrality supports both within-network cohesion and between-network routing, positioning it to rebalance global configuration during shifts toward relaxation or vigilance [178,191].

The pulvinar is especially suited to regulate cortical communication under changing behavioral demands: causal and correlational evidence indicates it synchronizes and gates information transmission between cortical areas according to attentional priority, thereby modulating effective connectivity across distributed visual–parietal–frontal systems. Such hub-like control provides a mechanism for quiet, low-threat contexts to down-weight external orienting and sensory drive [192,193].

The MD thalamus exerts complementary control over prefrontal dynamics. Converging reviews, human intracranial electrophysiology, and lesion/electrical-stimulation studies show MD–PFC loops organize low-frequency rhythmic coupling and regulate frontal–frontal interactions that underpin cognitive set and flexible behavior—precisely the substrate expected to shift when the organism transitions from vigilance to safety [194,195,196].

Subcortical influence extends to the default-mode network (DMN). High-resolution mapping and modeling identify consistent thalamic participation in DMN connectivity and demonstrate that subcortical nodes dynamically modulate DMN expression and switching with other systems; this provides a route by which bodily calming can reduce DMN coupling to sensory/executive networks while preserving internally oriented regulation [176,197,198,199].

State-dependent neuromodulation shapes these thalamocortical effects. The locus coeruleus–norepinephrine (LC–NE) system tunes thalamic feature selectivity and thalamo–cortical synchronization; arousal manipulations alter the relative thalamic versus cortical contributions to emergent network dynamics. In a soothing, predictable tactile context, reduced LC–NE drive would be expected to relax sensory gating and favor DMN–salience rebalancing through thalamic hubs [200,201,202,203].

The mechanistic implication of massage is that slow, pleasant touch signals safety. It shifts arousal, allowing pulvinar and MD hubs to retune cortico–cortical communication—down-weighting externally oriented, attentionally driven coupling while facilitating DMN-compatible, interoceptive regulation mediated via salience pathways. This hub-level reconfiguration offers a principled account of network changes observed after calming tactile input without invoking massage-specific imaging data.

#### 4.2.9. Specificity vs. Non-Specificity: Expectation, Context, and Social Modulation

A large share of the analgesic and calming benefits attributed to manual touch can be explained by nonspecific mechanisms—expectations formed in context, learning (conditioning/observation), and the social milieu—each with well-characterized neural and behavioral effects. Placebo-analgesia work shows that expectancy alone recruits endogenous control and reduces pain reports; critically, the open–hidden paradigm demonstrates that identical pharmacologic inputs are far more effective when delivered openly with patient awareness (i.e., rich context) than covertly (minimal context), isolating the psychosocial contribution to analgesia [204].

At the neural level, expectancy-driven relief engages prefrontal–cingulate control and the descending pain-modulatory system, with naloxone reversing both behavioral analgesia and rACC/PAG responses—direct evidence that belief-mediated relief taps opioid-sensitive circuitry. Meta-analytic and mechanistic syntheses converge on small but widespread reductions in pain-network activity (insula/somatomotor) with frontoparietal increases under placebo, consistent with context-dependent top-down control [181,205].

Learning strongly shapes these nonspecific effects. Classical conditioning paired with verbal suggestion produces robust placebo hypoalgesia and can even reverse nocebo responses; order and congruence of learning signals matter, and conditioned placebo effects can persist when explicit expectations fade. Observational learning—merely seeing others experience relief—induces comparable analgesia, highlighting the powerful social transmission of therapeutic expectations. Operant reinforcement (social/token rewards for lower pain reports) can also establish placebo hypoalgesia [206,207,208,209,210].

The social context surrounding care is a potent amplifier. In a randomized IBS trial using sham acupuncture, an augmented patient–practitioner relationship (warm, empathic, confident) produced substantially greater symptom relief than a limited interaction, despite identical inert procedures—implicating relational factors as “dose-escalators” of placebo response. Subsequent analyses showed that clinician behavior and patient traits (e.g., extraversion) modulated outcomes, and later experiments confirmed that provider warmth and competence independently boost placebo effects and can attenuate nocebo responses [211,212,213,214].

Social buffering during threat further illustrates tactile context effects: handholding by a trusted partner dampens right anterior insula and hypothalamic responses during shock threat, with relationship quality scaling neural attenuation—evidence that affiliative contact down-regulates salience and arousal [160]. In real clinical dyads, brain-to-brain concordance and nonverbal mirroring between patient and clinician predict greater analgesia, underscoring that interpersonal synchrony is not incidental but mechanistically relevant [215].

Finally, contemporary frameworks integrate these strands: placebo effects arise from predictive and associative processes centered on vmPFC/rACC that generate safety/relief predictions and engage descending control; reviews span pain, autonomic, and immune endpoints, making clear that context and social signals are biologically active ingredients rather than confounds [188,216,217].

The implication for massage is that because expectations, learned associations, and supportive social cues can independently produce analgesia and relaxation, well-controlled studies often find active vs. sham touch differences to be minor in healthy cohorts; the perceived benefits of massage are therefore plausibly dominated by these nonspecific (contextual and social) mechanisms layered on top of generic tactile input.

### 4.3. fNIRS-Based Massage Mechanisms

Functional near-infrared spectroscopy (fNIRS) indexes change in cortical oxy-/deoxy-hemoglobin linked to neurovascular coupling; increases in HbO and decreases in HbR are a canonical signature of local neuronal engagement [218]. Drawing on broad fNIRS and neuroimaging evidence, the hemodynamic patterns reported from massage are consistent with a set of converging mechanisms.

#### 4.3.1. Autonomic Pathways: Vagal Tone, HRV, and Prefrontal Control

Gentle touch and relaxing odors increase parasympathetic activity (higher HF-HRV), which tracks with reduced heart rate/blood pressure. They are embedded in the neurovisceral integration model, where prefrontal networks regulate vagal output. SCR/subjective arousal measures sit on this axis. Because autonomic state powerfully modulates cerebral blood flow (via CO_2_ and vasoactive routes), the global HbO/HbR shifts during relaxing massage likely include a parasympathetic component alongside local neurovascular coupling [219,220].

#### 4.3.2. Aromas During Foot Care: Olfactory–Reward Contributions

Aromatherapy engages olfactory–limbic–OFC networks and shifts autonomic balance (HRV). Lavender/sandalwood (and blends) often raise parasympathetic indices and alter prefrontal/limbic activity; pleasant odors also change OFC functional connectivity. Thus, the larger, early prefrontal HbO excursions you described during aroma-oil massage are expected from converging tactile and olfactory hedonic input [221,222].

#### 4.3.3. Pain Relief and Motor Circuits: Descending Control and Lateralization

Analgesic effects of supportive touch/massage align with classic gate-control ideas and, more importantly, with engagement of descending pain-modulatory systems (PAG→RVM→spinal dorsal horn). Attention, expectation, and social context recruit these top-down circuits, which can reduce S1 pain responses while increasing activity in medial prefrontal/brainstem hubs. In motor impairment (e.g., stroke), tactile stimulation at Hegu can bias activity toward contralateral SM1 and premotor areas when pathways are intact. Still, lateralization is blunted when circuits are disrupted—matching lateralization index results [223,224].

### 4.4. Integrative Neuroimaging Synthesis: How EEG, fMRI, and fNIRS Jointly Describe Massage Effects on the Brain

A primary reason that “integration” can feel incomplete in this literature is that the three methods do not measure the same thing: EEG indexes fast electrical population dynamics (rhythms, phase synchrony/coherence, complexity), whereas fMRI and fNIRS index slower neurovascular responses (BOLD or oxy-/deoxy-hemoglobin) that reflect synaptic/metabolic demand and vascular control. A proper integration, therefore, is not to force one-to-one matches between “alpha” and “BOLD,” but to map (i) sensory input → (ii) central network engagement → (iii) autonomic–neurovascular consequences, and then show how each modality captures a different segment of that causal chain.

#### 4.4.1. A Shared Mechanistic Backbone Across Modalities

Massage can be conceptualized as a “bottom-up” neuromodulatory stimulus that enters the brain through several converging afferent channels and then reorganizes large-scale neural processing in ways that each neuroimaging modality captures at a different level of the same cascade. Mechanistically, tactile stimulation of skin and deeper tissue activates fast-conducting Aβ mechanoreceptors (discriminative touch), proprioceptive and muscle/joint afferents (pressure, stretch, and movement), and—when touch is gentle, stroking, and socially delivered—C-tactile-related pathways that carry affective qualities of touch. These peripheral inputs ascend via spinal and brainstem relays to thalamic nuclei and brainstem autonomic centers while simultaneously engaging cortical sensorimotor representations. The immediate cortical consequence is a reweighting of sensory gain and network coordination: sensory-discriminative information is represented in S1 and related sensorimotor regions, while the interoceptive meaning of the bodily state is integrated in the insula and cingulate areas that also participate in autonomic control. Over seconds to minutes, this produces a broader shift in intrinsic network organization—particularly within default-mode and salience circuits—that supports changes in self-referential processing, emotional tone, and pain regulation. EEG, fMRI, and fNIRS index different segments of this same process: EEG resolves the rapid electrical signatures of changing excitability and coupling; fMRI localizes which network nodes and pathways show altered BOLD activity or functional connectivity; and fNIRS tracks cortical hemodynamic engagement in a way that is feasible during more naturalistic protocols and in populations less suitable for MRI.

Within this unified cascade, a first core route is the somatosensory–sensorimotor pathway. Massage reliably drives somatotopic processing of the stimulated body region, and this is most directly captured by fMRI evidence that focal foot or toe stimulation activates S1 representations consistent with known somatosensory organization and can recruit sensorimotor areas beyond the immediate foot map in reflexology paradigms. Complementing this, fNIRS studies that place optodes over S1 and adjacent regions show oxygenation changes time-locked to massage blocks, indicating that tactile input produces measurable cortical hemodynamic responses even in elderly participants or clinical populations (e.g., stroke rehabilitation paradigms). EEG captures the same pathway in its fastest form: unilateral massage produces localized contralateral changes in rhythm power consistent with altered excitability in the somatosensory cortex, and point stimulation or mechanical manipulation modulates coherence and synchronization between scalp regions that plausibly reflect sensorimotor network coordination. Importantly, these sensory-route effects can be either “stabilizing” (greater coupling during quiet rest) or “flexibilizing” (reduced coupling when sensory processing demands increase), which helps explain why some studies observe increased synchrony after massage in eyes-closed conditions while others report reduced coherence or desynchronization under eyes-open or stimulation-heavy conditions.

A second core route is the interoceptive salience pathway linking bodily signals to autonomic and affective regulation. Massage changes not only what the brain feels on the skin but also what the brain infers about the internal state of the body (safety, calm, pain, fatigue). This inference is anchored in cingulate–insular circuitry and related thalamic and brainstem hubs that integrate visceral state, pain, and arousal. fMRI work in manual therapy and tuina shows modulation in regions commonly treated as part of a pain/interoception network—insula, anterior and posterior cingulate cortices, thalamus, and connectivity with descending modulatory nodes such as the PAG—consistent with a central mechanism for analgesia and autonomic recalibration. EEG findings map neatly onto this route when interpreted as state markers: increases in alpha and/or delta power after massage in stressed individuals, shifts in frontal asymmetry toward greater left-sided activation (often associated with more positive affect), and reductions in signal complexity after massage in pain populations all fit with a brain moving toward lower threat load, lower interoceptive “error,” and reduced need for sustained pain monitoring. fNIRS adds translational leverage here by capturing prefrontal and sensorimotor oxygenation changes during interventions used in rehabilitation or fatigue reduction, suggesting that autonomic settling and cognitive readiness can be tracked as hemodynamic signatures in cortical control regions even outside the scanner.

A third core route is the default-mode and valuation/social-touch pathway, which explains why “massage” effects are not fully accounted for by somatosensory stimulation alone. The meaning of touch—human vs. mechanical, pleasant vs. neutral, socially affiliative vs. purely physical—recruits networks that evaluate reward, social relevance, and self-referential processing. fMRI evidence shows that Swedish massage modulates key default-mode hubs (subgenual ACC and posterior cingulate/retrosplenial cortex) during rest, supporting the idea that massage can bias the brain toward internally oriented, emotion-regulatory modes that overlap with DMN function. Separate fMRI work on pleasant touch shows that moving, skin-to-skin stimulation engages insula and somatosensory regions but uniquely recruits pregenual ACC when the touch is both human and dynamic—linking hedonic touch to reward/affective circuitry. fNIRS provides particularly direct leverage for integrating this route with peripheral physiology: hand-administered massage produces larger oxytocin increases than machine massage and preferentially activates posterior STS and orbitofrontal regions associated with social cognition and reward valuation, even when primary somatosensory activation does not differ. This dissociation is crucial mechanistically because it implies that a substantial component of massage-related brain change reflects higher-order valuation and affiliative processing layered on top of basic tactile input. EEG does not localize OFC or STS reliably. Still, it can register downstream consequences of this valuation shift as changes in frontal rhythms, coherence, and asymmetry—effects that vary with pressure, therapist vs. device delivery, and participant state.

#### 4.4.2. Linking EEG Rhythms/Connectivity to fMRI/fNIRS Networks: Convergence and “Apparent Contradictions”

EEG rhythms and connectivity metrics often look “inconsistent” across massage studies until they are interpreted at the level of network function rather than as fixed markers with one meaning. The central integration point is that EEG power, coherence, asymmetry, and entropy are fast electrical readouts of shifts in cortical excitability and communication. In contrast, fMRI and fNIRS describe where and how strongly slower neurovascular signals change in distributed networks. When the techniques are aligned by function—sensorimotor processing, salience/interoception, default-mode/self-referential activity, reward valuation, and pain modulation—the same massage manipulation can reasonably yield different-looking EEG signatures that nevertheless converge with fMRI/fNIRS findings about which networks are being biased and why.

A first convergence is that massage reliably alters the balance between internally oriented resting networks and externally oriented sensory/attention processing, and EEG “alpha” is especially sensitive to this balance. In some datasets, massage increases alpha power or alpha synchrony during quiet rest, particularly in stressed individuals or after moderate-pressure/manual techniques, consistent with a shift toward calmer internal regulation. This aligns with fMRI evidence that massage can modulate default-mode hubs such as the subgenual ACC and posterior cingulate/retrosplenial cortex during rest, regions typically linked to self-referential processing and affect regulation. However, other studies show alpha attenuation after massage—most clearly in protocols where participants are not simply “resting” in a passive way but may be transitioning into a state of reduced cognitive elaboration or altered vigilance. Alpha decreases are not a contradiction of relaxation; they can reflect cortical desynchronization accompanying efficient processing, sensory readiness, or reduced “idling” in specific regions. This is precisely where fMRI/fNIRS are clarifying: if neurovascular responses indicate increased engagement of valuation or salience circuitry (e.g., OFC/STS responses during socially meaningful human touch), an EEG pattern with reduced alpha and/or beta power can reflect a different relaxation endpoint—calm but engaged—rather than a sleep-like state. In other words, “relaxation” is not a single electrophysiological destination: massage can push the brain toward a low-demand default-mode-dominated condition (often alpha-synchronized) or toward a regulated, attentive readiness state (often more desynchronized), and both can be accompanied by decreased heart rate or improved mood.

A second convergence concerns connectivity. EEG coherence/cross-correlation measures capture frequency-specific coupling and are best understood as fast indicators of how strongly networks are functionally “bound” at a given moment. fMRI resting-state connectivity captures slower co-fluctuations across regions and is often interpreted as large-scale network integration or segregation. Across the massage literature, the two can be integrated through a single principle: massage can promote “adaptive reconfiguration,” increasing coupling where stability is beneficial and decreasing coupling where flexibility or sensory responsiveness is beneficial. For example, increased fronto-central synchrony during eyes-closed rest can be interpreted as strengthened coordination within internally oriented regulatory circuits, whereas decreased coupling between central and occipital regions during eyes-open conditions can be interpreted as reduced redundant linkage, allowing more responsive sensory processing. This couple/decouple logic mirrors fMRI network findings where post-massage states sometimes show reduced integration or clustering in hubs implicated in affective monitoring or attentional gating, consistent with a lowered need for vigilance. Thus, an EEG decrease in coherence after massage is not necessarily “worse”; it can reflect network efficiency and reduced over-coupling—an interpretation that becomes more compelling when fMRI/fNIRS show concurrent shifts in networks associated with salience and attention control.

A third convergence is in the domain of affect and emotion, where EEG frontal asymmetry offers a functional bridge to cingulate–insular and medial prefrontal circuitry emphasized by fMRI and fNIRS. Several massage protocols report shifts toward greater left-frontal activation (often inferred from alpha asymmetry) in stressed or affectively burdened participants, a pattern commonly associated with more positive affect and approach-related motivation. fMRI and fNIRS studies provide convergent anatomical grounding for this phenomenon: pleasant/social touch recruits pregenual and subgenual ACC and insula, and human-delivered massage preferentially engages OFC and STS—regions that support affective valuation and social meaning. The integration is that frontal EEG asymmetry is likely a surface-level electrical correlate of deeper network biasing toward reward/affiliative and emotion-regulatory systems. Where studies fail to find significant asymmetry changes, this can also be reconciled: if the massage context is brief, mechanically delivered, or primarily sensorimotor without strong affective meaning, fMRI/fNIRS suggests that S1 engagement may occur without robust recruitment of higher-order valuation circuitry, making a null asymmetry result plausible even when participants report feeling relaxed.

The strongest multi-technique convergence appears in pain-related studies when EEG complexity/entropy measures are linked to fMRI pain-network modulation. In chronic or acute musculoskeletal pain contexts, multiple EEG studies show reductions in signal complexity (sample entropy, permutation entropy, approximate entropy) after massage, sometimes accompanied by shifts toward higher energy in slower rhythms. These results are often interpreted as reduced cortical “strain,” decreased nociceptive salience, or stabilization of neural processing after analgesia. Resting-state fMRI studies of tuina/manual therapy in pain conditions converge on modulation of regions that define a pain-processing and pain-modulatory architecture: insula and cingulate for affective–interoceptive components of pain, thalamus for gating of ascending input, medial prefrontal/DMN hubs for self-referential pain rumination, and PAG-related connectivity for descending modulation. The integrated claim is that massage-induced analgesia involves both altered afferent inflow and a central retuning of salience and descending control; EEG entropy reductions are the electrical signature of a system requiring fewer variable “solutions” to manage pain, while fMRI identifies the anatomical network components through which that stabilization is likely implemented. Importantly, some studies observe different entropy directions in healthy controls versus pain patients, which can be interpreted as baseline-dependent: a healthy brain may show small complexity increases reflecting sensory enrichment without threat, whereas a brain in pain may show complexity decreases reflecting reduced nociceptive load and arousal.

fNIRS further helps resolve apparent EEG contradictions by separating sensory coding from social/hedonic valuation and by capturing time-varying hemodynamic trajectories during stimulation. When fNIRS shows that S1 does not distinguish human vs. machine massage but OFC/STS does, it becomes plausible that two protocols with similar tactile input may diverge in EEG outcomes depending on whether the touch is interpreted as socially meaningful and rewarding. Likewise, aroma-oil massage fNIRS trajectories that include an early rise in oxygenation followed by a decline can map onto EEG findings where high-frequency activity may transiently increase (orienting/novelty) and then decrease (settling/relaxation), or where alpha may briefly attenuate and then rebound, depending on when and how EEG is sampled. This helps explain why short post-only EEG epochs sometimes capture “activation-like” signatures and other times capture “sedation-like” signatures: the brain may be traversing a standard trajectory (salience response → autonomic settling), and modalities with different temporal sensitivities or sampling windows will “see” different segments.

#### 4.4.3. Where fNIRS Adds Unique Integrative Value (Beyond “Cortical Oxygenation”)

fNIRS contributes an integrative value that goes well beyond the general statement that it measures cortical oxygenation. In this literature, its distinctive role is that it can (i) isolate which cortical systems are being recruited during massage in ways that help interpret ambiguous EEG signatures, (ii) characterize within-session trajectories of engagement versus settling that are difficult to capture with pre-/post-only designs, and (iii) provide mechanistic biomarkers in populations and settings where fMRI is impractical and EEG can be challenging or insufficiently specific. Because fNIRS sits between EEG and fMRI in spatiotemporal sensitivity—more spatially informative than EEG for superficial cortex and more temporally feasible than fMRI for repeated or naturalistic protocols—it is particularly suited for connecting “fast electrical state shifts” to “network-level hemodynamic engagement” in ecologically relevant massage paradigms.

A first unique contribution is fNIRS’ ability to disentangle basic tactile coding from the social–hedonic and cognitive meaning of touch. EEG often shows that massage changes arousal-related rhythms and coupling, but these electrical changes do not reveal whether the driver is sensorimotor afference, affective valuation, expectancy, or social context. fNIRS studies directly demonstrate that the presence of human touch can selectively engage higher-order cortical systems even when the physical stimulation is matched. In the hand vs. machine-administered foot massage paradigm, primary somatosensory cortex (S1) responses did not differentiate conditions, whereas posterior superior temporal sulcus (pSTS) and orbitofrontal cortex (OFC) did, and these cortical differences aligned with larger endogenous oxytocin increases and greater subjective pleasure for human touch. This dissociation is integratively powerful: it provides a cortical mechanism for why two interventions can yield similar “relaxation” self-reports yet different EEG outcomes, and it clarifies why mechanical massage sometimes produces EEG patterns suggestive of arousal or network disruption. The fNIRS evidence supports the idea that some massage effects are not driven by tactile intensity per se but by recruitment of social-cognition and reward-valuation circuitry layered on top of a relatively constant sensorimotor input stream.

A second contribution is that fNIRS captures the temporal evolution of cortical engagement during massage blocks, which helps reconcile the timing-dependent variability seen in EEG studies. Massage is not a static stimulus; it often elicits a rapid orienting response (novelty, salience, sensory updating) followed by a slower settling phase (autonomic downshift, reduced monitoring demands). fNIRS block designs can reveal this trajectory as an early increase in oxyhemoglobin with subsequent decline or stabilization across prefrontal and somatosensory channels, including patterns suggesting strong initial engagement followed by calming effects in aroma-oil massage protocols. This time-resolved hemodynamic profile provides a principled way to interpret why EEG sometimes shows alpha attenuation or beta increases (if measured during an early engagement period) and other times shows increased alpha synchrony or reduced high-frequency power (if measured during later settling). Rather than treating these EEG outcomes as contradictory, fNIRS supports a trajectory model in which different sampling windows or task contexts emphasize various phases of the same underlying response.

A third contribution is translational and population-specific: fNIRS enables mechanistic monitoring during massage in groups where fMRI is difficult and where EEG alone may not provide a targeted biomarker. In stroke rehabilitation paradigms using Tui Na at Hegu, fNIRS quantifies lateralized sensorimotor cortex oxygenation and shows that affected limbs display disrupted contralateral dominance relative to less-affected limbs and healthy controls. This offers a physiologically interpretable marker of sensorimotor circuit integrity and reorganization that can be measured during the intervention itself, which is especially valuable when voluntary movement tasks are limited. In infant studies, fNIRS can measure motor cortex oxygenation responses to massage in a home environment with minimal burden, and it can differentiate response patterns to massage versus other tactile therapies, providing a window into early-life neuroplasticity and state regulation. These applications complement neonatal EEG maturation studies by adding a task-locked cortical hemodynamic dimension—practical when the central question is not only whether global electrophysiological maturation changes but also how specific cortical regions respond dynamically to tactile stimulation.

A fourth contribution is that fNIRS strengthens multi-level mechanistic chains by linking cortical responses to peripheral physiology and subjective valuation in the same experimental session. Several fNIRS paradigms explicitly combine cortical oxygenation measures with endocrine (oxytocin), autonomic (skin conductance), and subjective ratings (pleasure, willingness to pay, relaxation), allowing mediation-style interpretations that are difficult with EEG alone. For example, evidence that pSTS and OFC activation tracks pleasure and valuation, while S1 does not differentiate human versus machine touch, supports a pathway in which sensory input is processed through social-perceptual regions before influencing reward evaluation. This kind of integrative linkage is precisely what is often missing in single-modality studies: fNIRS can serve as the cortical “middle layer” connecting peripheral biomarkers and subjective experience to network-level interpretations that are otherwise inferred indirectly.

## 5. Neuroplasticity and Longitudinal Effects of Massage Therapy

### 5.1. Neuroplasticity in the Context of Manual Therapies

Neuroplasticity refers to the nervous system’s capacity to reorganize its structure and function in response to experience. In manual therapies like massage, neuroplasticity implies that repeated therapeutic touch can lead to lasting changes in brain activity, connectivity, and even the molecular milieu of the nervous system. Massage provides a rich sensory and mechanotransductive stimulus—activating skin and muscle mechanoreceptors, modulating peripheral inflammation, and engaging afferent pathways linked to discriminative touch (Aβ fibers) and affective touch (C-tactile fibers). These inputs do more than produce transient feelings of relaxation or pain relief; they can drive adaptive changes in neural circuits over time. For example, preclinical studies suggest that massage not only aids peripheral tissue healing but may also induce central changes: one animal study found that massage therapy after nerve injury altered gene expression (DNA hydroxymethylation) related to neurodevelopment, hinting at deep biological plasticity. In humans, massage consistently triggers parasympathetic activity (vagal tone increase, cortisol reduction) and releases neuropeptides like oxytocin, which can shape brain network dynamics associated with stress and social bonding. Repeated engagement of these pathways through regular massage could plausibly recalibrate the brain’s stress response and social-emotional processing systems in a lasting way. Conceptually, we can view massage as a form of somatosensory training or enrichment: just as repeated practice of a motor skill induces cortical remapping, repeated therapeutic touch might reinforce circuits that underlie interoception, pain modulation, and emotional regulation. Over time, this may translate into more enduring changes in how the brain processes bodily information and maintains homeostasis.

### 5.2. Immediate vs. Long-Term Neural Responses to Massage

Neuroimaging research shows that a single massage session produces apparent acute effects on the brain, but these immediate changes are often transient. For instance, many EEG studies have documented shifts in brain rhythms during and shortly after massage—commonly an increase in alpha (8–13 Hz) and a concomitant decrease in beta (13–30 Hz) and gamma (>30 Hz) power, reflecting a relaxed-alert state. Such spectral changes, associated with reduced arousal and anxiety, typically emerge within minutes of massage onset and dissipate within minutes to hours after the session. Similarly, fMRI studies show acute modulation of functional connectivity and regional activation during or immediately following massage-like touch. For example, after a brief calf massage, within-session fMRI revealed decreased connectivity in a subnetwork of regions (including hubs in the thalamus and anterior cingulate) compared to a rest-only control, consistent with a temporary downshift in integrative processing load. However, without continued stimulation, the brain’s activity tends to return to baseline patterns. In other words, a single dose of massage yields short-term neural effects—engaging reward circuits, diminishing salience network activity, and enhancing default-mode network (DMN) connectivity in some cases—but these effects are usually not permanently imprinted after one session.

Crucially, the neural effects of massage accumulate and adapt with repeated sessions. Two phenomena have been observed with ongoing exposure (e.g., regular treatments over weeks): habituation of immediate responses and gradual development of new baseline states. An illustrative example is an EEG study of aromatherapy massage administered twice weekly for 4 weeks. Early in the intervention, each session evoked robust alpha increases and delta (0.5–4 Hz) decreases relative to the pre-massage baseline; by the 8th session, the same immediate EEG shift had diminished, suggesting the brain had partially adapted to the familiar stimulus. Notably, participants’ pre-session resting EEG did not significantly change over the month—indicating no significant spontaneous shift in baseline oscillatory power—yet a neurotrophic change was detected: brain-derived neurotrophic factor (BDNF) levels rose significantly over the 4 weeks. In parallel, salivary cortisol responses to massage attenuated (initial sessions showed cortisol reductions that were no longer present by the final session) even though the overall clinical state improved. This pattern—diminishing acute physiological responses but strengthening neurotrophic milieu—implies that repeated massage engages homeostatic plasticity. The body becomes less reactive to each session (perhaps a sign of habituation or tolerance). At the same time, longer-term adaptations (like increased growth factors or receptor-level changes) accrue beneath the surface.

Another study in office workers who received chair massage twice weekly for 5 weeks echoed this dynamic. Early sessions induced pronounced EEG changes (e.g., frontal delta increases with alpha/beta decreases corresponding to relaxation) and cortisol drops immediately after massage, but by week 5, these acute changes were blunted. Despite the reduced momentary responses, participants’ baseline stress levels and job-related anxiety steadily declined over the intervention period. In other words, behavioral and psychological improvements accumulated even as the acute EEG/cortisol effects became less marked, suggesting that the brain and body had adjusted to the repeated stimulus in a way that conferred lasting benefit. Such findings highlight the difference in time scales: immediate neurophysiological effects (brain rhythms, heart rate, stress hormone release) vs. longitudinal outcomes (chronic stress reduction, enhanced well-being), and they underscore that short-term and long-term effects may not simply be the same changes prolonged but can involve distinct processes (e.g., fast neural feedback loops versus slower neuroplastic restructuring).

In summary, the acute impact of massage on the brain is real but ephemeral—a rapid state change. Sustained massage therapy practice engages the brain’s plasticity mechanisms, leading to adaptation (less dramatic state shifts with familiarity) and potentially laying the groundwork for enduring functional changes (like improved stress resilience or pain regulation). Increased BDNF after weeks of massage is particularly intriguing, as BDNF is a key mediator of synaptic plasticity and learning. These findings hint that repeated massage may stimulate neurochemical pathways that support long-term potentiation of specific neural circuits, even if outward EEG signals become subtler with repetition. Additionally, the distinction between transient and lasting effects reinforces the importance of longitudinal studies—a single-session experiment might miss the neuroplastic changes that only manifest with ongoing treatment.

### 5.3. Neuroplastic Changes Evidenced by Longitudinal Massage Studies

#### 5.3.1. Longitudinal Brain Changes in Clinical Populations

The most unmistakable evidence that massage can induce neuroplastic reorganization comes from longitudinal studies in clinical populations with aberrant baseline brain function (such as chronic pain or neurological disorders). In such cases, researchers have observed that massage therapy alleviates symptoms and shifts patients’ brain activity/connectivity toward a healthier state. For example, recent fMRI investigations in chronic pain conditions have shown normalization of dysfunctional networks after a multi-session massage-based intervention. In one study, patients with lumbar disc herniation at baseline showed abnormally low regional homogeneity (ReHo) in the medial prefrontal cortex (a core node of the default-mode network) who underwent a series of tuina massage treatments. After therapy, ReHo in that prefrontal/DMN region increased to near-normal levels, paralleling clinical improvement. A similar trial in patients with chronic cervical spondylosis noted that aberrant hyperactivity in the anterior cingulate and posterior cingulate cortices (regions implicated in pain processing and default-mode function) significantly shifted after repeated tuina sessions, in conjunction with reduced neck pain and disability. These studies directly demonstrate neuroplasticity: the patients’ brains changed in activity patterns over treatment, not just during an isolated session. Converging evidence comes from a recent synthesis of multi-session neuroimaging studies, which concluded that repeated manual therapies like tuina and osteopathic manipulation tend to normalize both local activity (ReHo) and functional connectivity (FC) in key networks (DMN, salience/interoceptive networks), together with clinical symptom improvements. In other words, as patients feel better over weeks of therapy, their brains are measurably reorganizing—for instance, showing stronger resting-state connectivity within pain-modulatory circuits and more balanced activity in default-mode versus salience network hubs.

Another domain where longitudinal massage effects have been studied is manual therapy for musculoskeletal pain and mobility issues. An fMRI trial of osteopathic manipulative treatment (OMT) in chronic low back pain used a pre/post design with multiple time points (baseline, after one session, and one month after a series of sessions). It found time-specific changes in insular and cingulate responses during interoceptive tasks, suggesting that even one month after the intervention, specific brain changes persisted in the OMT group compared to sham therapy. Although more research is needed to confirm long-term retention, this hints that some massage-induced neural adaptations (particularly those related to internal body-awareness pathways) can last beyond the immediate treatment window. EEG studies in chronic pain patients also reflect longitudinal changes: for example, patients with low back pain or sciatica who underwent repeated massage exhibit systematic shifts in EEG markers of cortical excitability and information flow (such as decreases in frontal beta power and EEG entropy corresponding to pain relief) that distinguish their post-treatment brain state from pretreatment and from healthy controls. These changes suggest a partial “rewiring” or recalibration of pain-related neural networks—a reversal of the heightened cortical arousal and disorganization that often accompanies chronic pain. Notably, advanced machine learning classifiers have accurately identified patients’ post-massage EEG patterns, implying a consistent therapeutic signature in the brain.

Emerging evidence also points to massage promoting neuroplastic changes in conditions like stroke and other neurologic impairments. While much of massage therapy in rehabilitation is aimed at peripheral tissues and improving circulation or spasticity, neuroimaging reveals central effects. A fNIRS study in stroke survivors examined repeated sessions of acupressure massage (tui na) at an acupoint (Hegu) on the impaired arm. Over the course of treatment, patients demonstrated enhanced activation in the contralesional (uninjured hemisphere) sensorimotor cortex during stimulation, indicating that massage helped engage residual cortical representation of the weakened limb. Patients with more intact corticospinal pathways showed greater lateralized activation (contralateral to the side of massage), whereas those with severe damage showed blunted lateralization. This pattern suggests that massage can facilitate use-dependent plasticity in motor networks—effectively “reminding” the brain to activate motor areas linked to the affected limb, which is crucial for recovery. Although the effect was immediate during each session, the fact that it was observed repeatedly and more strongly in those capable of recovery hints that repeated massage could strengthen these cortical pathways over time, aiding motor relearning. A systematic review reported that massage therapy added to standard rehab improved motor function in post-stroke patients, an outcome likely underpinned by neuroplastic changes in both spino-supraspinal circuits.

Massage’s capacity to modulate brain chemistry over the long term is another facet of neuroplasticity. We have mentioned the rise in BDNF with weeks of massage; BDNF is known to facilitate synaptic growth and dendritic branching, so its elevation could promote structural plasticity (e.g., strengthening of synapses in cortical or hippocampal regions) [225,226,227,228]. Additionally, repeated massage might recalibrate neuromodulator systems. For example, whereas a single massage robustly lowers cortisol and increases oxytocin, chronic exposure may lead to a new equilibrium where baseline cortisol output is reduced (indicating a chronically less reactive HPA axis), and oxytocin responsiveness is enhanced. Though direct longitudinal studies on neurotransmitters are sparse, one fMRI experiment demonstrated that exogenous oxytocin selectively augmented the brain’s response to massage—amplifying activation in reward and social cognition regions during massage touch. This finding implies that the oxytocin system is a lever for massage effects; by extension, regular massage might naturally upregulate oxytocin pathways over time, reinforcing the positive feedback loop between social touch and neural reward circuits. Indeed, a near-infrared spectroscopy study found that foot massage triggers oxytocin release alongside increased prefrontal cortex activity in healthy adults. Repeated stimulation of this response could conceivably lead to lasting increases in social affiliative behavior and stress resilience—training the brain to release oxytocin more readily in response to touch, thereby dampening stress circuits in the long run.

#### 5.3.2. Developmental and Lifespan Considerations

The developing brain may be especially malleable to the effects of therapeutic touch. Research on infant massage provides some of the most compelling evidence that massage can induce long-term changes in brain function. Preterm infants in neonatal intensive care who receive regular massage tend to show accelerated neurological maturation compared to non-massaged controls. In one randomized trial, very preterm infants were given moderate-pressure massage twice daily from 34 weeks postmenstrual age until term-equivalent age. At the end of this intervention, the massaged infants had significantly higher EEG power in beta and alpha bands (and lower delta) than non-massaged infants, indicating a more advanced brain electrical profile for their age. Notably, the typical developmental decline in delta power and increase in faster rhythms that occurs as infants approach term [229,230,231,232] was enhanced by massage. Essentially, their brains appeared “older” or more developed in activity pattern, which is generally a positive sign for neurodevelopment. Another study found that preterm newborns who received massage three times a day for 10 days did not exhibit the usual postnatal drop in global EEG power seen in unstimulated infants; instead, they maintained higher levels of delta and theta power over a 2–3 week period, with significant time × group interactions in EEG trajectories. Moreover, amplitude-integrated EEG (aEEG) monitoring in very low birthweight infants showed that those receiving massage had more continuous brain wave patterns and fewer immature waveform bursts during a 12 h recording, compared to controls. Continuous EEG background and higher-frequency activity are markers of brain maturation, suggesting that massage fostered more mature sleep–wake cycling and cortical synaptic development in these fragile infants.

The long-term significance of these findings is underscored by known links between early brain electrical maturation and later cognitive outcomes. While follow-up studies are needed, it is plausible that massage in the NICU contributes to better neurodevelopmental outcomes (as hinted by separate behavioral studies where massaged preterm infants gain weight faster and show superior motor development). At a minimum, these neuroimaging results confirm that the infant brain is highly plastic and responds to repeated tactile stimulation by altering its functional activity patterns in a lasting way, beyond the massage sessions themselves.

Massage can induce neuroplastic changes related to emotional regulation even in full-term infants or older children. A notable example: in one study, one-month-old infants of depressed mothers—who often exhibit a biologically driven right-frontal EEG asymmetry associated with distress or withdrawal—were given a brief massage therapy intervention. After the massage, these infants significantly attenuated right-frontal asymmetry, essentially a shift toward a more balanced (or leftward) frontal activity pattern. This is important because a relative excess of right-frontal activity in infants has been linked to negative affect and later risk of socioemotional problems. By normalizing the asymmetry, massage might help guide the developing brain toward a healthier affective bias. While this study examined an acute effect, the fact that a single massage could alter an infant’s EEG pattern highlights how sensitive the infant brain is to social touch—repeated massage could conceivably have cumulative benefits for infants at risk (e.g., those born to depressed or anxious mothers or infants with developmental delays).

At the other end of the lifespan, there is growing interest in how massage might support the aging brain. Changes in neuroplastic capacity accompany aging [233] and are often associated with dysregulation of autonomic function [234,235] and sleep [236,237,238], which touch therapies might influence. A pilot EEG study in older adults (both healthy and those requiring nursing care) evaluated massage with and without aromatherapy and found that all forms of the intervention produced measurable EEG and physiological changes, such as increases in alpha power and reductions in beta power post-treatment, in both senior and younger adult groups. These immediate changes correspond to relaxation and improved arousal regulation. Although long-term effects in the elderly have not been as extensively studied, some clinical trials suggest benefits of regular massage for dementia patients (e.g., reducing behavioral symptoms) [239] and for older adults’ mood [240] and sleep [241]. It is reasonable to hypothesize that repeated massage in older individuals could help maintain functional connectivity in networks vulnerable to aging (such as frontal lobe networks for executive function or limbic networks for mood) by providing ongoing sensory and social stimulation. The neuroplastic potential certainly exists even in later life—the challenge is that it may require more frequent or intensive intervention to harness due to slower neural turnover. This remains an open area for research, but the acute results in older adults are promising and align with the idea that human touch can engage the aging brain’s neuroplasticity to preserve or enhance function.

In summary, across the lifespan—from the neonatal period through adulthood to old age—there is evidence that massage can produce changes in brain function that outlast the immediate intervention. These range from developmental acceleration in infants to network normalization in chronic pain patients, all underlining that the brain is a dynamic organ responsive to the cumulative touch experience. Next, we consider what these findings mean for clinical practice and future research.

### 5.4. Clinical and Translational Implications

The demonstration of massage-induced neuroplasticity carries significant implications for clinical practice and wellness. Suppose massage can reshape brain activity and connectivity over time. In that case, it reinforces the role of manual therapy as not only symptomatic treatment but also a modulator of the central nervous system. Several domains stand to benefit.

#### 5.4.1. Rehabilitation and Motor Recovery

As seen in stroke patient studies, massage can facilitate motor circuit activation and potentially enhance functional recovery when used alongside conventional physiotherapy. Massage may strengthen residual neural pathways and promote cortical reorganization that supports motor relearning by repeatedly stimulating afferent inputs from muscles and skin. This suggests that incorporating massage or tactile stimulation in neurorehabilitation (for stroke, spinal cord injury, peripheral nerve injury) could improve outcomes by tapping into Hebbian plasticity—“neurons that fire together wire together.” For example, massaging a paretic limb while a patient attempts to move it might reinforce sensorimotor connections and increase the brain’s attention to that limb, accelerating the regain of motor control. Additionally, massage’s activation of descending pain inhibitory pathways (e.g., engaging the periaqueductal gray and rostroventral medulla) can reduce pain-related interference, allowing patients to participate more fully in rehabilitation exercises. Over the long term, this could translate to sustained improvements in mobility and a lower likelihood of central pain syndromes after injury.

#### 5.4.2. Chronic Pain and Pain Management

Chronic pain is increasingly understood as a condition of maladaptive neuroplasticity [242], where pain networks become hypersensitive [243,244] and the brain’s pain-modulatory systems are downregulated [245]. Massage appears to exert a corrective influence on these networks. Massage can “close the gate” at the spinal level and reduce activity in pain-related brain regions (like the primary somatosensory cortex S1 and insula) while boosting activity in medial prefrontal areas that read descending analgesia. With repetition, as the fMRI studies of tuina demonstrated, massage can normalize connectivity within the default-mode and salience networks that are often in chronic pain. Clinically, this means regular massage therapy might help reverse central sensitization—the brain’s tendency to over-amplify pain signals—by recalibrating the balance between pain-facilitating and pain-inhibiting pathways. Patients with fibromyalgia, low back pain, or osteoarthritis who receive ongoing massage often report not only immediate relief but also functional improvements and better sleep over weeks [246,247,248,249,250,251]; neuroplastic changes (like increased serotonin, decreased amygdala reactivity, or heightened frontal lobe inhibition of pain signals) likely underpin these subjective gains. These findings suggest that massage might serve as a valuable adjunct in pain management by potentially “training” the brain toward a less pain-sensitive state, which might reduce reliance on pharmacological interventions.

#### 5.4.3. Mental Health and Stress Regulation

The interactions between massage therapy and the brain’s stress and mood-regulating systems have important implications for treating anxiety, depression, and trauma-related conditions. Massage has been shown to acutely lower stress markers (e.g., cortisol, heart rate) in many studies and increases parasympathetic tone, which provides immediate anxiolytic and mood-elevating effects. Repeated massage might induce longer-term adjustments in the hypothalamic–pituitary–adrenal (HPA) axis and limbic system. For instance, the attenuation of cortisol responses after multiple sessions suggests the HPA axis becomes less reactive to stress triggers over time—a desirable adaptation in individuals with chronic stress or anxiety disorders. Concurrently, the consistent release of oxytocin during therapeutic touch can reinforce feelings of trust, safety, and social connectedness, directly counteracting the neurobiology of loneliness and hypervigilance that often accompanies depression or PTSD. Neuroimaging evidence of increased frontal lobe activity and a leftward frontal EEG asymmetry shift with massage aligns with an antidepressant-like neural effect, since frontal asymmetry is a known correlate of mood state [252,253,254,255]. Over a longer term, one could expect that regular massage, by repeatedly inducing calm, safe physiological states, might help rewire a person’s baseline mood regulation networks—somewhat analogous to the effects of meditation or biofeedback. In practice, integrating massage therapy into mental health care (for example, as part of treatment for PTSD or generalized anxiety) could leverage these neuroplastic effects to improve outcomes, as some clinical trials have begun to document (e.g., massage reducing trait anxiety and improving sleep quality when provided consistently) [256,257]. It also bears mentioning that the human touch aspect of massage addresses social neurobiology—many mental health conditions have elements of social isolation or touch deprivation [258,259], and massage fulfills a fundamental need for positive touch, thereby engaging brain circuits of attachment and reward that medications alone do not target.

#### 5.4.4. Neurodevelopment and Pediatrics

The use of massage in infants (both healthy and medically fragile) has clear translational significance. Given the evidence that massage accelerates EEG maturation and modulates developing sensory systems, routine massage could be a low-cost, low-risk intervention to support brain development in preterm infants. Hospitals worldwide are already employing infant massage care to promote growth [260,261,262]; the neuroimaging findings provide a mechanistic basis for those benefits and suggest that early tactile intervention might improve long-term neurodevelopmental outcomes (cognition, self-regulation, etc.). In typically developing children, massage therapy might aid in conditions like ADHD or autism spectrum disorder (where sensory processing and arousal regulation are atypical) by gradually teaching the child’s nervous system to tolerate and enjoy calm touch, potentially normalizing some sensory pathways. Indeed, a systematic review noted that massage therapy led to improvements in attention and behavior in children with ADHD [263], which could be related to improved frontal theta/beta ratios or other EEG changes indicative of a more regulated cortical state. While more research is needed in pediatric neuropsychiatric populations, the principle is that early-life interventions leveraging neuroplasticity may yield compounding benefits—and massage is a prime candidate, given its safety and developmental appropriateness.

#### 5.4.5. Wellness and Healthy Aging

Even outside of specific diagnoses, “use it or lose it” in brain aging suggests that stimulating the brain in novel ways can help maintain cognitive function. Massage provides a unique form of stimulation that combines physical, emotional, and social elements. Regular massage for healthy adults might bolster networks in body awareness and stress resilience, potentially buffering against age-related decline in those domains. For example, maintaining higher vagal activity through frequent relaxation responses could protect cardiovascular [264] and cognitive health [265]. Moreover, caregivers of patients with dementia have started using gentle massage to reduce agitation—this not only benefits the patient (possibly via activating remaining limbic touch pathways) but could also positively affect the caregiver’s brain by reducing stress and increasing oxytocin through the act of giving touch. Thus, the translational reach of massage-induced neuroplasticity might extend to improving quality of life in caregivers and health care providers, an area ripe for exploration.

### 5.5. Methodological Challenges and Future Research Directions

While the evidence for massage-related neuroplasticity is encouraging, studying these phenomena is complex. Researchers face several methodological challenges that future work must navigate.

#### 5.5.1. Diversity of Massage Intervention Protocols

Massage therapy is not a standardized treatment—it encompasses various techniques (Swedish, Thai, shiatsu, reflexology, myofascial release, etc.) applied to different body regions with varying pressure, duration, and context. This variability makes it challenging to generalize findings and compare studies. One solution is for future studies to report detailed dose parameters and, when possible, to systematically vary isolated elements (e.g., comparing effects of pressure levels or stroke frequency on neuroplastic outcomes) to identify which components are crucial for inducing brain changes. Developing standardized massage protocols for research will enhance reproducibility and allow dose–response relationships to be established for neuroplastic effects.

#### 5.5.2. Controls and Placebo Effects

The nurturing human touch inherent in massage raises challenges in creating appropriate control conditions. Participants usually know if they are receiving an authentic massage or a minimal/sham intervention, which can introduce expectation effects that alter brain activity (for example, simply believing one will relax can activate the frontal cortex and PAG in a placebo-analgesia manner). To disentangle specific neuroplastic effects of massage from general placebo or nonspecific effects, future trials should incorporate control groups such as light touch (to control for human contact), mechanical massage devices (to control for human interaction while delivering touch), or relaxation training without touch. Some studies have begun using mechanical massage chairs or devices as comparators; interestingly, findings indicate that hands-on massage produces more substantial and more sustained brain effects than mechanized or sham massage, underlining the importance of therapist-administered touch. Nevertheless, careful control design and possibly blinded outcome assessments (where feasible in neuroimaging analysis) are needed to validate that observed longitudinal changes are genuinely due to the massage component.

#### 5.5.3. Longitudinal Study Design

Capturing neuroplastic changes requires measurements across time, but longitudinal neuroimaging is resource-intensive. Many of the studies reviewed had small sample sizes and short follow-up windows. Future research should aim for larger, adequately powered longitudinal designs, potentially piggybacking on clinical trials of massage. For instance, if a trial is administering 8 weeks of massage for fibromyalgia, incorporating pre- and post-intervention fMRI or EEG (and perhaps mid-point measures to track the trajectory) would be invaluable. Repeated-measures EEG or fNIRS could be beneficial due to their lower cost and portability, enabling more frequent monitoring (e.g., weekly EEG to see progressive changes). Another design challenge is accounting for learning and habituation—as we saw, the brain’s response in the 1st session can differ from the 10th. Researchers should also include follow-up scans after a period of no massage to test how durable the changes are once treatment stops. This could reveal, for example, whether the brain gradually returns to baseline (indicating a need for “maintenance” sessions to preserve plastic changes) or whether certain gains are retained long-term.

#### 5.5.4. Multimodal and Convergent Measures

Because neuroplasticity is a multi-level phenomenon, combining methods can provide a more complete picture. Future studies might integrate multimodal neuroimaging—for example, concurrent EEG–fNIRS during massage over a course of sessions to link hemodynamic changes with oscillatory activity, or sequential EEG and fMRI assessments to validate that EEG markers of relaxation correlate with fMRI connectivity changes in key networks. Incorporating peripheral biomarkers of plasticity is also a promising direction. Measuring blood levels of BDNF, oxytocin, inflammatory cytokines, or endocannabinoids before and after a massage regimen can help connect the dots between central and systemic changes. One intriguing area is epigenetics: longitudinal massage studies could examine whether gene expression patterns (perhaps in leukocytes) shift in ways consistent with reduced stress or enhanced synaptic growth—extending the animal findings of touch altering gene methylation into human populations.

#### 5.5.5. Targeting Specific Populations and Windows

Research should also explore which populations benefit most from massage-induced neuroplasticity. It might be that specific critical periods or conditions have exceptionally high payoff—for example, infants and children (as their brains are rapidly developing) or older adults (where interventions might stave off decline). Likewise, individuals with heightened neural plasticity due to pathology (such as after an injury, when the brain is remapping) could be prime candidates. Understanding the individual differences—why some people show robust brain changes with massage and others less so—will be important. Genetics, baseline touch aversion, or microbiome/hormonal milieu differences might contribute and could be studied to personalize massage therapy approaches.

#### 5.5.6. Technological Innovations

The field can leverage new technology to enhance both massage delivery and measurement of its effects. Wearable EEG or near-infrared devices could monitor brain changes in real time during massage in naturalistic settings (e.g., at a massage clinic or patient’s home), providing more ecologically valid data than lab-based scans. This could also enable neurofeedback paradigms, where a therapist might adjust their technique in response to the client’s real-time physiological signals (for instance, aiming to maximize alpha oscillations or oxygenation in the prefrontal cortex). Robotic massage devices, while perhaps less effective alone, could be used in research to deliver highly consistent stimulation patterns and even to stimulate during fMRI (where a human cannot be present), allowing precise control of variables like pressure and rhythm. On the analysis side, machine learning is poised to find latent patterns in complex neuroimaging data; as shown by the successful EEG classification of pain patients’ pre-/post-massage states, advanced algorithms might predict which patients are most likely to experience positive neuroplastic change from massage or identify a signature of “therapeutic brain state” that can be targeted.

## 6. Methodological Considerations and Future Directions

While the current body of evidence is encouraging, our review also highlighted significant methodological variability and gaps. To fully harness massage’s neurophysiological insights, future research must address the following issues.

### 6.1. Heterogeneity of Massage Interventions

Massage therapy is not a single, standardized treatment—it encompasses a wide range of techniques (Swedish, Thai, shiatsu, tuina, myofascial release, etc.) applied to different body sites with varying pressure, speed, and duration. Across the studies we reviewed, there was considerable variability in how massages were delivered, and crucial dose parameters (e.g., force in Newtons, stroke frequency, exact session length) were often not reported in objective units. This heterogeneity makes it difficult to compare results or derive any “dose–response” relationship for massage effects. Standardization is needed: researchers should strive to quantify and report massage parameters (pressure, stroke rate, session duration, number of sessions) in a consistent way, and future studies could systematically vary these parameters to determine optimal dosing. Developing a more uniform framework for describing massage interventions will help differentiate whether, for example, a 15 min moderate-pressure back massage has a different neural impact than a 60 min gentle foot massage. By reducing inter-study variability in intervention reporting, we can better aggregate evidence and establish guidelines for effective practice.

### 6.2. Variability in Outcome Measures

Just as the interventions varied, so did the neuroimaging outcomes assessed. Some studies focused on EEG band power or coherence changes, others on fMRI BOLD activations or resting connectivity, and still others on fNIRS-derived hemoglobin signals. Each modality captures a different facet of brain activity, and even within a modality, there are diverse metrics (e.g., different EEG connectivity or entropy measures). As a result, there is a challenge in harmonizing outcomes—one cannot directly compare an “alpha power increase” to a “BOLD signal change” without a unifying framework. This lack of common outcome metrics makes it hard to synthesize findings or compute effect sizes across studies quantitatively. Going forward, the field would benefit from consensus on key neurophysiological endpoints for massage research. For example, researchers might agree to track specific canonical measures (such as frontal alpha asymmetry, insula or prefrontal activation, heart rate variability, etc.) across studies to facilitate comparisons. Another strategy is to employ multimodal endpoints—for instance, simultaneously recording EEG and fNIRS—so that one study yields an integrated dataset bridging electrical and hemodynamic effects. Establishing more uniform and comprehensive outcome measures will improve our ability to draw general conclusions and build predictive models of massage’s impact.

### 6.3. Sample Sizes and Study Design Limitations

A notable proportion of the studies in this domain had small sample sizes (often <30 participants, sometimes even single-digit pilot studies) and/or lacked robust control groups. Small-n studies are prone to false positives and may not represent the broader population due to sampling variation. Additionally, many experiments were one-off sessions in healthy adults, which, while informative about the mechanism, do not tell us about long-term clinical efficacy. To advance the science, larger randomized controlled trials (RCTs) and well-powered crossover studies are needed. These should include adequate control conditions (such as light-touch placebo, wait-list, or other active comparators) to account for expectation effects and nonspecific benefits. It is inherently challenging to blind participants to massage, since people can feel whether they have received an authentic massage or not, and this challenge must be acknowledged. Creative study designs—for example, comparing massage to a credible alternative relaxation intervention or using blinded assessors for outcomes—can help mitigate bias. Overall, increasing sample sizes and rigor will ensure the reliability of reported neuroimaging effects and support stronger clinical claims.

### 6.4. Longitudinal and Cumulative Effects

Many current studies examine only the immediate brain changes from a single massage session. However, as our review suggests, some of the most interesting effects of massage are cumulative—emerging over multiple sessions (e.g., weeks of therapy) and potentially enduring after the treatment ends. Capturing these longer-term, neuroplastic changes requires longitudinal designs, which have been relatively scarce due to the greater cost and complexity involved. Future research should invest in longitudinal tracking of brain and behavioral changes induced by massage. For instance, a study could use EEG or fMRI to measure participants before therapy, after an acute session, and then again after a 4–8 week course of regular massages, with a follow-up some weeks later to test durability. Such designs would reveal whether initial neural responses grow or diminish with repeated exposure, how long benefits last post-treatment, and whether any “training” of the brain occurs. Long-term follow-ups are crucial for assessing whether massage can induce lasting neuroplastic adaptations (e.g., sustained changes in network connectivity or stress reactivity) that correlate with lasting clinical improvements. Addressing this gap will move the field beyond viewing massage as a transient influence to understanding it as a potential long-term modifier of brain function.

### 6.5. Multimodal Approaches and Convergent Validation

Given that no single neuroimaging modality provides a complete picture, combining modalities can yield a more holistic understanding of massage’s effects. Each technique has strengths: EEG captures millisecond-by-millisecond dynamics of neural activity, fMRI pinpoints deeper brain structures and networks, and fNIRS offers a portable means to measure cortical blood flow changes. Using them in concert can validate findings and uncover interactions (for example, how EEG oscillation changes correspond to blood oxygenation changes in specific regions). A few pioneering studies have begun using concurrent EEG–fNIRS recordings during massage-like interventions, allowing researchers to link fast neural oscillations with slower hemodynamic responses in the prefrontal cortex. Similarly, future studies could acquire EEG inside an MRI scanner (with proper protocols) to directly correlate electrical and BOLD responses to massage stimuli. Multimodal data not only strengthen confidence in results (through cross-verification) but could also help identify biomarkers of massage’s effects—for instance, a characteristic EEG pattern that consistently predicts an fMRI-measured reduction in amygdala activity or an increase in functional connectivity within soothing networks. Embracing multimodal designs and data fusion will accelerate the discovery of such robust markers and deepen our mechanistic insight.

### 6.6. Toward Defining Biomarkers and Personalized Protocols

An ultimate goal for the field is to identify reliable neural or physiological biomarkers that indicate when a person is responding to massage and to tailor massage techniques for individual needs. The current evidence hints at candidates—for example, a substantial increase in alpha oscillations or a leftward FAA shift could serve as an objective signature of relaxation response in the brain, while a reduction in insula or amygdala activity might signal reduced pain/stress processing. By collecting larger datasets and using modern analytics (machine learning, network analysis), researchers can begin to pinpoint which of these signals (or combinations of signals) best predict the desired clinical outcomes (pain relief, anxiety reduction, etc.). This would enable personalized massage therapy, where clinicians could adjust massage pressure, location, or frequency based on real-time feedback from wearable EEG or fNIRS devices. Indeed, emerging technologies are making this plausible: lightweight, wireless EEG and fNIRS systems now allow brain monitoring in naturalistic environments. Future studies could deploy such tools to monitor patients during massage sessions in clinics or at home, providing data on how the brain responds in real-world settings. By integrating technological innovation with neuroimaging, the field can move toward evidence-based, individualized massage protocols and objectively track improvements via biomarker changes.

## 7. Conclusions

Massage therapy’s effects on the brain are not limited to momentary feelings of relaxation—they encompass a spectrum from immediate neural responses to longer-term rearrangements of brain function. The reviewed studies suggest that massage may rapidly shift the brain into a relaxed, internally focused state characterized by increased alpha oscillations, decreased high-frequency activity, and engagement of reward and default-mode networks. These acute changes correlate with reductions in stress and pain. More importantly, when massage is applied repeatedly over time, it may contribute to enduring neuroplastic changes: neural circuits for pain modulation strengthen, aberrant activity patterns associated with chronic conditions move toward normal, and developing brains show accelerated activity maturation. The evidence for such plasticity comes from diverse sources—infants whose EEG development is boosted by early touch, adults whose dysfunctional connectivity is restored by weeks of therapy, and biochemical signs like rising BDNF that point to synaptic remodeling.

The concept of “massage as medicine for the brain” emerges from this synthesis. Massage taps into a web of neurobiological mechanisms—peripheral nerve activation, spinal modulation (gate control), autonomic and endocrine shifts, and cortical network reorganization—by mechanically stimulating the body in a nurturing context. Over the short term, these mechanisms induce beneficial states, and over the long term, they can produce trait-like changes (e.g., a more quiescent stress response or a more integrated default-mode network). This bidirectional influence (body on brain and brain on body) underscores that massage is a truly holistic intervention. It also aligns with prevailing neuroscience models that emphasize brain-body coupling and the role of interoceptive feedback in emotional and cognitive health.

In practical terms, the neuroplasticity of massage means that the benefits of massage can accumulate. Patients and clients who incorporate regular massage into their routine may experience progressive improvements in symptoms and well-being that mirror underlying brain changes. It also means that one should not be discouraged if a single session’s effects are fleeting—like physical exercise for muscles, the neurological “exercise” of massage yields greater endurance and change with training over time. These findings provide a rationale for considering massage as part of chronic management and preventive care (beyond acute relief), though further validation is needed.

Nonetheless, our understanding of massage’s long-term neural impacts is still evolving. While growing, the current body of neuroimaging literature is relatively young and sometimes limited by small sample sizes or disparate methods. Continued research is needed to map out the specific brain changes associated with different massage modalities and how they relate to clinical outcomes. It will be essential to identify which neural changes are most critical—for instance, is it the rebalancing of large-scale networks like the DMN and salience network, the upregulation of descending analgesic pathways, the enhancement of sensory cortex representations, or a combination of many minor adjustments across the brain? Answering such questions will deepen scientific knowledge and guide practitioners in tailoring massage techniques to desired outcomes (for example, slow, gentle stroking to target emotional calming via insula pathways vs. deep tissue work to target sensorimotor integration).

## Figures and Tables

**Table 1:** EEG studies included in the review.

Adult and General Population						
Other Notable Outcomes/Notes	Main EEG Effects (Post-Intervention)	EEG Metrics	Design and Dose	N (Groups) and Sample	Modality/Site	Study
No sex/hemisphere effects; context-dependent modulation (relaxed EC; flexible EO)	EC: ↑ synchronicity F–C (LH); EO: ↓ synchronicity C–O (RH)	21-ch; cross-correlation (synchronicity)	Within-subject; 20 min; EC and EO	40 healthy (20–25 y)	Combined general + point massage, cervical-collar	[20]
s-IgA ↔; pulse ↓ (esp. G2, oil); SpO_2_ slight ↓ (relaxation)	α: ↑ in G1–G2 (aroma); β: ↑ in G1 (oil and aroma-oil); θ: ↑ esp. G3 (aroma/oil)	α, β, θ power; ratios	Multiple sessions × 5 min each; pre/post	28 (G1 adults; G2 older no care; G3 older with care)	Oil massage, aromatherapy (lavender inhalation), aroma-oil massage	[21]
Local contralateral inhibition → generalized relaxed wakefulness	δ, θ: ↓ (bilateral F/FC/C); α: ↑ (same regions); β: ↓ at C4 during left-arm massage	δ, θ, α, β absolute power	Within-subject; 25 min/side	18 healthy	Unilateral Swedish massage (arm/face/neck)	[22]
Supports parasympathetic shift/relaxation	α: ↑; δ: ↑ (vs baseline and control)	Absolute and relative δ, θ, α, β	RCT vs. rest; 15 min	26 stressed students	Manual lymph drainage (MLD), neck	[23]
MLD produced a stronger calming effect	Both: α ↑, β/γ ↓; MLD > AM for α ↑ and γ ↓	α, β, γ (abs/rel)	Parallel groups; 20 min	28 stressed	MLD abdomen vs. conventional abdominal massage	[24]
Suggests a more positive effect	Shift toward left-frontal activation; F7–F8 significant (*p* = 0.032)	Alpha asymmetry (Fp1–Fp2, F3–F4, F7–F8)	Pre/post; 15 min	13 stressed females	MLD, neck (frontal asymmetry)	[25]
Manual more relaxing/stable connectivity; mechanical more stimulating	Hands-on: δ/θ ↑; Mechanical: δ/θ ↓, β ↑; α ↓ esp. mechanical; Mechanical ↓ coherence (α/β)	32-ch power (δ–β), coherence, α asymmetry	Between-groups: 3 min/point × 4	24 healthy	Hands-on vs. mechanical massage at acupoints	[26]
Interpreted as cortical deactivation/rest; specific vs. controls	After facial massage: α ↓ (attenuation), β ↓, slight θ ↑; O1–O2 α-coherence ↓	Fz, O1, O2; α, θ, β; coherence	20 min; 3-arm	24 females (19–21 y)	Facial (esthetic) massage vs. autogenic training vs. rest	[27]
Math speed/accuracy ↑; anxiety ↓ (massage only); cortisol ↓ after first session	Both: frontal δ ↑; Massage only: α ↓, β ↓	Frontal δ, θ, α, β-low, β-high	RCT; 2×/wk × 5 wks	50 adults	Chair massage vs. guided relaxation (5-wk)	[28]
Anxiety/depression/stress ↓; salivary cortisol ↓ acutely; plasma BDNF ↑ over 4 wks	Single session: α ↑ (17%→25%), δ ↓; acute EEG effects attenuated by session 8; no baseline shift	α, δ (acute pre/post each session)	40 min, 2×/wk × 4 wks	25 mothers of children with ADHD	Aromatherapy massage (lavender + geranium)	[29]
HR ↓ (moderate), HR ↑ (light/vibratory); stress/anxiety ↓ most with moderate	Moderate: δ ↑, α/β ↓, left-frontal shift; Light: δ ↓, θ/β ↑; Vibratory: θ/α/β ↑	δ, θ, α, β-low, β-high; frontal asymmetry	10 min, 3 arms	36 adults	Moderate- vs. light-pressure massage vs. vibratory	[30]
Indicates alert cortical activation	β and γ (31–80 Hz) ↑; δ ↓; left-frontal emphasis	Frontal δ–β; γ1–γ3	Pre/post; ~10 min total	7 healthy males	Foot reflexotherapy	[31]
HR, RR ↓; mood ↑; cortisol/catecholamines ↔	α ↑ (relaxed alertness)	α power	Single 45 min session	16 adults (30–60 y)	Shirodhara (after full-body Abhyanga)	[32]
FR ↓ skin conductance; MM > FR for pain/muscle relaxation; ROM ↑ (toe-touch best with MM)	FR and MM: ↑ α/β sync (FR frontal; MM parietal); AT: desynchronization	Resting α and β synchronization	RCT; single bout	65 adults	Foam rolling (FR) vs. manual massage (MM) vs. autogenic training	[33]
Suggests reduced pain/altered connectivity; ML models classified states well	Alpha-band complexity (SampEn, PermuEn) ↓ across channels; PDI changes (β)	SampEn, PermuEn; CSP; PDI	Pre/post; 30 min	71 patients (mean 54 y)	Clinical massage in skeletal muscle pain (EEG complexity)	[36]
LDH: relaxation/analgesia; Healthy: relaxed-alert; PDI ↑ (LDH), ↓ (healthy)	LDH: δ entropy ↓ & δ energy ↑ (LH); θ/α energy ↓; Healthy: δ entropy ↑; α/β complexity ↓	Entropy (ApEn, SampEn, WaveEn, PE, FuzzyEn, IFE), energy	Pre/post; 15 min	26 LDH; 24 healthy	Chinese massage in LDH patients vs. healthy	[37]
Small pilot; suggests relaxation	α ↑ significantly (μV 1.60→2.47); other bands ↔	NeuroSky bands	Pre/post; 30 min	5 adults	Machine-assisted shoulder massage	[38]
Both rated pleasant/relaxing/refreshing	Hand: ↑ α in left insula; Foot: ↑ α in bilateral PCC	eLORETA α sources	15 min each; 1-week washout	12 (mean ~82 y), crossover	Hand vs. foot massage in elderly LTC	[39]
Confirms robot can elicit “massage-like” brain responses	δ ↑; α ↓ (F3/F4)	δ, α, β (14-ch)	Baseline → 10 min massage → post	7 adults	Dual-arm robotic massage (press/rub/stroke)	[40]
Attention benefits sustained; verbal STM and LTM ↑ only with Massage + BB	Only Massage + BB: ↓ θ/β & ↓ (θ + α)/β across sites	Prefrontal θ, α, β; fatigue ratios θ/β, (θ + α)/β	20 min conditions A/B/C	25 adults; crossover	Massage chair ± binaural beats (“brain massage”) vs. rest	[41]
HR ↓; respiration ↑; HRV ↔; strong positive subjective effects	Global power ↓ during/after; largest ↓ in β2 and γ	Global PSD: δ–γ	20 min; before/during/after	34 adults	Singing bowl (“sound”) massage	[43]
Anxiety and pain ↓ more with TTM; EEG changes track symptom relief	TTM: δ ↑; θ/α/β ↓; PT: minimal change	δ–β power	Single 30 min session	40 patients	Traditional Thai massage vs. PT (SCS pain)	[44]
COVI anxiety improved in responders	In high-β users, massage ↓ β amplitude (3/4 responders)	Frontal β amplitude	Two-stage test; real-time	10 adults	EEG-triggered massage headband (prototype)	[45]
Interpreted as reduced cortical complexity with pain relief	Overall entropy ↓; δ and α ApEn ↓; β HHTMSEn ↓	ApEn, HHTMSEn; δ–β bands	Pre/post; 25 min	26 SLBP patients	Massage in specific low back pain (EEG entropy)	[46]
Both reduced leg swelling; hand rated more pleasant/relaxing	Hand: ↑ α in left ACC; Machine: no α source change	eLORETA α sources	15 min; 2-week washout	18 women; crossover	Hand vs. machine leg massage (occupational swelling)	[47]
Pediatric and Neonatal Populations						
Spectra closer to term-born profiles with higher dose	Primary β GRP ↔; ↑ central α RP; dose–response: more sessions → ↑ β/α/θ RP, ↓ δ RP; per-protocol: β and α GRP ↑	EEG GRP (β primary), α, θ, δ	From 34 wks GA to term; 15 min, 2×/day	60 VPT; RCT	Maternal–infant massage (very preterm)	[34]
Suggests preservation/enhancement of neurophysiologic activity	Massage group avoided global decline; ↑ δ and θ (central), ↓ temporal δ/α; time × group effects for global δ, central δ/β	Spectral δ, θ, α, β; regional	Randomized; 3×/day × 10 days	20 preterm	Infant massage (preterm)	[35]
HR ↓; novelty/dynamic patterns evoked stronger calming	School-age: PAF ↓ (relaxation); Preschool: FD ↓ during some stories	Spectral power, Peak Alpha Frequency (PAF), fractal dimension; HR	Within-subject	Preschool (~5.5 y) and school-age (~8.7 y)	Playful back “story” massages (children)	[42]
No parietal change; aligns with improved affect risk profile	Right-frontal asymmetry ↓ during and after (toward balance/left)	Frontal/parietal (3–13 Hz) asymmetry	Within-session pre/during/post; 10 min	25~1.2-mo infants	Infant massage (infants of depressed mothers)	[48]
Feasible aEEG neuromonitoring for tactile interventions	More continuous backgrounds in the massage group; fewer delayed maturation scores	aEEG continuity; Burdjalov maturation	RCT subset; twice-daily; 12 h aEEG at 34 PMA	26 VLBW infants	NICU massage (VLBW), aEEG maturity	[49]

Abbreviations: EEG = electroencephalography; EC = eyes closed; EO = eyes open; F/C/P/T/O = frontal/central/parietal/temporal/occipital; LH/RH = left/right hemisphere; δ/θ/α/β/γ = delta/theta/alpha/beta/gamma; GRP = global relative power; RP = relative power; aEEG = amplitude-integrated EEG; PSD = power spectral density; 10–20 = international EEG electrode placement system; ACC = anterior cingulate cortex; PCC = posterior cingulate cortex; PAF = peak alpha frequency; FD = fractal dimension; HR = heart rate; HRV = heart rate variability; ROM = range of motion; RCT = randomized controlled trial; PT = physical therapy; TTM = traditional Thai massage; MLD = manual lymph drainage; AM = abdominal massage; FR = foam rolling; MM = manual massage; AT = autogenic training; BB = binaural beats; STAI = State–Trait Anxiety Inventory; BDI = Beck Depression Inventory; POMS = Profile of Mood States; VAS = Visual Analog Scale; MAS = Mood Assessment Scale; SRI = Stress Response Inventory; s-IgA = salivary immunoglobulin A; SpO_2_ = peripheral oxygen saturation; BDNF = brain-derived neurotrophic factor; DMN = default-mode network; ERP = event-related potential; P1/P2 = early visual ERP components; LPP = late positive potential; ApEn = approximate entropy; SampEn = sample entropy; PermuEn = permutation entropy; WaveEn = wavelet entropy; HHTMSEn = Hilbert–Huang transform marginal spectrum entropy; IFE = inherent fuzzy entropy; PE = permutation entropy; PDI = permutation disalignment index; CSP = common spatial pattern; ML = machine learning; VPT = very preterm; VLBW = very low birth weight; NICU = neonatal intensive care unit; GA = gestational age; PMA = postmenstrual age; TEA = term-equivalent age.

**Table 2:** fMRI studies included in the review.

Key Takeaway	Behavioral/Clinical	Main Neural Findings	Primary ROIs/Network	Analysis	fMRI Paradigm	Control/Comparator	Intervention/Touch	Population	Study
Human touch (Swedish) most strongly modulated DMN at rest; task engagement reduces sACC effect.	Small ↑ well-being across groups (ns); baseline positive affect higher in Swedish (covaried)	Swedish ↑ BOLD in sACC and RSC/PCC at rest; the sACC effect vanished during the task. Reflexology mainly ↑ RSC/PCC at rest; object ↑ RSC/PCC during task.	DMN: sACC, RSC/PCC	SPM8 GLM; whole-brain; ROI sACC, RSC/PCC; FWE-corrected	Rest + Go/No-Go task; right-foot-stimulated	Rest; wooden object vs. human touch	Swedish massage; Reflexology; Wooden object (tactile control); Rest	40 healthy (mean 32.3), 4 arms; right-handed	[50]
Somatosensory responses to reflexology are robust to cognitive expectation; unusual left-lateralized face-area activation.	-	Consistent activation of left middle postcentral gyrus (face/eye) and contralateral foot SI; unaffected by pseudo-info.	SI (postcentral gyrus) face/eye area; foot area	SPM8; 2 × 2 RM-ANOVA; SI mask; FWE-corrected	Reflex-area stimulation (toes) during fMRI	Misinformation manipulation	Reflexology to the eye reflex area; correct vs. pseudo-information	32 healthy Japanese (mean 22.2)	[51]
Reflex-area stimulation evokes somatotopically plausible SI patterns; partial support for reflexology mapping.	-	Eye → left mid postcentral (face/eye); SI → left superior postcentral (trunk). Shoulder: trend in right postcentral (ns). Common foot-related SI activation.	SI somatotopy (postcentral gyrus)	SPM2; subtraction contrasts; AAL mask; FWE-corrected	Block stimulation of reflex areas	Within-subject contrasts	Left-foot reflex areas: eye, shoulder, small intestine (SI); wooden stick	25 healthy (mean 22), right-handed	[52]
Skin-to-skin dynamic touch uniquely recruits pgACC (reward/affect) beyond generic tactile processing.	Pleasantness: Human moving > Human static > Glove moving > Glove static	Moving touch → bilateral insula and S1/S2; Human + moving uniquely → pgACC.	Insula, S1/S2, pgACC	SPM; whole-brain	During-touch BOLD; pleasantness ratings	Rubber glove; stationary	Forearm touch: human vs. rubber glove × moving vs. stationary (2.5 N; 1.5 cm/s)	16 healthy (mean 30.2)	[53]
Brief lower-limb massage alters RS network topology—trend toward hypo-integration consistent with relaxation.	-	Post-massage: experimental group showed reduced integration vs. controls in subnetwork incl. ant pulvinar thalamus and pgACC.	Thalamus (ant pulvinar), pgACC; broader network	SPM12; AAL3 ROI network metrics (NS, BC, EC, CC, efficiency)	Pre/post resting-state	Rest (no massage)	5 min calf-and-foot massage	27 massage; 11 rest controls	[54]
Tactile foot stimulation modulates intrinsic networks via nonspecific touch/relaxation effects; proposed pain-related NNCP.	HR ↓; well-being ↑ similarly for FR and SM; no adverse events	Both FR and SM: ↓ DMN PCC/precuneus connectivity; ↑ SMN connectivity; NNCP mixed modulations; no FR > SM specificity.	DMN (PCC/precuneus), SMN, SN, ECN, NNCP	CONN toolbox; ROI-to-ROI in DMN, SMN, SN, ECN; NNCP (23 ROIs)	Pre/post rs-fMRI; physiological and well-being	Within-subject FR vs. SM; double-blind	Foot Reflexology (FR) vs. Sham Massage (SM), 10 min (left foot)	30 healthy (~30 y), crossover	[55]
Reflexology robustly engages primary sensorimotor cortices; broader activations may relate to relaxation/stress pathways.	No cessation outcomes tested	Consistent contralateral pre/postcentral activation; variable extra-cortical activations; no major smoker vs. non-smoker differences.	Motor (precentral) and sensory (postcentral); thalamus	GLM; whole-brain	Task fMRI during reflexology	Smoker vs. non-smoker groups	Foot reflexology at three hallux points (45 s each; alternating feet)	20 males (15 smokers, 5 nonsmokers)	[56]
Oxytocin amplifies hedonic and social-reward processing of human-delivered touch; imagery evokes similar but motor-sparser patterns.	Pleasantness ↑ with OT (manual only); gender of masseur has no effect	OT ↑ pleasantness & ↑ activation for manual massage across reward, salience, social, DMN; minimal effect for machine.	Reward (OFC, striatum, VTA), Salience (amygdala, ACC, insula), Social (STS, IPL), DMN (mPFC, PCC, precuneus)	SPM12; whole-brain; FDR-corrected	Task fMRI during massage/imagery; pleasantness ratings	Placebo; machine; imagined	Intranasal oxytocin vs. placebo; manual vs. machine foot massage; real and imagined	46 healthy males (mean 21.2), within-subject	[57]
Tuina modulates regional synchrony in pain/DMN regions alongside clinical improvement in cervical spondylosis.	Pain and disability ↓; ReHo changes correlated with symptoms	Pre: abnormal ReHo (↑ ACC/PCC/thalamus; ↓ right gyrus rectus). Post: ReHo shifts incl. ↑ left inf occipital; persistent ↓ right rectus.	DMN nodes (ACC/PCC), thalamus, temporal, inferior parietal; right gyrus rectus	DPARSF; ReHo (KCC); correlations with symptoms	rs-fMRI; ReHo; symptom scales (VAS, NDI)	HC baseline; pre vs. post within CS	Tuina (6 sessions/2 weeks)	27 CS patients (mean 37.3) + 27 HCs	[58]
Tuina appears to restore DMN-related dysfunction in LDH; rs-fMRI metrics track symptom relief.	Pain and disability ↓; neural changes correlated with clinical gains	Pre: ↓ ReHo in LO-MFG; abnormal dFC variance. Post tuina: ↑ ReHo LO-MFG; dFC variances normalized toward HCs.	DMN (LO-MFG/mPFC, precuneus), fusiform, IFG	RESTplus; seeds from ReHo; sFC and dFC (sliding window)	rs-fMRI; ReHo + static and dynamic FC; symptoms (VAS, C-SFODI)	HC baseline; pre vs. post within LDH	Tuina (6 sessions/2 weeks)	27 LDH patients + 28 HCs	[59]
Different MTs rapidly reconfigure pain-related FC, with some modality-specific signatures despite similar pain relief.	Pain ↓ across all; PPT ns; weak brain–behavior correlations	Shared: PCC–aINS shift to +FC; pINS–PAG ↑; SI(R)–pINS(L) ↓. Technique-specific FC changes (e.g., SMT ↑ SI–aINS).	Pain-processing network; descending modulation (PAG)	SPM12 + CONN; 16 ROIs (SI/SII, THA, ACC/PCC, aINS/pINS, PAG)	Pre/post rs-fMRI; ROI-to-ROI FC; pain and PPT	Between-technique comparison	Spinal manipulation (SMT), mobilization (MOB), therapeutic touch (TT)	24 healthy (exercise-induced myalgia) → SMT *n* = 6, MOB *n* = 8, TT *n* = 10	[60]
OMT selectively tunes interoceptive networks and improves interoceptive accuracy in CLBP.	Improved heartbeat tracking in OMT; EA unchanged	After first session: slight ↑ activation (except rMFG ↓); by T2: marked ↓ activation vs. baseline and sham—suggesting efficiency gains.	Insula (bilat), ACC, left striatum, right MFG; salience/interoceptive circuits	AFNI GLM; MVM; independent ROI analysis	Tasks: Interoception (heartbeat) and exteroception (sound); fMRI at T0, T1, T2; heartbeat tracking	Randomized, placebo-controlled	Osteopathic manipulative treatment (4 weekly) vs. sham	29 CLBP (OMT *n* = 15; Sham *n* = 14)	[61]

Abbreviations: AAL—Automated Anatomical Labeling atlas; AAL3—updated AAL brain parcelation; ACC—anterior cingulate cortex (salience/affect, pain modulation); AFNI—Analysis of Functional NeuroImages (fMRI software); aINS—anterior insula (interoception/salience); BC—betweenness centrality (graph metric); BOLD—blood oxygen level–dependent signal (fMRI contrast); CC—clustering coefficient (graph metric); CLBP—chronic low back pain; CONN—connectivity analysis toolbox for fMRI; C-SFODI—Chinese Short Form Oswestry Disability Index (back-pain disability); dFC—dynamic functional connectivity (time-varying FC); DMN—default-mode network (self-referential/affect, rest); EC—eigenvector centrality (node influence in graphs); ECN—executive control network (cognitive control); EPI—echo-planar imaging (fast fMRI sequence); FDR—false discovery rate (multiple-comparison control); FC—functional connectivity (correlated BOLD between regions); FWE—family-wise error (multiple-comparison correction); FR—foot reflexology; GLM—general linear model (voxel-wise stats); HR—heart rate; IA—interoceptive awareness (internal-body signals); IFG—inferior frontal gyrus (control/language); Insula—insular cortex (affect/interoception); IPL—inferior parietal lobule (multisensory/social); MNI—Montreal Neurological Institute space (standard coords); MOB—spinal mobilization (low-velocity manual therapy); MVM—multivariate modeling in AFNI (3dMVM); mPFC—medial prefrontal cortex (DMN/valuation); NDI—Neck Disability Index; NNCP—Neural Network Correlates of Pain (proposed ROI set); NS—node strength (sum of edge weights); OFC—orbitofrontal cortex (reward/valuation); OMT—osteopathic manipulative treatment; OT—oxytocin (neuropeptide); PAG—periaqueductal gray (descending pain modulation); PCC—posterior cingulate cortex (DMN hub); pgACC—pregenual anterior cingulate (pleasantness/reward); Postcentral—postcentral gyrus (primary somatosensory cortex); Precuneus—posterior medial cortex (DMN); Precentral—precentral gyrus (primary motor cortex); PPT—pressure pain threshold; ReHo—regional homogeneity (local BOLD synchrony); RSC/PCC—retrosplenial/posterior cingulate cortex (posterior DMN); rMFG—right middle frontal gyrus (attention/control); RM-ANOVA—repeated-measures ANOVA; ROI—region of interest; ROI-to-ROI—connectivity between predefined ROIs; rs-fMRI—resting-state fMRI; sACC—subgenual anterior cingulate cortex (affect/DMN); SI—primary somatosensory cortex; SI/SII—primary/secondary somatosensory cortices; S1/S2—same as SI/SII; SM—sham massage (control touch); SMN—sensorimotor network; SMT—spinal manipulation therapy (HVLA thrust); SN—salience network (ACC/insula); SPM2—Statistical Parametric Mapping v2; SPM8—Statistical Parametric Mapping v8; Striatum—dorsal/ventral striatum (reward/motor); STS—superior temporal sulcus (social perception); THA—thalamus (abbr.); Thalamus—sensory relay/integration hub; tuina—traditional Chinese manual therapy; VAS—Visual Analog Scale (pain); VTA—ventral tegmental area (dopaminergic reward); fMRI—functional magnetic resonance imaging; pINS—posterior insula (interoceptive/somatosensory integration).

**Table 3:** fNIRS studies included in the review.

Behavioral/Physiological Outcomes	Main Neural Effects	Other Measures	fNIRS Setup (Device • Channels/ROIs)	Protocol/Design	Interventions	Sample	Study
OXT ↑ both: +51.8% hand vs. +18.2% machine; hand rated more pleasurable and higher WTP; intensity/arousal ≈; OXT (baseline and post-hand) negatively correlated with AQ/STQ; mlOFC activation positively correlated with AQ; pSTS/mlOFC ↔ pleasure and WTP	Hand massage ↑ pSTS and mlOFC; machine showed ↓ mlOFC; no S1 difference; mediation: S1 influences mlOFC via pSTS	Plasma oxytocin pre/post; SCR; ratings (pleasure, intensity, arousal, willingness-to-pay); AQ, STQ	Techen CW6; 27 optodes (12 sources/15 detectors), 3.0 cm spacing; 690/830 nm; ROIs: bilateral OFC (lOFC, mlOFC, mOFC), medial S1, bilateral aSTS/pSTS	GLM on 20 s massage blocks with 10 s rests; ROI-based analyses; Bonferroni-corrected; mediation (S1→STS→OFC)	10 min hand vs. machine foot massage (within-subject; order counterbalanced; blindfolded)	40 healthy Chinese males (mean 21.78); 36 analyzed	[62]
Both comfortable; aroma scent clear/pleasant (3/4); relaxation generally greater with aroma	PFC (esp. ch13) ↑ oxy-Hb/total-Hb, deoxy-Hb ↓ for both massages; with aroma: sharper early ↑ then decline; PFC ch8–9 showed wider fluctuations; S1 ch33/35 differed by condition	Perceived strength, comfort, relaxation, scent (5-point Likert)	Shimadzu FOIRE-3000; 46 channels; ROIs: prefrontal (1–22) and somatosensory (23–46)	Alternating R/L foot; 60 s task/20 s rest, ×3 cycles; post-session questionnaires	Footbath; standard massage; footbath + aroma; aroma oil massage (lavender, tea tree, ravensara, palmarosa, chamomile)	4 elderly females (≥65 y)	[63]
fNIRS reveals disrupted sensorimotor lateralization aligned with motor dysfunction; supports neurofunctional specificity of Hegu for rehab monitoring	Less-affected arm in patients: contralesional SM1 dominance (like controls); affected arm: contralateral dominance absent; LI differed (affected vs. less-affected)	-	NirScan-8000A; 11 sources/10 detectors; 730/850 nm; 32 channels; ROIs: SM1 (ipsi/contra), PMC (ipsi/contra), SMA, SAC	Rest 20 s → Tui Na 20 s → Rest 30 s, ×6; GLM; lateralization index (LI)	Tui Na (“one-finger Zen”, ~120/min) at Hegu on both hands	10 unilateral ischemic stroke pts (mean 58 y) + 8 healthy controls (mean 49.25 y), right-handed	[64]
Both feasible; time × intervention effects significant; massage = more gradual/sustained engagement; RLT = rapid/transient activation	Massage: bilateral HbO ↓ (min 1) → ↑ (min 2); L hemi sustained ↑ (min 3); pattern varies through min 5; post: ↑ right and ventral left motor. RLT: sharp bilateral ↑ (min 1) → ↓ (min 2; L > R); rebounds then drops; distinct temporal signatures	-	Cortivision photoncap C20 (Baby kit); bilateral motor cortex coverage; HbO tracked continuously	5 min baseline, intervention (~5 min sequence), 5 min post; mixed ANOVA; Homer3 preprocessing; AtlasViewer mapping	Massage (1 min strokes across body) vs. Reflex Locomotion Therapy (pectoral pressure)	20 full-term infants (pilot n = 2)	[65]
Fewer post-therapy errors; Random Forest top individual (≈94.6%); ensemble best overall; 90% reported improved focus/relaxation	Post-intervention: HbO_2_ ↑, HbR ↓ across PFC → enhanced activation	Behavioral errors; ML metrics (accuracy, precision, recall, F1, AUC)	NIRSport 2; 20 PFC channels (dlPFC, OFC, vmPFC); band-pass 0.05–0.3 Hz; HbO_2_/HbR via MBLL	Pre/post battery: picture recognition, digit span, Stroop, SART, N-back; fNIRS over PFC; ML classification (10 models + ensemble voting)	Heated mechanical foot reflexology (35 °C) + binaural beats (256/240 Hz → 16 Hz beta)	10 healthy adults (5F/5M, 18–30 y)	[66]

Abbreviations: OXT = oxytocin (plasma neurohormone linked to social bonding); fNIRS = functional near-infrared spectroscopy (optical imaging of cortical hemodynamics); HbO/HbO_2_ = oxygenated hemoglobin; HbR = deoxygenated hemoglobin; PFC = prefrontal cortex; OFC = orbitofrontal cortex; lOFC/mlOFC/mOFC = lateral/mediolateral/medial orbitofrontal cortex; S1 = primary somatosensory cortex; SM1 = primary sensorimotor cortex; STS = superior temporal sulcus; aSTS/pSTS = anterior/posterior STS; PMC = premotor cortex; SMA = supplementary motor area; SAC = somatosensory association cortex; dlPFC/vmPFC = dorsolateral/ventromedial prefrontal cortex; GLM = general linear model (task-evoked signal estimation); ROI = region of interest; HRF = hemodynamic response function (canonical shape used in GLM); SCR = skin conductance response (autonomic arousal); AQ = Autism Spectrum Quotient; STQ = Social Touch Questionnaire; WTP = willingness to pay; LI = lateralization index, (contralateral − ipsilateral)/(contralateral + ipsilateral); ipsi/contra = ipsilesional or ipsilateral vs. contralesional or contralateral (relative to lesion/stimulus); R/L = right/left; MBLL = modified Beer–Lambert law (converts optical intensity to HbO/HbR); RLT (Vojta) = reflex locomotion therapy; SART = sustained attention to response task.

## Data Availability

No new data were created or analyzed in this study. Data sharing is not applicable to this article.
